# Creating locally enriched single-atom catalysts via bio-inspired internalization process for interference-resistant NO_2_ detection in complex environments

**DOI:** 10.1038/s41467-026-76850-4

**Published:** 2026-08-20

**Authors:** Xin Jia, Runfang Mao, Shan Hu, Muyu Yan, Haotian Huang, Zhiheng Ma, Xuesong Zhang, Musen Li, Yongjie Shen, Wenqiang Qu, Zhanchen Wang, Yi Jiang, Shuai Guo, Pengcheng Xu, Zhenggang Xue, Dengsong Zhang, Jiaqiang Xu

**Affiliations:** 1 Department of Chemistry, College of Sciences, Shanghai University, Shanghai, China; 2 School of Intelligent Manufacturing, Huzhou College, Huzhou, China; 3 College of Chemistry and Materials Science, Shanghai Normal University, Shanghai, China; 4 State Key Laboratory of Transducer Technology, Shanghai Institute of Microsystem and Information Technology, Chinese Academy of Sciences, Shanghai, China; 5 Institute for Chemical Reaction Design and Discovery (WPI-ICReDD), Hokkaido University, Sapporo, Japan; 6 Department of Chemistry, University of Toronto, Toronto, ON Canada; 7 Shanghai Key Laboratory of Intelligent Manufacturing and Robotics, Shanghai University National-level Experimental Teaching Demonstration Center for Engineering Training, Shanghai University, Shanghai, China

**Keywords:** Sensors and biosensors, Heterogeneous catalysis

## Abstract

Intelligent gas-sensing technology that accurately and stably identifies gas categories in complex atmospheres is critical for protecting public safety and environment. However, competing interactions among different gases on sensing surfaces can trigger interference even poison of sensors. Here, we present a bio-inspired atomic internalization process that produces locally enriched single Pt species, enabling highly selective and interference-resistant gas detection. By engineering Sn/C precursors as homologous receptors, Pt_3_Sn alloys are two-step redistributed into high-density single Pt species within regionalized SnO_2_ surface. This structure constitutionally alters the distribution patterns of NO_2_ molecules and thus delivers accurate NO_2_ monitoring in multicomponent atmospheres and stable detection for over 550 days, surpassing state-of-the-art commercial devices. Further integrating characteristic-specific sensors into arrays, the resulting device further achieves 100% classification accuracy for single and mixed gases at ultralow cost. Moreover, we demonstrate an autonomous “cruise-monitoring” system by equipping the sensor on a robot to detect and identify NO_2_ in real time. Our findings thus guide the accurate analysis and interference-resistant detection in complex gas mixtures.

## Abstract (web summary)

Accurate gas sensing remains challenging because conventional semiconductor sensors often struggle with selectivity and interference from complex gas mixtures. Here, the authors use a single-atom catalyst strategy to stabilize isolated Pt atoms on regionalized SnO₂ surfaces, enabling selective and antiinterference NO₂ detection. The resulting sensor operates continuously in air for more than 550 days and achieves high classification accuracy across a library of 37 gases, highlighting the promise of single-atom-engineered sensors for reliable gas monitoring.

## Introduction

The accurate detection and identification of toxic gases (such as NO_2_) in air are of vital importance in environmental monitoring, energy utilization, and health management^1–6^. Traditionally, commercial detection technologies mainly involve semiconductor-type gas sensors based on tin dioxide (SnO_2_) as the sensing material due to its excellent chemical stability and cost-effectiveness^7,8^. However, in practical detection applications, simple SnO_2_-based sensors are frequently hampered by poor selectivity and susceptibility to interference in complex gas mixtures, leading to functional confusion and misinformation risk^9^. To overcome these limitations, sensor array designs coupled with machine learning algorithms have been developed to leverage the cross-sensitivity of sensors for whole sensing accuracy^10–14^. For example, Fan et al.^15^. reported a biomimetic olfactory chip containing up to 10,000 individually addressable sensors combined with artificial intelligence, offering excellent distinguishability for different gases. However, this strategy often relies on complex data processing routines caused by functionally similar sensor selections and consistent sensing behaviors toward various gases, resulting in high computational power and energy consumption. Hence, integrating characteristic-specific and behavior-distinctive sensors into a system, while simultaneously optimizing the associated algorithms to reduce training costs and enhance decision-making efficiency, is essential for developing highly efficient sensing architectures.

Normally, the efficiency of sensor arrays is restricted by the similar response behaviors of sensing materials toward different gases. Especially under complicated multi-component gas conditions, sensing materials will encounter competitive adsorption and response of different gases, even poisoning process^16–18^, which severely impede the activity, selectivity, and stability of sensors. In this regard, single-atom catalysts (SACs) hold immense promise for resolving the selectivity and interference problems of semiconductor sensors due to their high dispersion and uniform coordination environments^19–22^. This structural nature is inclined to specifically contact and interact with objective gas molecules. Especially, highly dense SACs often exhibit distinctive sensing properties derived from their characteristic interatomic distances, unique gas adsorption patterns, and synergistic catalytic effects^23–26^. However, this distribution mode is extremely rare in gas-sensing applications, considering that the required high operating temperature (typically at 150 °C–400 °C) often induces the formation of nanoparticles and deactivates the sensor. As a result, SACs for gas-sensing applications generally maintain a sparse and scattered distribution to prevent aggregation. In recent years, thermally stable SACs have also been widely studied to meet the demands of high-temperature operating conditions^27–29^. For instance, Li et al.^30^. reported that noble metal nanoparticles (Pd, Pt, Au) can be converted into thermally stable single atoms over carbon supports through strong metal-carbon interactions at 900 °C. However, a prominent challenge is that the dispersion states of migratory metal species at high temperatures are non-directional and difficult to control, which limits the formation of highly dense SACs.

**Fig. 1:**
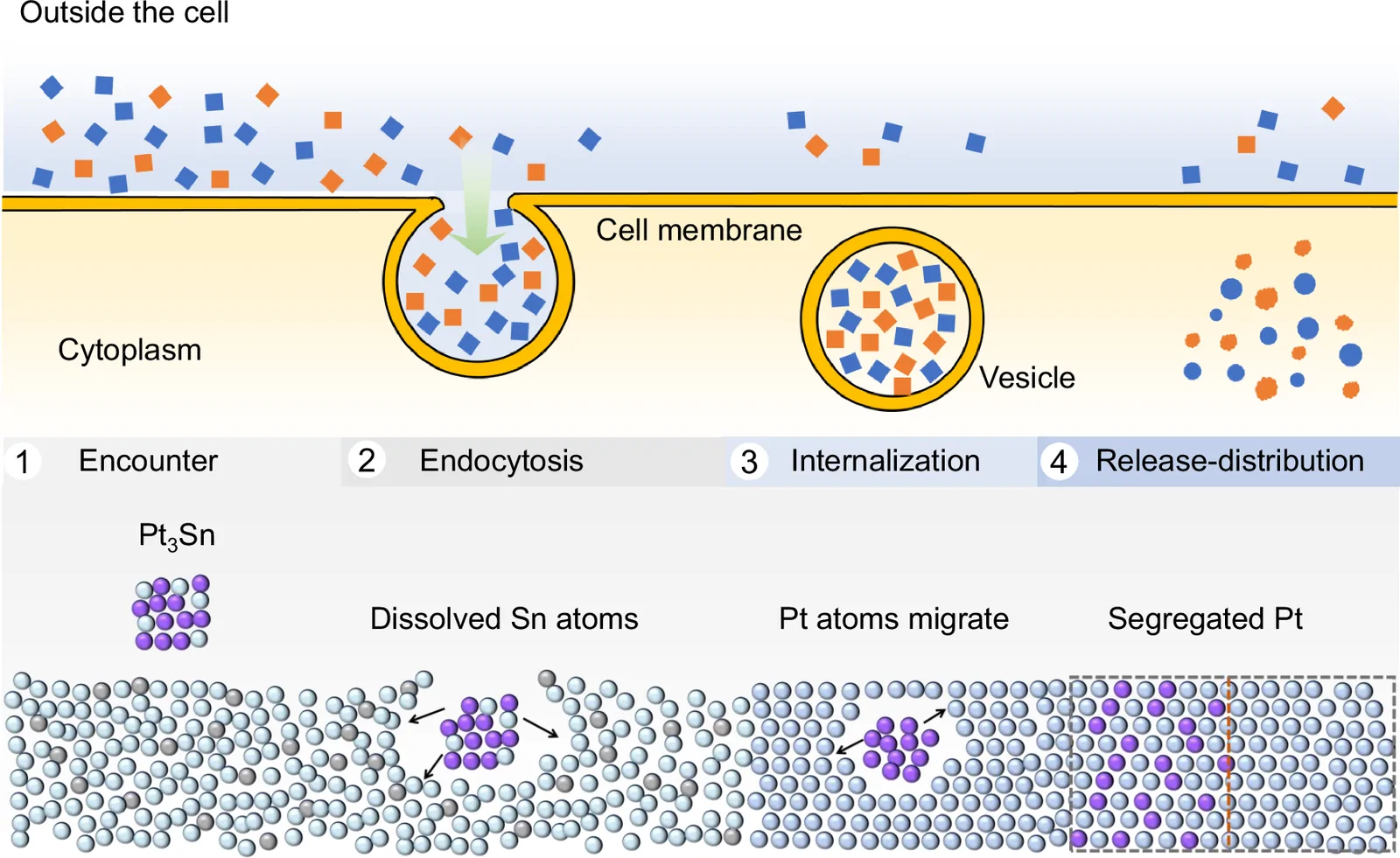
Catalyst design inspired by exogenous endocytosis in biosystems.

To navigate the trade-off between high-temperature stability and high-density dispersion of SACs, inspiration is drawn from exogenous endocytosis in biosystems. As seen in Fig. 1 (top), exogenous endocytosis proceeds through four main steps: 1) encounter of exogenous substances with the cell membrane, 2) invagination of the cell membrane and endocytosis of receptors, 3) formation and internalization of vesicles, 4) release and redistribution of exogenous substances^31,32^. The essence of this process involves “proactive-confine” and “protective-release” mechanisms, where exogenous substances are identified and protected within a narrow space, enabling their controlled release inside the host. This precise modulation avoids the stochastic dispersion of substances and presents a potential solution to achieve localized structural management.

Here, we establish a bio-inspired internalization strategy to guide the dispersion of segregated and thermally stable single Pt species within a regionalized SnO_2_ surface, enabling characteristic-specific and anti-interference NO_2_ detection. As shown in Fig. 1 (bottom), Pt_3_Sn alloy precursors (simulative exogenous substances) are first engulfed by Sn/C supports (simulative homologous receptors) through Sn-Sn interactions at elevated temperature in air. Upon oxidation and coalescence of Sn species, Pt species become localized within specific regions. The aggregated Pt species then re-disperse to form segregated yet highly dense SACs on regionalized SnO_2_ surfaces. The detailed internalization process of the Pt_3_Sn alloy is traced by in-situ transmission electron microscopy (TEM) and molecular dynamics (MD) simulations. The resulting 1%-PtSn-SnO_2_ (1% Pt_3_Sn loading on SnO_2_ calcined in air) sensors exhibit highly selective and interference-resistant NO_2_ detection under dual and ternary gas mixture conditions. The outstanding performances of the sensors are attributed to the altered dispersion states of surface NO_2_ molecules and the enhanced electron transfer between supports and NO_2_, demonstrating by Ab Initio Molecular Dynamics (AIMD) and in-situ Raman results. Compared to commercial sensors, the 1%-PtSn-SnO_2_ sensors can operate continuously for more than 550 days in air, outperforming state-of-the-art systems. Furthermore, by integrating characteristic-specific sensors (including PtSn-SnO_2_, SnO_2_, Pt_1_-SnO_2_, and Pt NPs-SnO_2_) into an array, machine-learning analysis shows 100% classification accuracy for both NO_2_ and mixed gases (for a library of 37 gases). At the same time, we also equip these sensor arrays onto humanoid robots to build a cruise-monitoring system, in which the olfactory module accurately recognizes NO_2_ in complex gas mixtures. Overall, this bio-inspired atomic internalization strategy offers a general and scalable route for constructing a robust gas-sensing system capable of reliable operation in complex environments.

## Results

### Internalization process of PtSn-Sn NFs

**Fig. 2:**
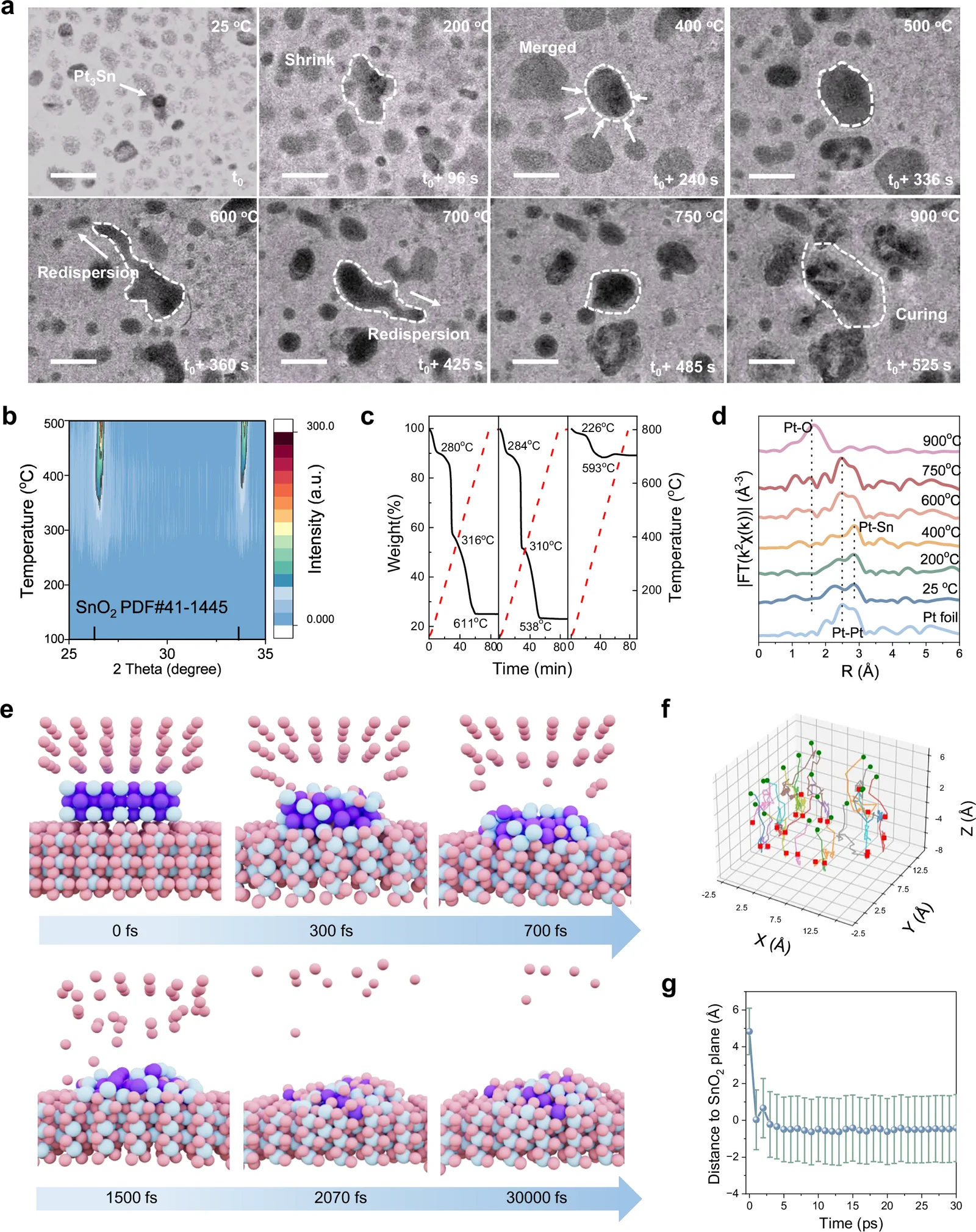
Evolution of Pt_3_Sn alloys during pyrolysis. **a** In-situ TEM images at various temperatures of 1%-PtSn-Sn NFs in air (the scale bars are 50 nm). **b** XRD patterns of 1%-PtSn-Sn NFs at different temperatures. **c** TGA patterns of 1%-PtSn-Sn NFs (left), Sn NFs (middle) and Pt_3_Sn alloys (right). **d** K^2^-weighted Pt L_3_-edge EXAFS spectra of Pt foil and 1%-PtSn-Sn NFs treated at different temperatures. **e** MD simulations of the Pt_3_Sn alloy during the annealing process (pink, blue, and purple balls represent O, Sn, Pt atoms, respectively). **f** The trajectories of randomly selected 20 Pt atoms extracted from MD simulations during 0-100 ps. Initial green dots gradually move into the terminal red cube, suggesting the downward migration of Pt species. **g** Average distances between Pt atoms and the SnO_2_ plane from an MD trajectory. Error bars indicate the standard deviation across 51 Pt atoms within each simulation frame. Source data are provided as a Source data file.

Initially, Pt_3_Sn alloy nanoparticles (NPs) with a uniform diameter of approximately 20 nm were fabricated via a surfactant-assisted method (Fig. S1). By mixing Pt_3_Sn alloy, Sn sources, and polymeric polyvinylpyrrolidone (PVP), a series of nanofibers (NFs) precursors were successfully synthesized via electrospinning technology (denoted as Sn NFs, 0.3%-PtSn-Sn NFs, 0.6%-PtSn-Sn NFs, 1.0%-PtSn-Sn NFs, 1.5%-PtSn-Sn NFs and 2.0%-PtSn-Sn NFs, respectively). The Pt contents of these samples are shown in Table S1. As seen in Figs. S2, 3, the fibers exhibit average diameters of approximately 200 nm, and noticeable agglomeration is observed when the alloy loading exceeds 1.5 wt%. Scanning electron microscopy (SEM) shows that pyrolysis of Sn NFs is accompanied by oxidation and volatilization of the polymer precursors to form SnO_2_ (Fig. S4). When pyrolyzing the 1.0%-PtSn-Sn NFs in air, detailed structural evolution was recorded by in-situ transmission electron microscopy (TEM) (Fig. 2a and Movie S1). After a simple preprocessing step, the Pt_3_Sn alloys first adhere to the Sn NFs. As the temperature gradually rises to 500 °C, the surrounding Sn precursors exhibited a “shrink and merge” behavior to form crystalline SnO_2_ supports, leading to wrapped and restricted Pt_3_Sn derivatives within the SnO_2_ interior. Upon further heating to 500 °C–750 °C, the fused body gradually disintegrates and re-disperses, and the product finally stabilizes at 900 °C with no obvious Pt_3_Sn alloy remaining on the SnO_2_ surface. The time-resolved images of the entire pyrolysis process are displayed in Fig. S5. High-resolution transmission electron microscopy (HRTEM) and energy-dispersive X-ray spectroscopy (EDS) mapping provide additional evidence for the absence of Pt_3_Sn NPs and Pt NPs on the SnO_2_ surface (Fig. S6). Phase evolution analysis by X-Ray diffraction (XRD) for 1%-PtSn-Sn NFs exhibits characteristic peaks of rutile SnO_2_ at 26.5 ° and 33.7 ° once the pyrolysis temperature reaches 300 °C (Fig. 2b), and the crystal structure gradually stabilizes above 500 °C (Fig. S7). This result is consistent with the in-situ TEM observations, showing agglomeration and fusion of Sn/C precursors to form crystalline SnO_2_ at 500 °C. Moreover, pyrolysis of 1%-PtSn-Sn NFs, Sn NFs, and the pristine Pt_3_Sn alloy was monitored by thermogravimetric analysis (TGA) (Fig. 2c and Fig. S8). The weights of all three samples stabilize at approximately 500 °C–600 °C, corresponding to carbon volatilization and SnO_2_ formation. For Pt_3_Sn alloys, a slight increase in weight at 590 °C–610 °C is attributed to Sn oxidation to form SnO_2_, marking the onset of dissolution and internalization of Sn species in Pt_3_Sn.

To investigate the evolution of Pt species during pyrolysis, ex situ X-ray absorption fine structure (XAFS) analyses were performed on 1%-PtSn-Sn NFs after annealing at 200 °C–900 °C. As seen in Figure. S9, the Pt L_3_-edge X-ray absorption near-edge structure (XANES) spectra reveal continuous oxidation of Pt species during pyrolysis. In addition, the extended X-ray absorption fine structure (EXAFS) spectra (Fig. 2d) display a dominant peak at 2.8 Å (Pt-Sn) when the pyrolysis temperature is below 600 °C, indicating that Pt mainly exists in the form of Pt_3_Sn alloys^33^. At 600 °C–750 °C, the main Pt coordination peak shifts to 2.5 Å (Pt-Pt), implying that the Pt configuration transforms from Pt_3_Sn alloys to Pt NPs, consistent with the TGA results. At 900 °C, only a single peak at 1.6 Å (Pt-O) is observed, indicating monodispersion of Pt species on the support surface. These EXAFS results demonstrate that, through a two-step internalization process, the Pt_3_Sn alloys are gradually converted first into Pt NPs and then into Pt single atoms during pyrolysis (schematic illustration shown in Fig. S10). Moreover, the annealing process is further analyzed by molecular dynamics (MD) simulations (Fig. 2e). Initially, the permeation of oxygen and Sn drives the disintegration of the Pt_3_Sn alloy, leading to a disordered amorphous structure. Subsequently, oxygen species assist Pt migration toward the support surface and ultimately yield dispersed Pt single atoms. The trajectories of representative Pt atoms (Fig. 2f) and the average distances between Pt atoms and the SnO_2_ plane (Fig. 2g and Fig. S11) clearly illustrate downward migration and redispersion of Pt species. Furthermore, Pt_3_Sn alloys with different loadings (including 0.3%-PtSn-SnO_2_, 0.6%-PtSn-SnO_2_, 1.0%-PtSn-SnO_2_, 1.5%-PtSn-SnO_2_, 2.0%-PtSn-SnO_2_) exhibit distinct agglomeration of Pt species into nanoparticles (30–60 nm) when the loading exceeds 1.5% (Fig. S12–15).

### Segregation behaviors of monodispersed Pt species

**Fig. 3:**
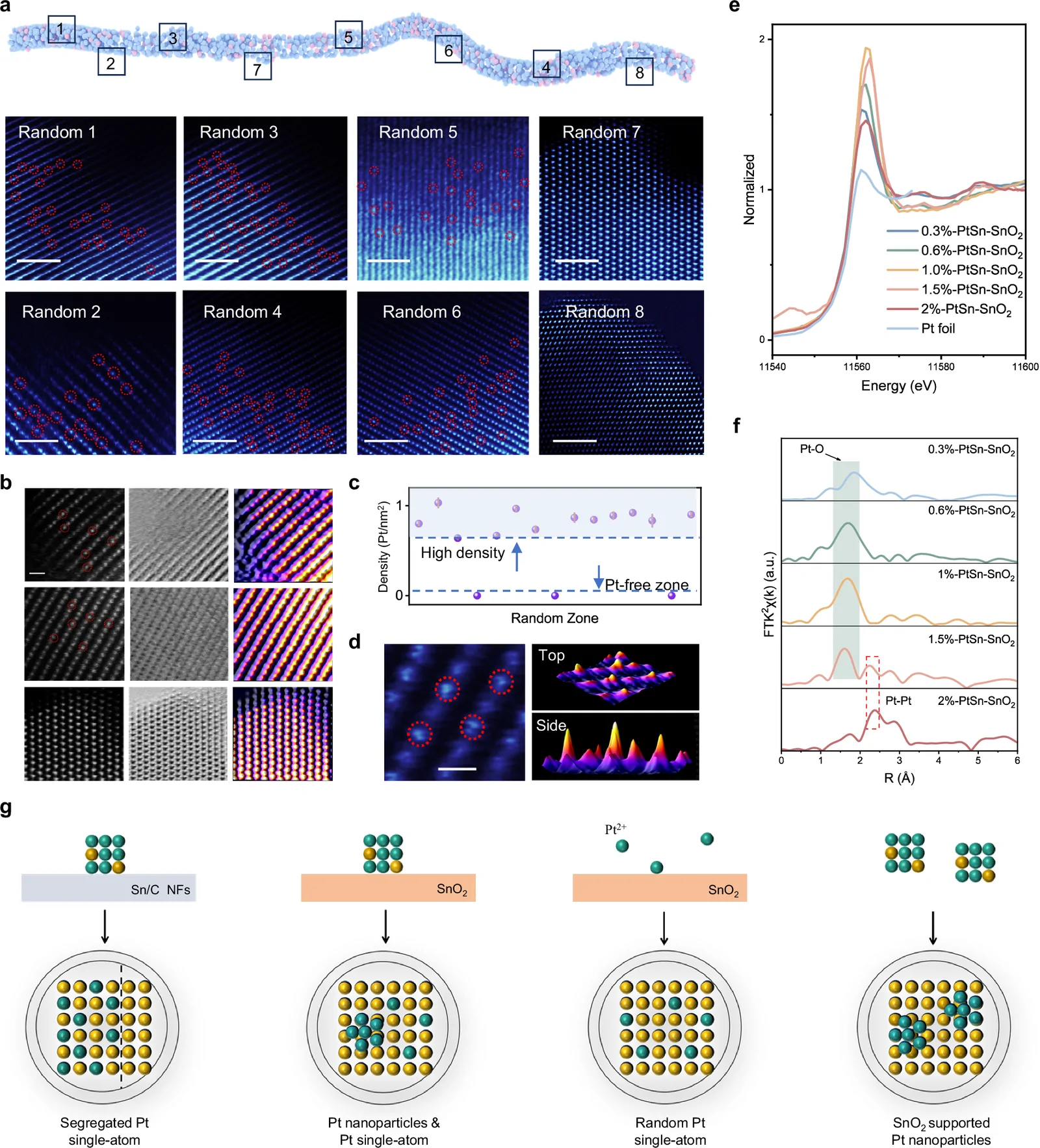
Evolution and distribution mechanism of Pt species. **a** HAADF-STEM images of eight random regions in 1%-PtSn-SnO_2_ samples (the scale bars are 2 nm). **b** Additional HAADF-STEM images of 1%-PtSn-SnO_2_ samples (the scale bars are 0.5 nm).**c** The calculated Pt density distribution from 15 random regions in 1%-PtSn-SnO_2_ samples. **d** HAADF-STEM images and three-dimensional atom overlapping Gaussian-function fitting mapping of 1%-PtSn-SnO_2_ samples. **e** Pt L-edge XANES spectra. **f** FT-EXAFS spectra of Pt L_3_-edge. **g** Schematic of the different evolution paths of Pt species during the pyrolysis process. Source data are provided as a Source data file.

High-angle annular dark-field scanning transmission electron microscopy (HAADF-STEM) was implemented to investigate the distribution pattern of Pt species on SnO_2_. As seen in Fig. 3a, b and Fig. S16, eight randomly selected regions from single nanorods of 1%-PtSn-SnO_2_ exhibit markedly different Pt concentrations across the SnO_2_ surface. Notably, isolated Pt atoms are clearly observed (marked in red circles) in regions 1–6, while regions 7-8 display negligible Pt signals. Specifically, regions 1–6 show segregated yet highly dense Pt distributions (~0.9/nm^2^) in localized areas, in sharp contrast to the Pt-free regions 7-8. To corroborate these uniquely dispersed modes, additional HAADF-STEM images were collected (Fig. S17). The Pt atom densities from 15 selected areas were quantified (Fig. 3c), revealing a bipolar distribution (either 0 or about 0.9 ± 0.05 atoms/nm^2^). The intensity profiles and three-dimensional atom overlapping Gaussian-function fitting mapping further confirm the atomic-level dispersion of individual Pt atoms on the SnO_2_ supports (Fig. 3d and Fig. S18).

The evolution of Pt species in various concentrations during pyrolysis was further investigated. As shown in Fig. 3e, the white-line intensities of all PtSn-SnO_2_ samples are higher than those of Pt foil, indicating higher oxidation states of Pt. The FT-EXAFS spectra of the Pt L_3_-edge reveal only Pt-O bonds in the 0.3%-PtSn-SnO_2_, 0.6%-PtSn-SnO_2_, and 1%-PtSn-SnO_2_ samples (Fig. 3f), while both Pt-Pt and Pt-O bonds exist in the 1.5%-PtSn-SnO_2_, 2%-PtSn-SnO_2_ samples, indicating that excessive Pt_3_Sn alloy promotes the formation of Pt NPs^33^. (The FT-EXAFS spectrum is not corrected for phase shift, therefore, the peak positions are shorter than the actual atomic distances.) This is conformed to the mapping results in Fig. S15. Furthermore, the detailed structural evolution of Pt NPs in 1.5%-PtSn-SnO_2_ was recorded by in-situ TEM (Fig. S19) and EDS-mapping (Fig. S20), both showing that adjacent Pt_3_Sn alloys coalesce within confined space to form Pt NPs. To probe the effect of different supports and impregnation methods on the dispersion patterns of Pt species, comparative experiments were conducted (Fig. 3g). When directly annealing outer-1%-Pt_3_Sn-SnO_2_ (impregnating Pt_3_Sn into SnO_2_ NFs) in air at 900 °C, four random HAADF-STEM areas of the resulting outer-1%-PtSn-SnO_2_ samples reveal the coexistence of both monodispersed Pt species and aggregated Pt NPs (Figure. S21). This suggests that Sn/C NF precursors play a crucial “receptor” role in engulfing Pt species and restricting their directionless volatilization, thereby favoring the formation of aggregation-dispersed single-atom catalysts. In contrast, the conventional wet impregnation method yields only randomly dispersed Pt single atoms (Fig. S22), whereas SnO_2_ supported Pt NPs can be found via directly annealing Pt_3_Sn alloy in air at 900 °C (Fig. S23). Collectively, these microscopic and spectroscopic results demonstrate that the Sn/C NF-mediated internalization route uniquely directs Pt into locally segregated, high-density single atoms on SnO_2_ while preventing undesired NPs aggregation.

### Gas-sensing performances analysis

**Fig. 4:**
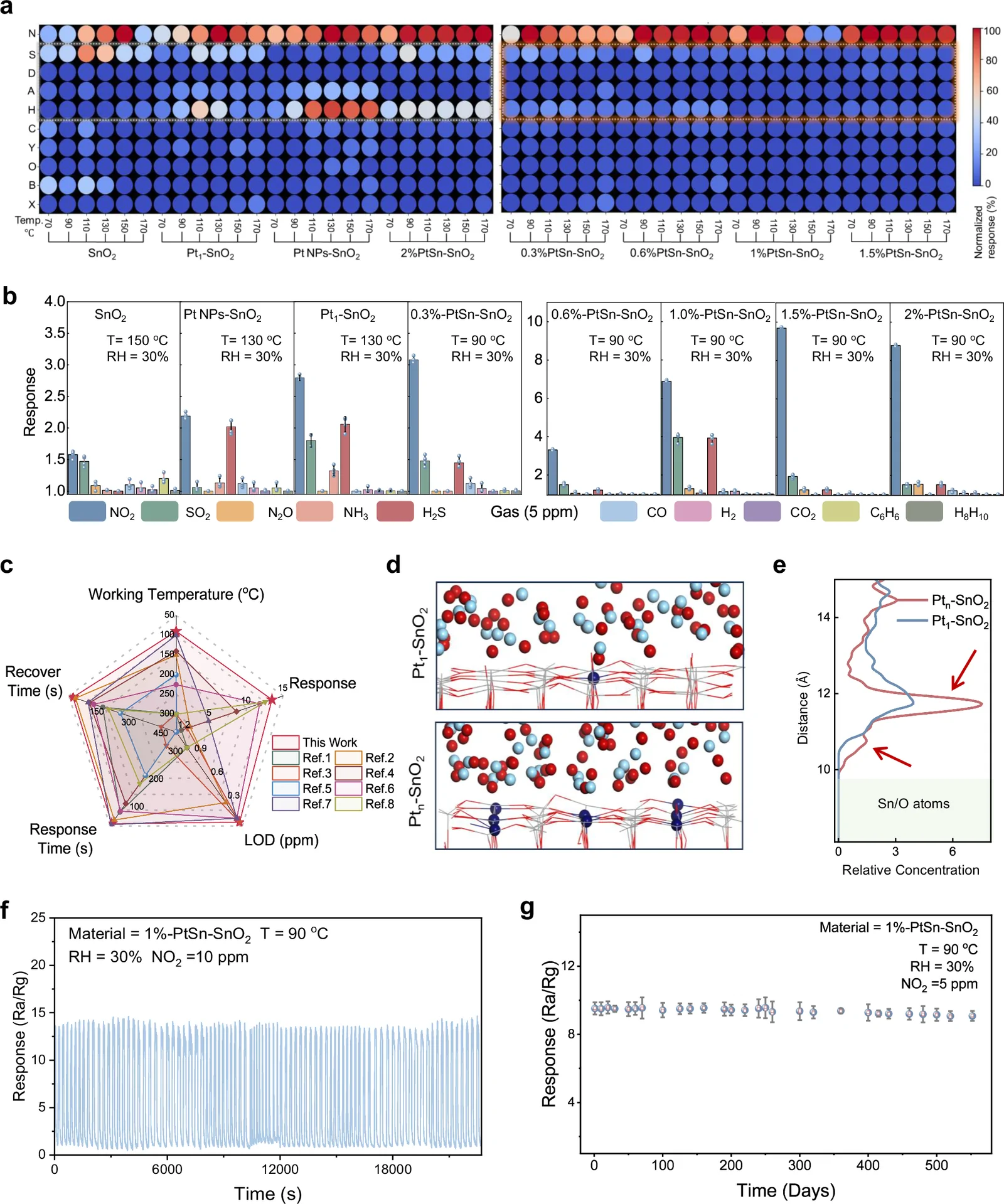
Gas-sensing performances and mechanistic studies. **a** Normalized signals of SnO_2_, Pt NPs-SnO_2_, Pt_1_-SnO_2_, 0.3%-PtSn-SnO_2_, 0.6%-PtSn-SnO_2_, 1%-PtSn-SnO_2_, 1.5%-PtSn-SnO_2_ and 2%-PtSn-SnO_2_ to ten different gases under different temperatures. **b** The response values of the eight sensors to the eight different gases under optimal working temperature. All results represent the average values from four different sensors, and the error bars indicated the standard deviation. All results represent the average values from different sensors (*n*  =  4). **c** Comparative studies of NO_2_ sensors. **d** Representative snapshots of the interfacial structures with NO_2_ molecules on Pt_1_-SnO_2_ (top) and Pt_n_-SnO_2_ (down) surfaces. Red, blue, mazarine balls represent O, N, and Pt atoms, respectively. **e** Concentration distribution profiles of NO_2_ molecules along the surface normal direction. **f** Stability tests of 1%-PtSn-SnO_2_ sensors to 10 ppm NO_2_. **g** Long-term stability tests of 1%-PtSn-SnO_2_ sensors to 5 ppm NO_2_ over 550 days. All results represent the average values from three sensors, and the error bars indicated the ten times the standard deviation. All results represent the average values from different sensors (*n*  =  4). Source data are provided as a Source data file.

To explore the gas-sensing capabilities of PtSn-SnO_2_ materials (0.3%-PtSn-SnO_2_, 0.6%-PtSn-SnO_2_, 1%-PtSn-SnO_2_, 1.5%-PtSn-SnO_2_, 2%-PtSn-SnO_2_), the PtSn-SnO_2_ samples together with pristine SnO_2_, Pt_1_-SnO_2_, and Pt NPs-decorated SnO_2_ (Pt NPs-SnO_2_) were tested as contrasted analysis. (Figs. S24–28; more details are provided in the Methods section). As seen in Fig. 4a, the sensensing performances the eight sensors toward ten gases (including N (NO_2_), S (SO_2_), D (N_2_O), A (NH_3_), H (H_2_S), C (CO), Y (H_2_), O (CO_2_), B (C_6_H_6_) and X (C_7_H_8_)) at different temperatures were evaluated. All PtSn-SnO_2_-based sensors demonstrate favorable responses to NO_2_, with 0.3%-PtSn-SnO_2_, 0.6%-PtSn-SnO_2_, 1%-PtSn-SnO_2_, and 1.5%-PtSn-SnO_2_ exhibiting markedly higher selectivity and sensitivity than SnO_2_, Pt_1_-SnO_2_, Pt NPs-SnO_2_, and 2%-PtSn-SnO_2_. Among the eight sensors, 1%-PtSn-SnO_2_ displays the highest response values (5.81/3 ppm) and the lowest detection limit (0.05 ppm) for NO_2_ (Fig. S29–32 and Table 2–5). In addition, its relatively low operating temperature (90 °C) can minimize the power consumption (6 mW) in micro-electromechanical system (MEMS) devices (Fig. S30). The response performances of the eight sensors toward the ten gases at their respective optimal working temperature are summarized in Fig. 4b. The Pt NPs-SnO_2_ and Pt_1_-SnO_2_ sensors show enhanced responses to multiple gases (such as NO_2_ and H_2_S) compared to pristine SnO_2_. Notably, the NO_2_ sensitivity of the PtSn-SnO_2_ sensors increases with Pt content and reaches a maximum at 1% Pt_3_Sn loading, whereas SO_2_ and H_2_S sensitivities are highest at 2% Pt_3_Sn loading, which may be attributed to the presence of Pt NPs in 2%-PtSn-SnO_2_. More detailed gas-sensing analyses of 1%-PtSn-SnO_2_ sensor show rapid response and recovery times (18.6 s and 39.4 s to 10 ppm NO_2_), stable responses under varying humidity levels (responses reduce only 7.1% at 90% RH environment), high adsorption and desorption rate constants (0.145 and 0.15 s^-1^), and relatively low activation energies (3.86 J/mol) (Figs. S33–39). Together, these parameters establish the 1%-PtSn-SnO_2_ sensor supas erior to many previously reported NO_2_ sensors (Fig. 4c and Table S6).

To gain mechanistic insights into the superior NO_2_ sensing performance of 1%-PtSn-SnO_2_, systematic Ab Initio Molecular Dynamics (AIMD) simulations were performed on SnO_2_ surfaces modified with single and multiple Pt atoms (Pt_1_-SnO_2_ and Pt_n_-SnO_2_; see Theoretical Calculation and Fig. S40 for more computational details). As shown in Fig. 4d, the presence of individual Pt atoms promotes the adsorption of interfacial NO_2_ molecules (Fig. S41). Higher Pt loadings further alter the distribution pattern of NO_2_, leading to the formation of a hierarchical structure that interacts more strongly with the Pt-modified SnO_2_ surface (Fig. S42). This phenomenon is confirmed by the concentration distribution profiles of NO_2_ along the surface normal direction, which show that Pt_n_-SnO_2_ surfaces accumulate more NO_2_ molecules than Pt_1_-SnO_2_. (Fig. 4e).

In addition, NO_2_ kinetic adsorption was evaluated by quartz crystal microbalance (QCM) measurements. As shown in Figs. S43, 44, the 1%-PtSn-SnO_2_ sample exhibits a significantly higher adsorbed mass than both the Pt_1_-SnO_2_ and pristine SnO_2_, confirming its stronger NO_2_ uptake. Moreover, in-situ Raman spectra of 1%-PtSn-SnO_2_ and SnO_2_ samples were collected over 120 s during exposure to 10 ppm NO_2_ (Figs. S45, 46). The peak located at 621 cm^-1^(*A*_1g_ vibrations of 1%-PtSn-SnO_2_ sample) gradually shifts to lower wavenumbers after NO_2_ exposure^34^, while no obvious changes for SnO_2_ sample. These variations are mainly because the broken symmetrical stretching vibrations of the SnO_2_ lattice after adsorbing and interacting with NO_2_ molecules. In addition, the repeatability of the 1%-PtSn-SnO_2_ sensor toward 10 ppm NO_2_ gas detection at 90 °C and 30% RH was examined. As seen in Fig. 4f, the 1%-PtSn-SnO_2_ sensor demonstrates exceptional stability over 100 cycles, with a response range of 13.5 ± 0.5. Long-term stability tests reveal only a 4.6% decline over 550 days to 5 ppm NO_2_ (Fig. 4g), emphasizing its strong potential for practical application.

### Assessments of sensor arrays

**Fig. 5:**
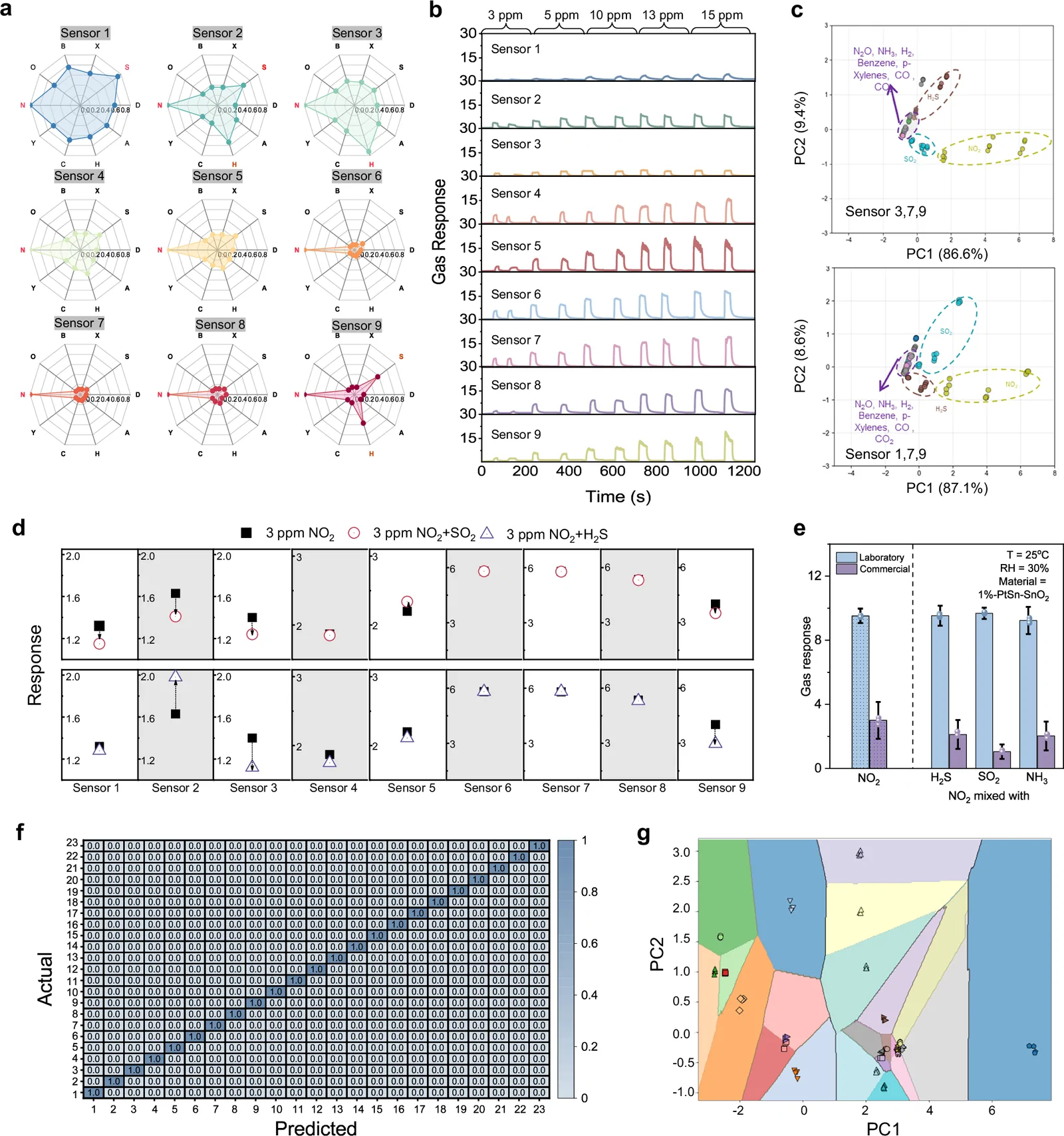
Gas detection in multi-gas atmospheres. **a** Selectivity of individual sensors in the sensor array for 5 ppm different gases **b** Dynamic sensing curves of the nine sensors in the sensor array for NO_2_ at 3–15 ppm. **c** PCA plots obtained from given groups of sensors (sensor 1, 3, 7 and sensor 1, 7, 9). **d** Changes in response values of nine sensors under three different gas environments. **e** Performances of the commercial and 1%-PtSn-SnO_2_ sensors in different gas environments. All results represent the average values from two different sensors, and the error bars indicated ten times the standard deviation. All results represent the average values from different sensors (*n*  =  4). **f** Confusion matrix on classifying 23 types of gases. **g** KNN classification boundaries for 23 types of gases. The legend is shown in Fig. S55. Source data are provided as a Source data file.

Based on the detection performances of the SnO_2_-based sensors toward different gases, a 3×3 multi-sensor array was constructed containing (1) SnO_2_, (2) Pt_1_-SnO_2_, (3) Pt NPs-SnO_2_, (4) 0.3%-PtSn-SnO_2_, (5) 0.6%-PtSn-SnO_2_, (6) 1%-PtSn-SnO_2_, (7, same as 6) 1%-PtSn-SnO_2_, (8) 1.5%-PtSn-SnO_2_, (9) 2%-PtSn-SnO_2_, with each component labeled 1-9, respectively (Fig. S47). The selectivity coefficients (S=R_gas_/R_NO2_) were introduced to describe their relative responses toward the ten different gases. As seen in Fig. 5a, sensors 4-8 show prominent NO_2_ capacity, while sensors 1-3 and 9 display different selectivity patterns for multi-gas. Furthermore, dynamic sensing curves for five NO_2_ concentrations (3-15 ppm) show stable and reliable responses of this array system (Fig. 5b). Considering the different selectivity of each sensor, the principal component analysis (PCA) method was performed to elucidate the discriminative ability of gases through combinatorial sensors. As shown in Fig. 5c, groups that contain at least one of the sensors 4–7 (such as groups 1, 5, 9; 1, 4, 9; 1, 2, 4; 1, 2, 5; and 1, 3, 4) effectively distinguish between NO_2_, H_2_S and SO_2_, whereas groups that do not contain any of the sensors 4-7 (such as groups 1, 3, 9; 1, 2, 3; and 1, 8,9) exhibit poor discriminative ability for these gases (Fige. S48).

In addition, the sensing actions of the sensor array under multi-gas conditions were tested to elucidate its ability to distinguish different gases in complex environments. As shown in Fig. S49, two or three different gases were simultaneously injected into a test volume by a static testing method. Comparing 3 ppm NO_2_ + 3 ppm SO_2_ and 3 ppm NO_2_ + 3 ppm H_2_S atmospheres with those in pure NO_2_, the response values of sensors 1, 2, 3 decrease by 12.8%, 11.4%, 13.5%, and those of sensors 2, 3, 4 decline by 20%, 21.5%, 6.4%, respectively, whereas sensors 5-8 maintain nearly unchanged responses under both conditions (Fig. 5d). Additional cross-mixed concentrations and gas atmospheres further demonstrate the outstanding interference resistance and stability of sensors 6 and 7 (Fig. S50–53). When the detection conditions are switched to ternary gas mixtures with various concentrations and compositions (Fig. S54), sensors 6 and 7 still exhibit superior performance compared with the other sensors. Furthermore, a world-class commercial NO_2_ sensor (GM-102B) was tested and compared with the as-prepared 1%-PtSn-SnO_2_ sensor in multi-component atmospheres. As shown in Fig. 5e, the 1%-PtSn-SnO_2_ sensor delivers more than triple the response signal intensity of the GM-102B sensor toward 5 ppm NO_2_. In addition, the response fluctuations of the GM-102B sensor are about 30, 65, 32.3% upon the introduction of H_2_S, SO_2_ and NH_3_ in equal concentrations, respectively, while the responses of 1%-PtSn-SnO_2_ sensors remain nearly unchanged. Therefore, the 1%-PtSn-SnO_2_ sensor not only exhibits excellent selectivity in single-gas environments but also demonstrates superior interference-resistant properties under multi-gas conditions. These fundamental sensing properties are crucial for integrating multiple sensors and thus achieving reliable detection of both single-component and complex gas mixtures. As summarized in Table S7, a set of 37 gas atmospheres, including both single and multi-component gases, was constructed. The confusion matrix illustrates a clear dominance of diagonal elements, signifying a 100% gas classification accuracy (Fig. S56). Additionally, the classification boundaries display clear differences for all 37 target gases (Fig. S57), and representative 23 gases (Table S8) are further exhibited in Fig. 5f, g. Moreover, K-NearestNeighbor (KNN) classification boundaries for single-component, two-component, and multi-component gases were generated and displayed in Fig. S58. Notably, the number of sensors in the array can be effectively reduced to three while still maintaining 100% gas classification accuracy (Fig. S59). Together, these sensor array experiments and classification analyses demonstrate that sensors containing locally enriched Pt single atoms can deliver robust, interference-resistant, and highly accurate recognition of various single and mixed gases in complex environments.

### Cruise-monitoring system on a humanoid robot with smart sensors

**Fig. 6:**
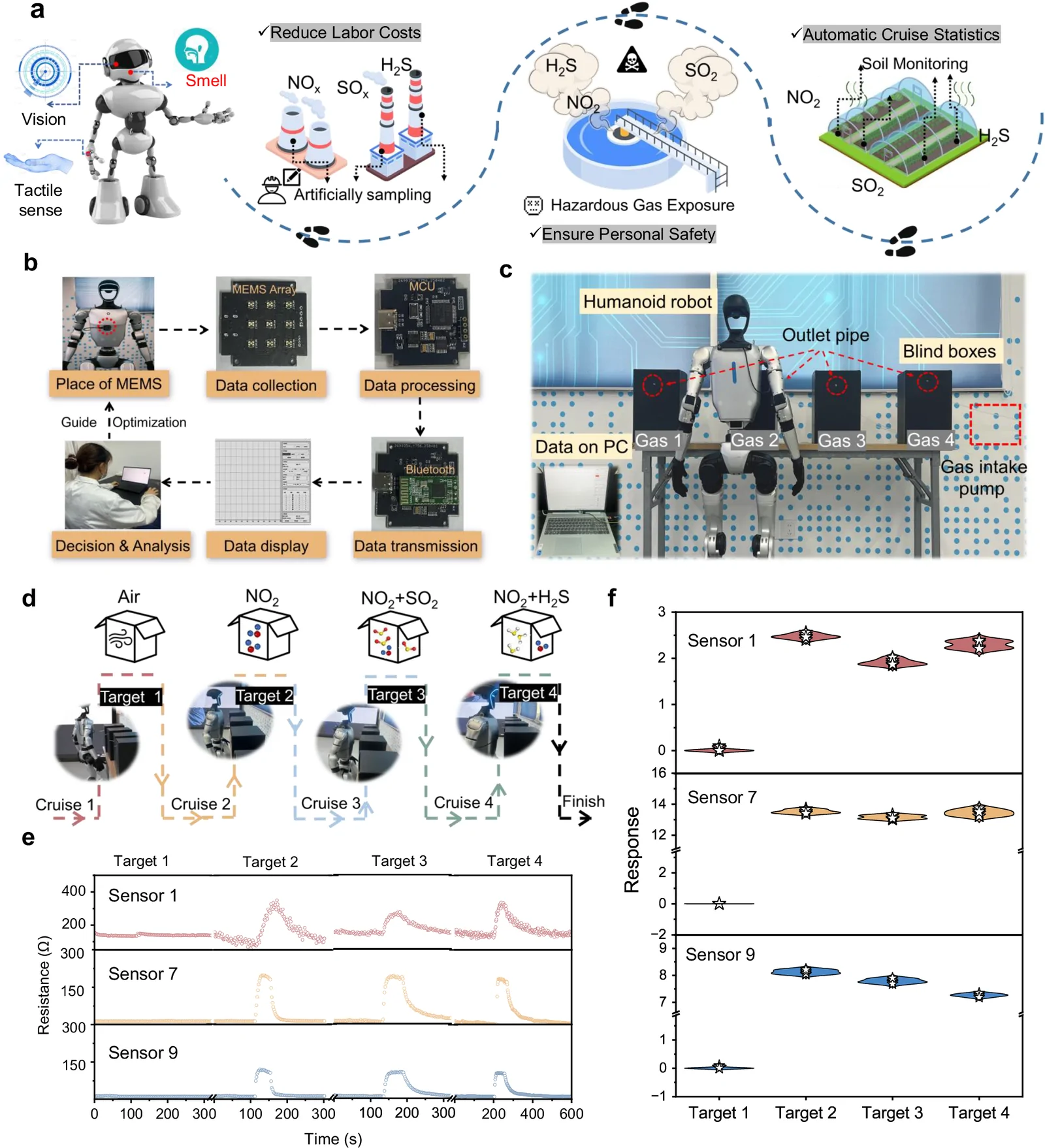
Integration of an olfactory platform on a humanoid robot for a cruise-monitoring system. **a** Illustrations of humanoid robots equipped with intelligent olfactory platforms in different scenarios. **b** Detailed functioning procedure of the olfactory platform. **c** Configuration of the intelligent olfactory platform on a humanoid robot for the blind-box experiments. **d** Route map during the cruising experiments. **e** Dynamic sensing curves for three sensors during the blind-box experiments. **f** Statistical analysis of the response values obtained from repeated experiments. All results represent the average values from three different sensors. All results represent the average values from different sensors (*n*  =  6). Source data are provided as a Source data file.

Given typical scenes in chemical plants and sewage treatment facilities, NO_2_ is a common gas pollutant whose emission is often accompanied by other gaseous components, such as SO_2_ and H_2_S^35–38^. Accurate monitoring of NO_2_ concentrations is crucial for optimizing production processes, preserving equipment, and maintaining operational safety^39,40^. In addition, measuring NO_2_ emissions from soil in greenhouses is a way to assess nitrogen fertilizer utilization by plants, and similar approaches can be applied for the detection of SO_2_ and H_2_S originating from sulfur fertilizers^41,42^. Considering the excellent sensing performance of the sensor array, a humanoid robot equipped with these smart sensors can autonomously cruise and monitor gases in real time, thereby reducing labor costs, minimizing exposure to hazardous gases, and enabling intelligent sampling (Fig. 6a). As shown in Figs. S60–62, the nine sensors were integrated into an olfactory platform, comprising modules of energy management, data collection and processing, Bluetooth transmission, and other functions. Afterwards, the olfactory platform was assembled and mounted on a humanoid robot equipped with visual sensors for practical cruise and real-time gas monitoring (Fig. 6b). In the configured scenarios, four blind boxes with the same appearance (indistinguishable to the visual sensors on the robot) were placed in a row and connected to air (Target 1), a single-tube NO_2_ (Target 2), a double-tube NO_2_ + SO_2_ (Target 3), and another double-tube NO_2_ + H_2_S (Target 4), respectively (Fig. 6c; more details are provided in Notes S3). In the testing process, a patrol route was predesigned to ensure the sequential identification of each box (Fig. 6d). During navigation along the specified route, the robot approached each target box, and the sensor array in the olfactory platform was used to identify the gas species and composition inside each box. The real-time signals from representative sensors 1, 7, and 9 in the sensor array, along with the statistical results from multiple patrol cycles, are presented in Fig. 6e, f. No obvious resistance changes for any of the three sensors around Target 1, indicating ambient air inside the box. The response value of sensor 1 shows the greatest variation near Target 3, whereas sensor 9 responds most strongly to Target 4, while the response values of sensor 7 remain nearly constant around Targets 2–4, demonstrating its high anti-interference properties.

## Discussion

In summary, we propose a bio-inspired approach to internalize Pt_3_Sn alloys as thermostable Pt single atoms on regionalized SnO_2_ surfaces. The segregated yet highly dense dispersion mode of Pt single atoms effectively alters the adsorption mode of NO_2_ molecules and exhibits exceptional NO_2_ sensing selectivity, stability, and interference-resistant capability. Even in multi-component atmospheres, the system achieves accurate and stable NO_2_ monitoring over 550 days, surpassing state-of-the-art commercial sensors. By integrating individual characteristic-specific sensors into an array and combining it with machine learning algorithms, 100% identification accuracy of target gases is achieved at low cost. Finally, a cruise-monitoring system constructed by equipping the sensor array on a humanoid robot demonstrates a complete, practical prototype for intelligent gas detection. Overall, this work paves the way toward robust, autonomous monitoring of complex industrial and environmental gas emissions.

## Methods

### Synthesis of Pt_3_Sn alloys

In a typical synthetic procedure, platinum (II) acetylacetonate (Pt(acac)_2_), 9.8 mg), tin (II) chloride dihydrate (SnCl_2_·2H_2_O, 1.904 mg), and polyvinylpyrrolidone (PVP, M.W. 30,000, 80 mg) were weighed into a 10 mL beaker. N,N-Dimethylformamide (DMF) (5 mL) was added to the beaker containing the starting materials. The mixture was stirred for 30 min, resulting in a pale-yellow clear solution. This solution was then transferred into a Teflon-lined autoclave. The reaction was maintained at 180 °C for 12 h. After cooling to room temperature, the product was collected by centrifugation with acetone (three times).

### Synthesis of PtSn-Sn NFs

The as-synthesized Pt_3_Sn alloy (8 mg), SnCl_2_·2H_2_O (1.2 g), and PVP (M.W. 1,000,000, 1.6 g) were weighed into a beaker containing a mixed solvent of DMF (9 mL) and ethanol (2 mL). The mixture was stirred at room temperature for 48 h until a homogeneous and viscous solution was obtained, yielding the electrospinning precursor. Subsequently, electrospinning was performed by applying a voltage of 16 kV between the nozzle and the collector, with a fixed distance of 15 cm. During the process, the resulting 1%-PtSn-Sn NFs were collected on an aluminum foil-covered collector. The loading of the alloy was initially calculated based on the mass ratio of Pt in the Pt_3_Sn alloy to Sn in the tin precursor. The samples of 0.3%-PtSn-Sn NFs, 0.6%-PtSn-Sn NFs, 1.5%-PtSn-Sn NFs, and 2.0%-PtSn-Sn NFs were obtained by adding 2.5 mg, 5 mg, 12 mg, and 16 mg of Pt_3_Sn alloy precursor, respectively.

### Pretreatment of PtSn-SnO_2_

The as-synthesized PtSn-Sn NFs were first stabilized at 200 °C for 1 hour. Subsequently, the sample was transferred to a ceramic boat and heated in a tube furnace at 900 °C for 2 h in air, with a programmed heating rate of 5 °C/min, to obtain the final PtSn-SnO_2_ product.

### Statistics & reproducibility

No statistical method was used to predetermine sample size. No data were excluded from the analyses. The experiments were not randomized. The investigators were not blinded to allocation during experiments and outcome assessment. Statistical analyses were performed using Origin 2026. All experiments were repeated at least three times independently with similar results.

### Characterization of materials

X-ray diffraction patterns (XRD) were collected on a powder XRD diffractometer (Rigaku D/MAX-2500 X-ray, with Cu-Kα_1_ radiation λ = 0.154056 nm 40 kV and 40 mA, scanning from 10° to 70°). Samples of 5.00 ± 0.05 mg were placed in 150 μL aluminum oxide crucibles for thermogravimetric analysis (TG, HITACHI STA200/HITACHI STA300). The high-resolution TEM (HRTEM, JEM-2100F, Japan) and high-angle annular dark-field scanning TEM (HAADF-STEM) images were recorded on an FEI Tecnai G2 F20 S-Twin high-resolution transmission electron microscope (Hillsboro, OR, United States) set at 200 kV, and a JEOL JEM-ARM300F TEM/STEM (Tokyo, Japan), respectively, with a spherical aberration corrector worked at 300 kV. Through-focal HAADF series were acquired at nanometer intervals, with the first image under-focused (beyond the beam exit surface) and the final image over-focused (before the beam entrance surface). Then the images were aligned manually to remove the sample drift effects.

### Gas-sensing measurement

The gas-sensing tests were performed on an intelligent gas-sensing analysis system of the high-precision multi-channel sensor testing system EVM-HP-MC02 (Shanghai Lingpan Electronic Technology Co., Ltd., China). The gas-sensing system is shown in Figs. S27, 28. The operating voltage of the test circuit is 5 V. The operational resistance of the sensor was calculated from the output working voltage of the testing system, and the concentration of the test gas was determined using a static test method. The target gases were diluted using air as the balance gas and then injected into the test chamber. All gas-sensing tests were conducted in an environmentally controlled chamber capable of maintaining constant temperature and humidity, with the test temperature stabilized at 28 °C. More specific details about gas-sensing measurements were shown in Supplementary Information.

### Theoretical calculation

The specific software, modules and functions used for AIMD and MD are detailed in Supplementary Information. During ab initio molecular dynamics (AIMD) simulations, a 1 × 1 × 1 Γ-centered k-point mesh was employed. The top atomic layer was allowed to relax, while the remaining layers were fully fixed. AIMD simulations were performed in the canonical (NVT) ensemble at 363 K using a Nosé-Hoover thermostat, with a total simulation time of 12 ps and a time step of 1.0 fs. The concentration profiles were obtained by averaging over 4000 configurations collected from the last 8-12 ps of the AIMD trajectory. The initial structures for AIMD simulations were taken from the DFT-optimized ones.

For MD simulations, oxygen atoms were placed above the solid surfaces to model the gas–solid interface. In total, 657 atoms were included in the simulation box with 1.92 × 3.35 × 3.50 nm, composed of 51 Pt atoms, 160 Sn atoms, and 392 oxygen atoms. Among them, 54 oxygen atoms were treated as gas-phase O atoms positioned above the SnPt surface^43,44^. The force field parameters between all atoms were modeled by a Lan-type potential, obtained from openkim.org^45,46^.

### Reporting summary

Further information on research design is available in the Nature Portfolio Reporting Summary linked to this article.

## Supplementary information

Supplementary Information

Description of Additional Supplementary Files

Supplementary Movie 1

Reporting Summary

Transparent Peer Review file

## Source data

Source Data

## Data Availability

The data supporting the findings of this study are reported in the main text or the Supplementary Information. Source data are provided with this paper.

## Code availability

The custom code used for the machine learning analysis is available from the Zenodo open repository at (https://zenodo.org/records/21626430).
