# Supplementary material for: Creating locally enriched single-atom catalysts via bio-inspired internalization process for interference-resistant NO2 detection in complex environments
Source: Nat Commun. 2026 Aug 20;17:9949. doi: 10.1038/s41467-026-76850-4 (PMC13586179; doi:10.1038/s41467-026-76850-4)
Supplement: Supplementary file 1 [file 41467_2026_76850_MOESM1_ESM.pdf]

Supplementary Information

Creating locally enriched single atom catalysts via bio-inspired internalization process for interference-resistant NO_2 detection in complex environments

Xin Jia[†1,2], Runfang Mao[†1], Shan Hu[†3], Muyu Yan[4], Haotian Huang[1], Zhiheng Ma[1], Xuesong Zhang[1], Musen Li[1], Yongjie Shen[5], Wenqiang Qu[6], Zhanchen Wang[1], Yi Jiang[1], Shuai Guo[7], Pengcheng Xu[4], Zhenggang Xue[1,], Dengsong Zhang[1], Jiaqiang Xu[1]*

Table of contents

Experimental section:

Supplementary Notes: 1-3

Supplementary Figures: 1-64

Supplementary Tables: 1-6

References:

Experimental section

Materials.

Platinum bis(acetylacetonate) ($Pt(acac)_2$),tin(II) chloride dehydrate ($SnCl_2 \cdot 2H_2O$), poly(vinylpyrrolidone)(PVP, M.W. 30000/1000000), N,N-Dimethylformamide (DMF), ascorbic acid(AA) and ethanol (C_2H_5OH) were analytical grade and purchased from Shanghai Chemical Corp. All reagents in the experiment were used without any further purification. Gases including nitrogen dioxide (NO_2), sulfur dioxide (SO_2), dinitrogen monoxide(N_2O), ammonia (NH_3), hydrogen sulfide (H_2S), carbon monoxide (CO), hydrogen (H_2), carbon dioxide (CO_2), benzene (C_6H_6) and xylene (C_8H_{10}) were obtained from Shanghai Shenkai Gases Technology Co., Ltd

Synthesis of SnO_2 nanofibers.

In a typical synthesis process, the electrospinning precursor solution of SnO_2 was first prepared by dissolving 1.2 g of tin(II) chloride dihydrate ($SnCl_2 \cdot 2H_2O$) and 1.6 g of polyvinylpyrrolidone (PVP, M.W. 1000000) in a mixed solvent of 9 mL of N,N-Dimethylformamide (DMF) and 2 mL of ethanol, followed by stirring for 48 hours. The electrospinning parameters were set as follows: a voltage of 16 kV between the needle and the collector, a fixed distance of 15 cm, and a solution flow rate of 0.5 mL/h. The electro spun product was initially stabilized at 200 °C for 1 hour. Subsequently, it was heated in a tube furnace at 900 °C for 2 hours under an air atmosphere. The resulting white powder was collected and labeled as SnO_2.

Synthesis of Pt_1-SnO_2.

200 mg of the as-synthesized SnO_2 powder was uniformly dispersed in a mixed solution containing 20 mL of ethanol and 20 mL of deionized water. A specific concentration of platinum (II) acetylacetonate (Pt(acac)$_2$)) solution in ethanol (10 mg/mL, 0.2 mL) was then added dropwise into the mixed solution at a rate of 0.5 mL/h. The mixture was stirred at room temperature for 24 hours. Subsequently, the product named Pt_1-SnO_2 was collected by centrifugation and dried in a vacuum oven at 70 ºC for 10 hours.

Synthesis of Pt NPs-SnO_2.

A quantity of 4 mg of platinum (II) acetylacetonate (Pt(acac)$_2$) and 6 mg of ascorbic acid (AA) were weighed and added to a mixed solvent composed of 20 mL of ethanol and 20 mL of deionized water. The mixture was stirred for 2 hours until complete dissolution. Subsequently, 200 mg of the as-synthesized SnO_2 powder was added to the above solution. The resulting mixture was stirred at room temperature for 24 hours. The final product was then collected by centrifugation, dried in a vacuum oven at 70 °C for 10 hours, and labeled as Pt NPs-SnO_2.

Synthesis of outer-1%-PtSn-Sn NFs.

A quantity of 200 mg of the as-synthesized SnO_2 sample was uniformly dispersed in a mixed solvent composed of 20 mL of ethanol and 20 mL of deionized water. Subsequently, 2 mg of the Pt_3Sn alloy was added to the mixture, which was then stirred

at room temperature for 24 hours. The resulting solid mixture was collected by centrifugation at 10,000 rpm/min, dried in a vacuum oven at 70 °C, and subsequently transferred to a ceramic boat. The sample was heated in a tube furnace at 900 °C for 2 hours under an air atmosphere, with a controlled heating rate of 5 °C/min. The final product was labeled as outer-1%-PtSn-Sn NFs.

Gas cell in-situ TEM experiment.

The in-situ TEM characterization was performed on the Tecnai F20 TEM with a commercial in-situ gas holder (Climate S3, DENS solutions)[1,2]. A gas supply system controls the gas flow. To conduct the in-situ TEM experiments, we introduced the air gas, (1 mL/min, respectively) to the gas cell chips with a controlled pressure of ~1.0 bar to mimic the working atmosphere of sensing material and the reaction temperature was well controlled by adjusting the heating voltage of the integrated micro-heater on the chip. The electron dose rate of the in-situ TEM experiments was 10 e^-/$Å^2$/s, and the total dose was 6000 e/ $Å^2$. In order to estimate the selected nanoparticle's projection area, the mean diameter of the nanoparticle is obtained based on the data measured from different directions (n>3) and, the nanoparticle is treated as a perfect sphere to calculate the area.

XAFS Measurements.

The X-ray absorption fine structure data were collected at 1W1B station in Beijing Synchrotron Radiation Facility (BSRF). The storage rings of BSRF were operated at

2.5 GeV with a maximum current of 250 mA. The X-ray absorption near edge structure (XANES) data were recorded in fluorescence mode. All samples were pelletized as disks of 13 mm diameter using poly(1,1-difluoroethylene) powder as a binder. The acquired extended X-ray absorption fine structure (EXAFS) data were processed according to the standard procedures using the ATHENA module implemented in the IFEFFIT software packages[3]. The EXAFS spectra were obtained by subtracting the post-edge background from the overall absorption and then normalizing with respect to the edge-jump step. Subsequently, $\chi(k)$ data in the k-space were Fourier transformed to real (R) space using a Hanning window (dk = 1.0 $Å^{-1}$) to separate the EXAFS contributions from different coordination shells.

Fabrication of MEMS sensors and Gas-sensing measurement.

The MEMS sensor with an integrated micro heater and interdigital electrode adopts a suspended membrane structure with low power consumption and high reliability. Interdigital electrode (pin 1, 2) is used to detect resistance change of gas sensitive material, and heater electrode (pin 3, 4) is used to provide a suitable working temperature for a gas sensor, as shown in Fig. S27. The resistance value of the heating chamber (2, 4 pins) is 45 ± 5 Ω. The sensor was fabricated according to our previous method [4,5]. First, the prepared samples and ethanol were mixed in an agate mortar to make a paste, and then a drop of the mixed sample was dropped onto a Pt interdigital electrode (1.0 mm × 1.0 mm × 0.5 mm) by micropipette. After dried naturally at room

temperature, a resistance-type sensor was obtained. Subsequently the prepared MEMS sensors were sintered at 150 °C for 5 h to improve their stability. The MEMS sensors were placed on the base with the heating electrode and test electrode.

The gas sensing tests were performed on an intelligent gas sensing analysis system of EVM-HP-MC02 (Shanghai Lingpan Electronic Technology Co., Ltd., China). The gas sensing system were shown in Fig. S28. The operating voltage of the test circuit is 5 V. The resistance of sensors was obtained by detecting the voltage of the loading resister in series. The resistances (R) of sensors were calculated by the Eq. (1):

$$R = \left(\frac{5-Vout}{Vout}\right) \cdot R_L \tag{1}$$

Where 10 is the circuit voltage, V_{out} is the output voltage of the load resistance (R_L) detected. The operating temperature of sensors was achieved by adjusting the heater voltage. When the resistances kept stable, the test gas was injected into the 200 mL chamber. The amount of injected gas can be obtained through the Eq. (2):

$$C_x = \frac{V_x}{200} \cdot C \tag{2}$$

Where V_x(mL) is the gas volume extracted from the gas sampling bags by the micro syringe, 200 is the volume of the test chamber, C is the standard concentration of gas in gas sampling bags and the C_x is the different gas concentration in the test chamber. The response value of sensors in an oxidizing atmosphere is defined as Eq. (3):

$$R_{response} = R_g / R_a \tag{3}$$

The response value of sensors in a reducing atmosphere is defined as Eq. (4):

$$R_{response} = R_a / R_g \tag{4}$$

The resistance of the sensor in the air is recorded as R_a and that in the test gas is R_g.

For the cross-sensitivity and selectivity tests, two/three gases were simultaneously injected into the chamber to simulate complex gas environments. The concentrations of the target gas (NO_2) and interfering gases (e.g., H_2S, SO_2) were calculated based on the injection volume and the total chamber volume (V = 200 mL).

Fabrication of Quartz Crystal Microbalance (QCM)and sensing test process.

The QCM test was prepared using a drop-coating method. Before coating the samples, the electrodes of the QCM were sonicated in acetone for 10 min, followed by an ethanol/water mixture in a 1:1 ratio for 30 min. The electrodes were then dried at 60°C to ensure that the QCM was clean and free of impurities. Subsequently, the three as-synthesized samples were each dispersed in distilled water to form suspensions with a concentration of 1 mg/mL. Next, 2 μL of each suspension was drop-cast onto one side of the QCM electrode, and the prepared sensors were then placed in a vacuum oven for 2 h. To ensure uniformity of the sensitive membrane on the QCM electrode, a shell was used to cover all parts of the QCM except for the electrodes. The specific device is illustrated in Fig.S43.

The test process of the QCM sensor involves three steps: (1) Establishing a baseline by purging with high-purity synthetic air; the target gas is introduced only after the baseline stabilizes (frequency drift does not exceed 5 Hz). (2) A gas with a pre-calculated concentration is extracted using a micro-sampler and quickly injected into

the test chamber. Once the adsorption capacity reaches a maximum and the curve stabilizes, the system reaches an adsorption-desorption equilibrium, and the frequency change represents the response value. (3) Purging with high-purity synthetic air desorbs the adsorbed gas from the sensing materials, causing the frequency value to return to the baseline. The above operations are repeated for different gas concentrations to obtain continuous response curves. The testing chamber was set in a heating oven to maintain a stable temperature, and all measurements were conducted at room temperature (25.0 ± 1.0°C). The relative humidity was controlled at 50% using a dehumidifier.

A quantitative relationship exists between an increase in mass change (Δm) on the QCM surface electrode and a decrease in frequency change (Δf), which satisfies the Sauerbrey equation[6]:

$$\Delta f = -2.3 \times 10^{-6} \times f_0^2 \times \Delta m/A. \quad (5)$$

Where Δf is the frequency variation value (unit: Hz), the f_0 is the inherent oscillation frequency of the QCM chip (10×10^6, unit: Hz), A is the work area of the electrodes (d=3 mm). Δm is the mass change caused by the adsorbed gas molecules on the electrode, (unit: Hz), the f_0 is the inherent oscillation frequency of the QCM chip (10×10^6, unit: g).

Theoretical calculation.

Spin-polarized periodic density functional theory (DFT) calculations were performed within the generalized gradient approximation (GGA) using the Perdew–Burke–Ernzerhof (PBE) [7,8] exchange–correlation functional, as implemented in the Vienna ab initio Simulation Package (VASP). Van der Waals interactions were accounted for using the DFT-D3 method with Becke–Johnson damping [9]. The interactions between ionic cores and valence electrons were described using the projector-augmented wave (PAW) method. The valence electronic states were expanded in a plane-wave basis set with a kinetic energy cutoff of 420 eV. Structural optimizations were carried out until the residual forces on each atom were less than 0.02 eV $Å^{-1}$.

The SnO_2 (110) surface was modeled using a slab consisting of three atomic layers with a vacuum thickness greater than 15 Å, constructed in a 6 × 3 × 1 supercell. Pt-doped SnO_2 models (denoted as Pt_1-SnO_2 and Pt_n-SnO_2) were generated by substituting the corresponding Sn atoms in the topmost layer with Pt atoms. The Pt-SnO_2 (110)/NO_2 interface was constructed by introducing 64 NO_2 molecules onto the Pt-SnO_2 (110) surface.

During ab initio molecular dynamics (AIMD) simulations, a 1 × 1 × 1 Γ-centered k-point mesh was employed. The top atomic layer was allowed to relax, while the remaining layers were fully fixed. AIMD simulations were performed in the canonical (NVT) ensemble at 363 K using a Nosé-Hoover thermostat, with a total simulation time of 12 ps and a time step of 1.0 fs. The concentration profiles were obtained by averaging over 4,000 configurations collected from the last 8-12 ps of the AIMD trajectory.

Molecular Dynamics (MD) Simulation.

According to the in-situ TEM and TGA results, the formation of SnO_2 support concentrate in 500 °C, while the final oxidation of Sn species in Pt_3Sn alloys completes at 610 °C. The initial configuration of the system is built by SnO_2, Sn_3Pt and O gas phase. The crystal structures of SnO_2 and Sn_3Pt were constructed from unit cells obtained from the Materials Projects crystallographic dataset[10,11]. Oxygen atoms were placed above the solid surfaces to simulate the gas-solid interface. In total, 657 atoms were included in the simulation box with 1.92 nm × 3.35 nm × 3.50 nm, composed of 51 Pt atoms, 160 Sn atoms, and 392 oxygen atoms. Among them, 54 oxygen atoms were treated as gas-phase O atoms positioned above the surface. The force field parameters between all atoms were modeled by a LJ-type potential, obtained from openkim.org[12,13].

Molecular dynamics (MD) simulations were performed using the LAMMPS package[14]. A total of 1×10^6 timesteps were performed with a timestep of 0.0001 ps. Data were collected every 0.1 ps for production runs and subsequent analysis. All simulations were conducted in the NVT ensemble at a temperature of 1173.15 K, with periodic boundary conditions applied in all three directions. Visualization and post-processing of atomic trajectories were performed using OVITO[15].

Details of activation energy.

The activation energy refers to the energy required to transition from a normal state to an active state that is prone to chemical reactions. In 1889, Arrhenius revealed the relationship between reaction rate and temperature through extensive experimental and theoretical demonstration. The Arrhenius equation takes the following form:

$$k = Ae^{-\frac{E_a}{RT}} \quad (6)$$

$$lnk = lnA - \frac{E_a}{RT} \quad (7)$$

The relationship between lnk and $-\frac{1}{T}$ is linear, with a slope of - $\frac{E_a}{R}$. The k value at different temperatures is measured by experiments.

$$E_a = R \times slope$$

So, the *E* value can be obtained by plotting lnk against $\frac{1}{T}$.

where k is the reaction rate. *A* is the pre-exponential factor, E_a is the activation energy. T is the operating temperature and *R* is the molar gas constant (8.314 $J \cdot mol^{-1} \cdot K^{-1}$).

For this article, The Arrhenius equation is described as follow:

$$\tau_{res} = A_0 \, exp[-E_{act}/RT] \quad (8)$$

where τ_{res} is the reaction rate. A_0 is the pre-exponential factor, E_{act} is the activation energy. T is the operating temperature and *R* is the molar gas constant (8.314 $J \cdot mol^{-1} \cdot K^{-1}$).

Supplementary Notes 1: Sample preparation and in-situ TEM gas cell setup

The as-synthesized sample was dispersed in ethanol via ultrasonication. A precise amount of the suspension was transferred onto the observation window of the lower chip using a quartz capillary. The chip was then dried under an infrared lamp for 10 minutes to ensure complete evaporation of ethanol. Optical images of the upper and lower chips are shown in Fig. S63. The observation window of the chip consisted of a 30 nm-thick silicon nitride membrane. The lower and upper chips were aligned face-to-face using a microscope and alignment tools, followed by assembly onto a commercial gas-phase in situ heating holder to form a sealed gas cell. After confirming the gas-tightness of the cell, the system was ready for in-situ TEM observations of sample.

The sample holder was gently inserted into the pre-pumping chamber of the transmission electron microscope (TEM). The pre-pumping procedure was initiated until the vacuum reached the required level, after which the holder was transferred into the main TEM chamber. The gas inlet and outlet of the chip were connected to a commercial gas supply system (Model: Climate S3, DENS solutions) via capillaries integrated into the holder. The atmosphere within the microreactor (gas cell) was controlled as air, with a flow rate of 1.0 mL/min and a pressure of 1 bar. Electrical contacts on the chip were connected to external circuits through probes on the holder, enabling precise temperature control. The heating program was initiated with a ramp

rate of 30 °C/min from room temperature to 900 °C, followed by a 5-minute hold at 900 °C. The structural dynamics of the material during gas-solid reactions were monitored using a high-resolution TEM (Model: FEI TCG2 F20) operated at 200 kV, equipped with a CMOS camera (EMSIS XAROSA) capable of continuous video recording. Simultaneously, energy-dispersive X-ray spectroscopy (EDS; Oxford X-Max) was employed to perform elemental mapping and quantitative analysis of the sample before and after the reaction.

Supplementary Notes 2: Principal components analysis (PCA) for pattern recognition.

The principal components analysis (PCA) was used for pattern recognition to classify multivariant sensor data. PCA is a method of finding the axis that best describes all variables by obtaining the covariance matrix and performing eigenvalue decomposition. A data matrix for single analyte was constructed by gas response data from nine sensors ((1) SnO_2, (2) Pt_1-SnO_2, (3) Pt NPs-SnO_2, (4) 0.3%-PtSn-SnO_2, (5) 0.6%-PtSn-SnO_2, (6) 1%-PtSn-SnO_2, (7) 1%-PtSn-SnO_2, (8) 1.5%-PtSn-SnO_2, and (9) 2%-PtSn-SnO_2) for 3, 5, 10 ppm of NO_2, SO_2, H_2S, N_2O, NH_3, H_2, C_6H_6, C_8H_{10}.

From this matrix, the eigenvalue and eigenvector were calculated. Each observation was projected onto this eigenvector line to get a coordinate value along the principal component line. The first principal component (PC1) represents the maximum variance direction in the data. A second PC (PC2) exhibits the second-largest variance along the direction orthogonal to PC1. PC1 and PC2 were plotted in Fig. S48 and Fig. 5c.

Supplementary Notes 3: The Cruising experiments of humanoid robot integrated with sensor array.

As shown in Fig. 6c and Fig.S64, the system we develop is fixed on the chest of humanoid robot (R1, Unitree Robotics). The standing dimensions of the humanoid robot are 1320 mm×450 mm×200 mm. The Unitree R1 in the as-proposed robotic application is a 23 degrees-of-freedom quadruped robot. The robot is capable of omnidirectional movement within a 2D environment and demonstrates excellent shock resistance and balancing stability.

In the experiment, four boxes with same shape (cuboid) and size (200 mm × 200 mm × 290 mm) are placed in a row in a specific field. A transparent exhaust pipe connected to a gas cylinder is integrated at the front of the box 1 and box 2. And there are double-tube connected to the gas cylinder in the box 3 and box 4. The robot will move through the specific routes and the olfactory sensor array approached target boxes to monitoring the gases in a cavity.

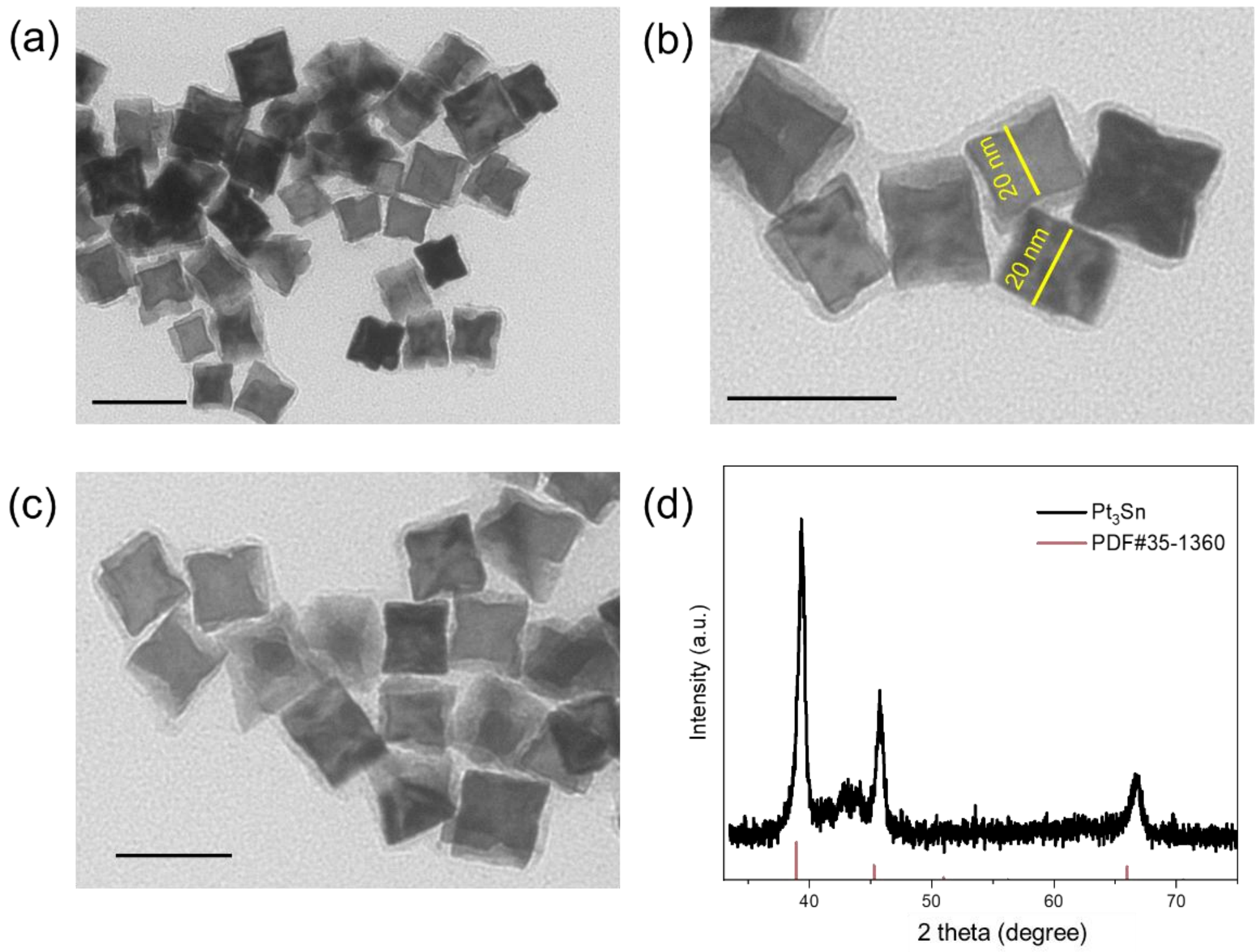

Supplementary Figure 1. (a-c) The TEM images of Pt_3Sn alloys (scale bar: 50 nm). (d) XRD pattern of Pt_3Sn alloys.

As observed in the Fig. S1(a-c), the Pt_3Sn alloy particles exhibit a uniform cubic morphology with a diameter in the range of 20 nm. The XRD pattern (Fig. S1(d)) of the sample exhibits three diffraction peaks at 38.9°, 45.1°, and 65.7°, which correspond to the (111), (200), and (220) crystal planes of the Pt_3Sn alloy.

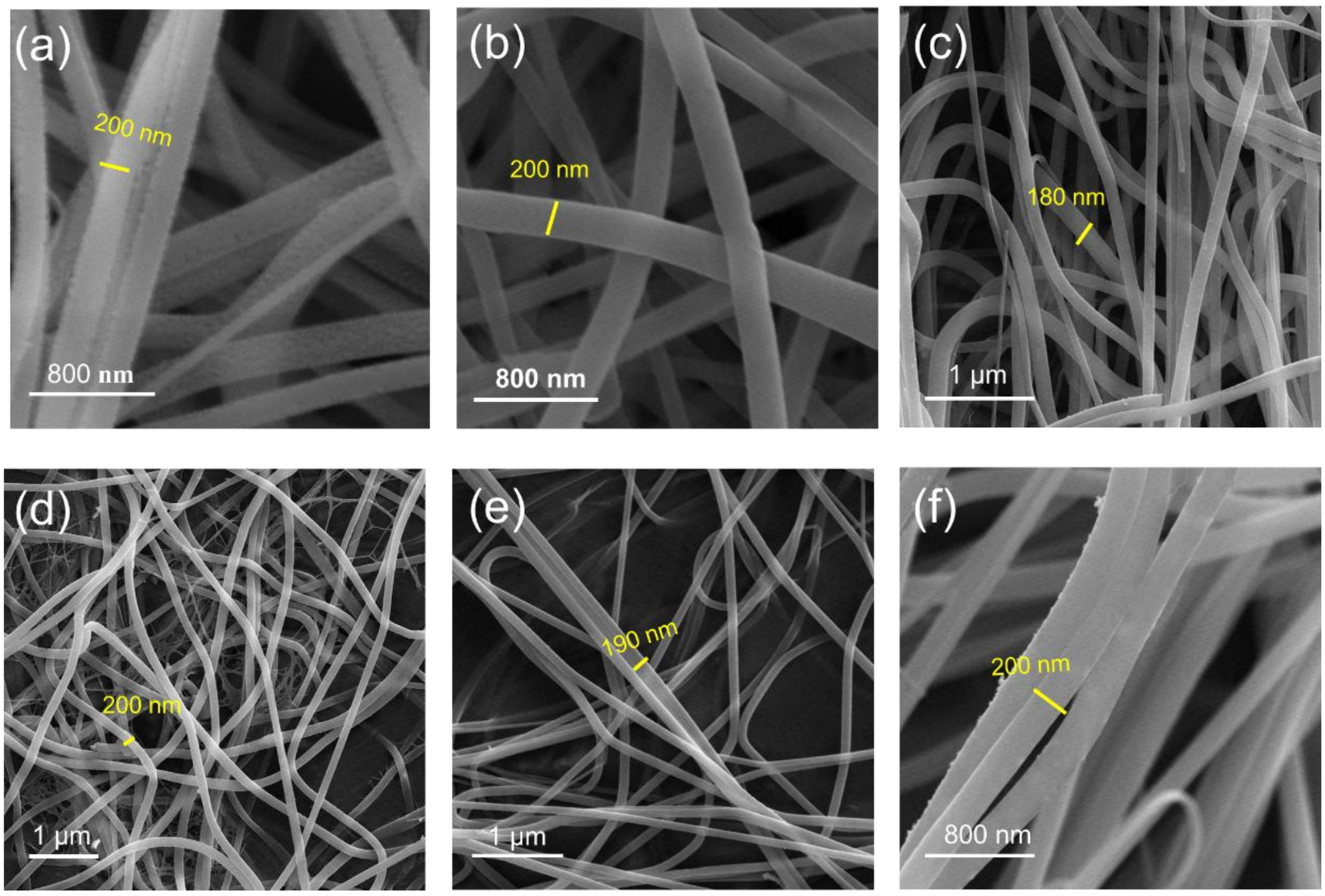

Supplementary Figure 2. SEM images of (a) Sn NFs, (b) 0.3%-PtSn-Sn NFs, (c) 0.6%-PtSn-Sn NFs, (d) 1.0%-PtSn-Sn NFs, (e) 1.5%-PtSn-Sn NFs, (f) 2.0%-PtSn-Sn NFs.

The introduction of Pt_3Sn alloys does not significantly alter the fiber morphology or diameter. This is attributed to the fact that the electrospinning process is primarily dictated by the rheological properties of the polymer matrix, which remain stable despite the trace amount of metal doping.

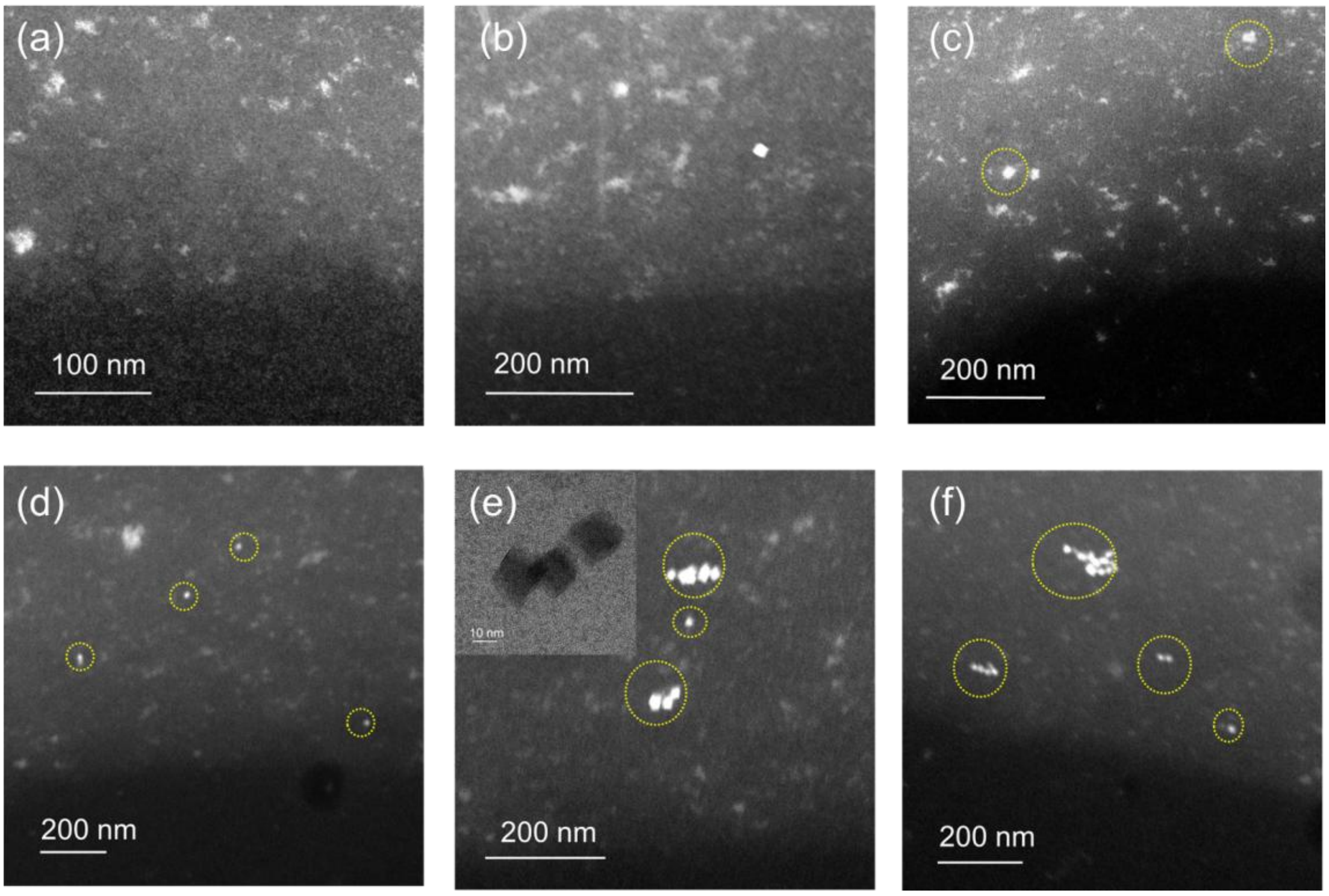

Supplementary Figure 3. TEM images of (a) Sn NFs, (b) 0.3%-PtSn-Sn NFs, (c) 0.6%-PtSn-Sn NFs, (d) 1.0%-PtSn-Sn NFs, (e) 1.5%-PtSn-Sn NFs, (f) 2.0%-PtSn-Sn NFs.

The TEM images (Fig. S3) clearly show that the Pt_3Sn particles are loaded on the nanofibers. When the loading mass of the Pt_3Sn alloy exceeds 1.5%, significant agglomeration of the alloy particles is observed.

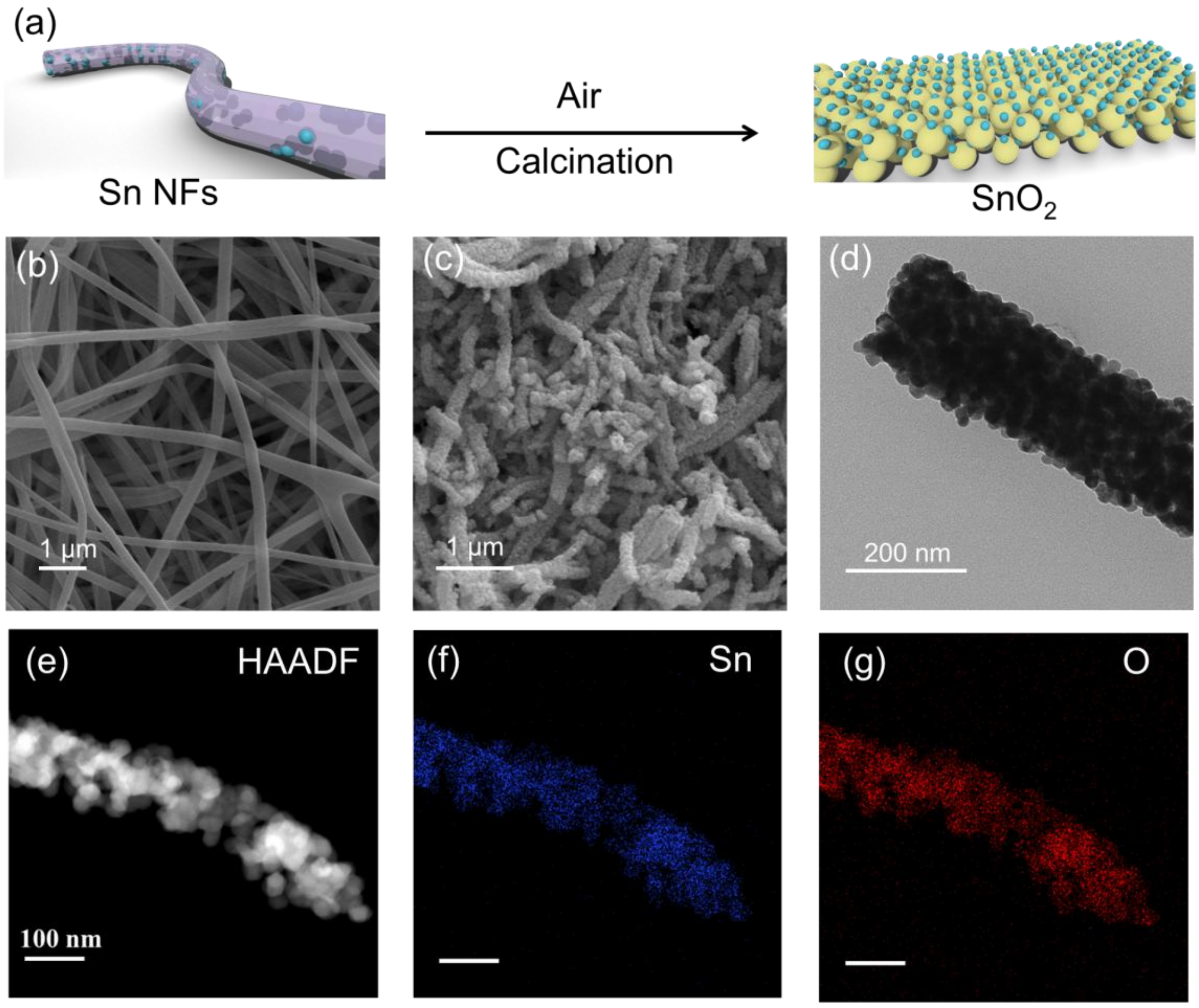

Supplementary Figure 4. (a) Schematic of the synthesis process of SnO_2. (b) The SEM image of Sn NFs. (c) The SEM image of SnO_2. (d) The TEM image of SnO_2. (e-g) The EDS elements (Sn and O) mapping images of SnO_2 (scale bar: 100 nm).

The SnO_2 nanorods were obtained through the pyrolysis of Sn NFs in air (Fig. S4(a)). As shown in Fig. S4(b), the Sn NFs possess a diameter of approximately 200 nm. After calcination, the resulting nanorods are composed of granular SnO_2 particles, as revealed in Fig. S4(c, d). EDS mapping results further demonstrate that Sn and O elements are uniformly distributed across the surface of the SnO_2 sample (Fig. S4(e-g)).

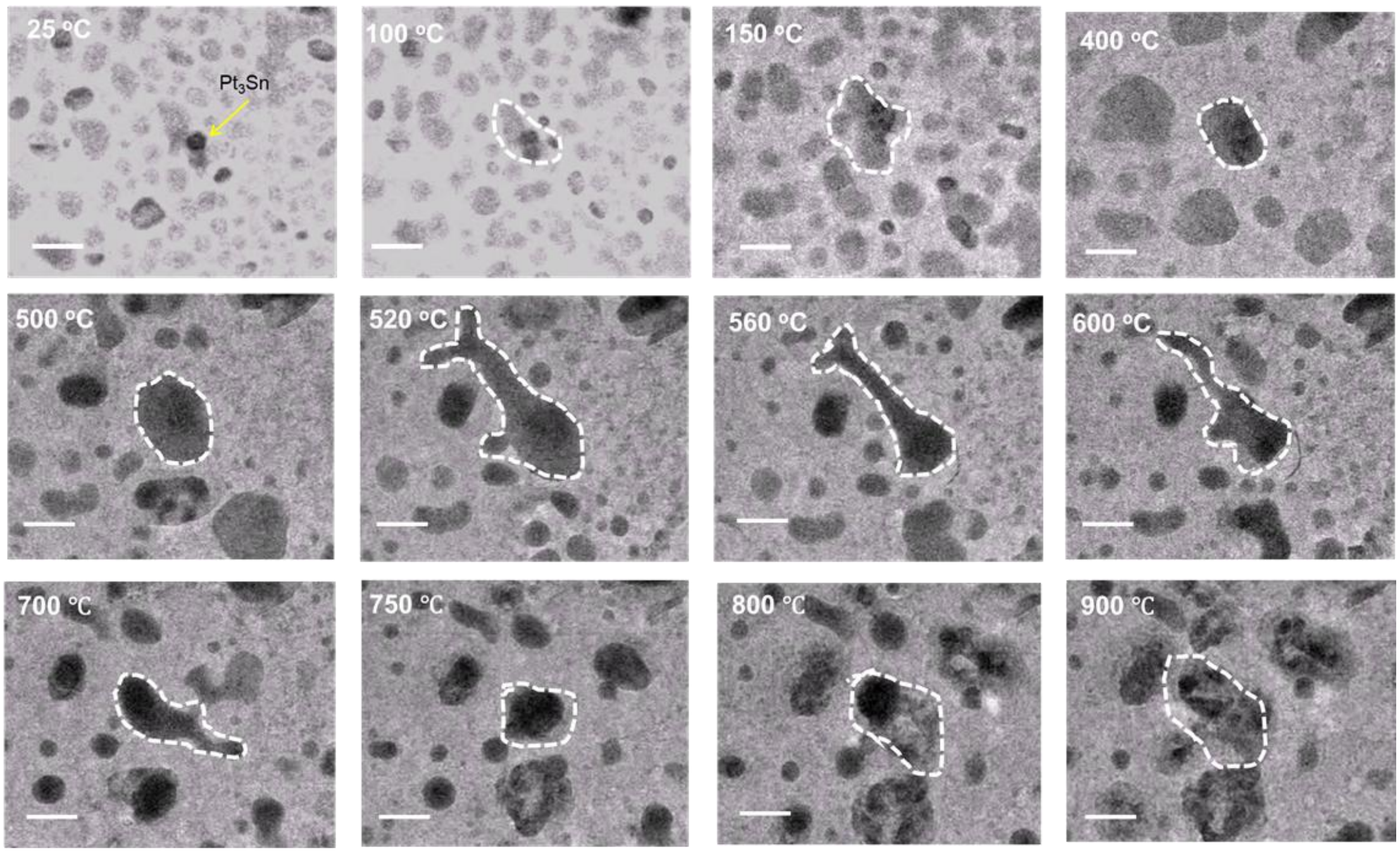

Supplementary Figure 5. In-situ TEM images of 1%-PtSn-SnO_2.

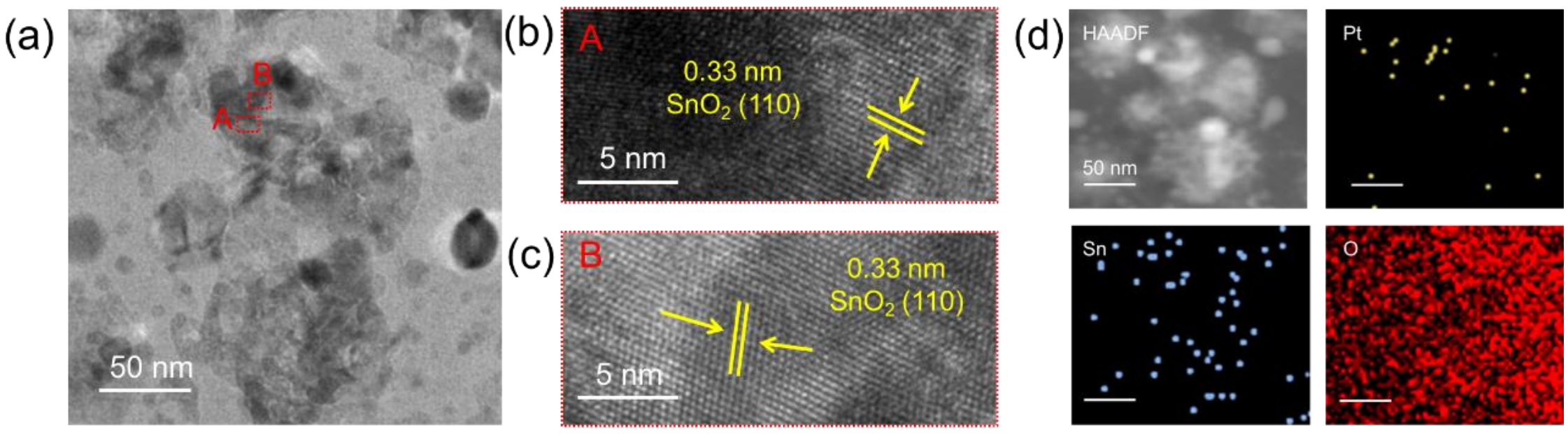

Supplementary Figure 6. (a) TEM image of 1%-PtSn-Sn NFs after 900ºC treatment. The HRTEM image of (b) region A and (c) region B. (d) EDS-mapping of 1%-PtSn-Sn NFs after 900ºC treatment.

The sample subjected to in-situ TEM processing was characterized by high-resolution transmission electron microscopy (HRTEM) in Fig. S6(a). As shown in Fig. S6(b, c), the measured interplanar spacing of 0.33 nm in regions A and B of the sample corresponds to the (110) plane of SnO_2. And the EDS-mapping analyses (Fig. S6(d)) indicate that no aggregated Pt particle is detected on the surface of sample.

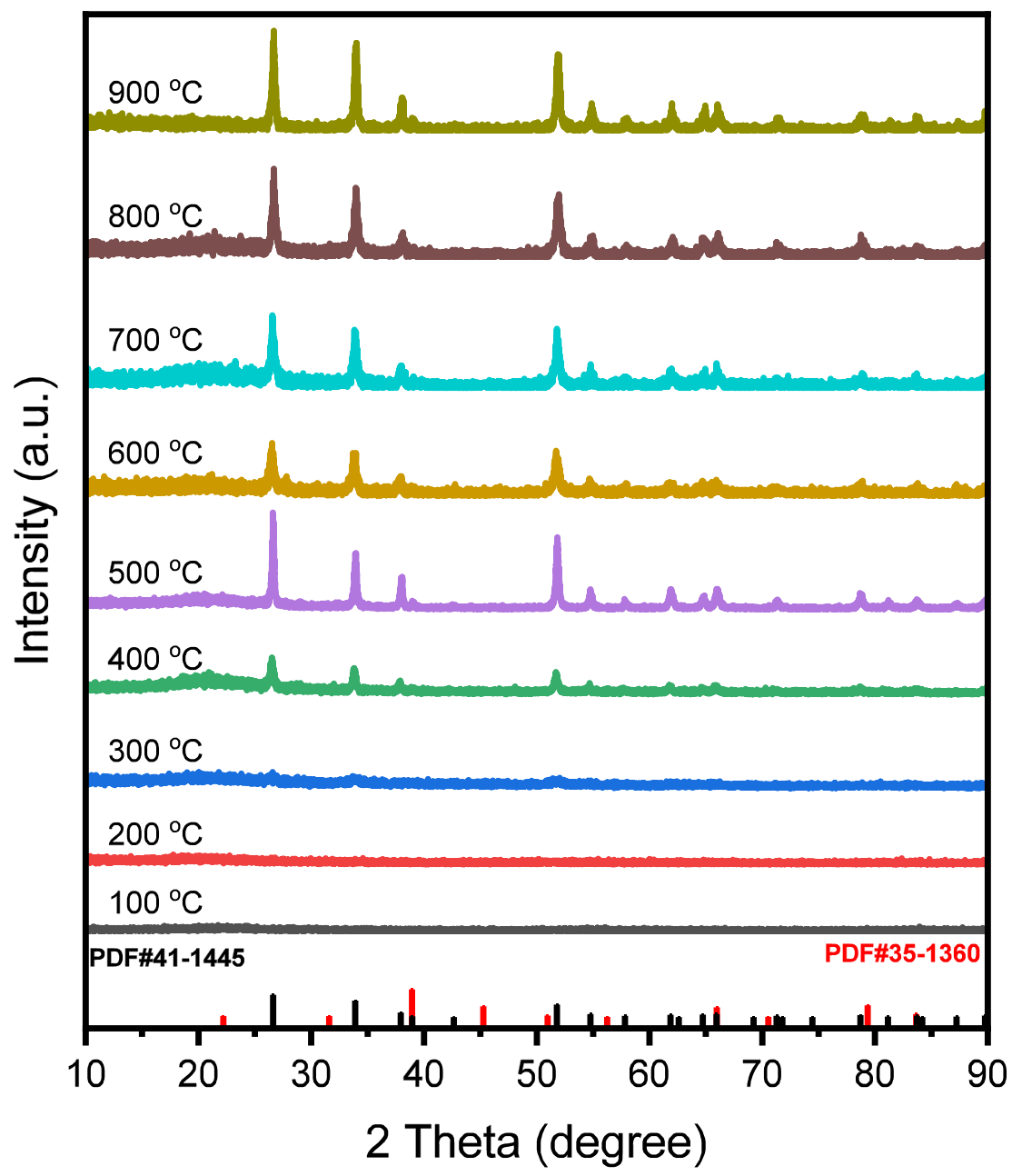

Supplementary Figure 7. XRD spectra of 1%PtSn-Sn NFs after different temperature treatment. (JCPDS No. 41-1445: SnO_2 and JCPDS No. 65-1323: Pt_3Sn)

With the increasing temperatures, the 1%PtSn-Sn NFs exhibit the characteristic diffraction peaks of SnO_2 at 300 °C. Within the temperature range of 500 to 900 °C, the crystallinity of SnO_2 enhances. The three characteristic peaks located at approximately 26.5°, 33.7°, and 51.7° correspond to the (110), (101), and (211) crystal planes of SnO_2, respectively.

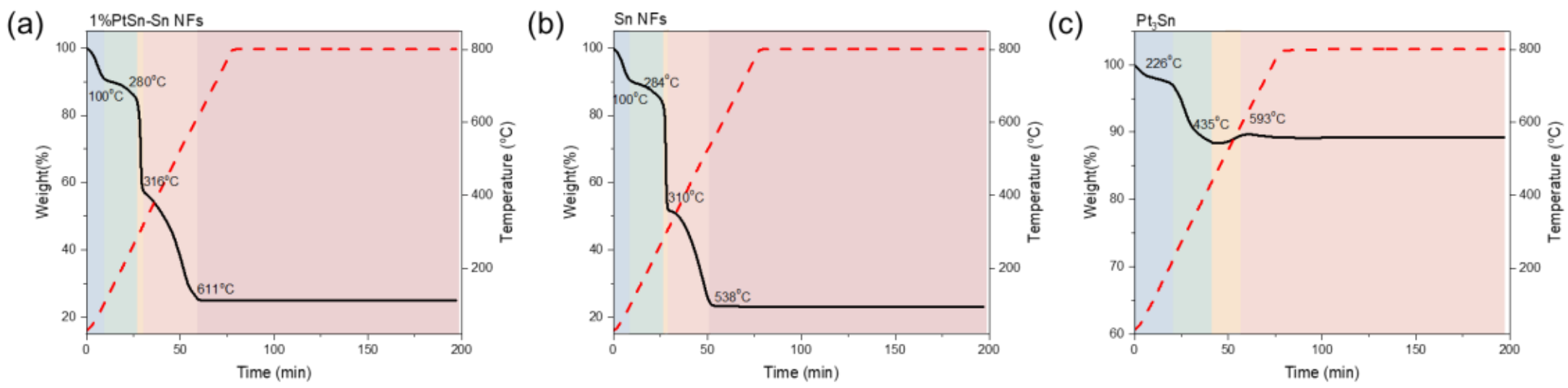

Supplementary Figure 8. The TGA curves of (a) 1%PtSn-Sn NFs, (b) Sn NFs and (c) Pt_3Sn alloys.

For the Sn NFs sample, the mass loss observed below 284 °C is attributed to the evaporation of water molecules and adsorbed organic solvents. The significant mass loss between 284 °C and 538 °C is ascribed to the decomposition of polymers within the nanofibers and the oxidation of residual carbon. Above 538 °C, the mass remains stable, indicating the complete formation of crystalline SnO_2. For the Pt_3Sn sample, the initial mass loss observed between 226-593 °C is attributed to the profound decomposition of the alloy and the combustion of residual carbon, while the subsequent gradual mass increase is due to the oxidation of the metallic components. For the 1%-PtSn-Sn NFs, the temperature required for the formation of a stable inorganic phase was delayed to 611 °C compared to the pure Sn NFs. This retardation is likely attributed to the endothermic nature of the redispersion process of the Pt_3Sn alloy on the SnO_2 surface.

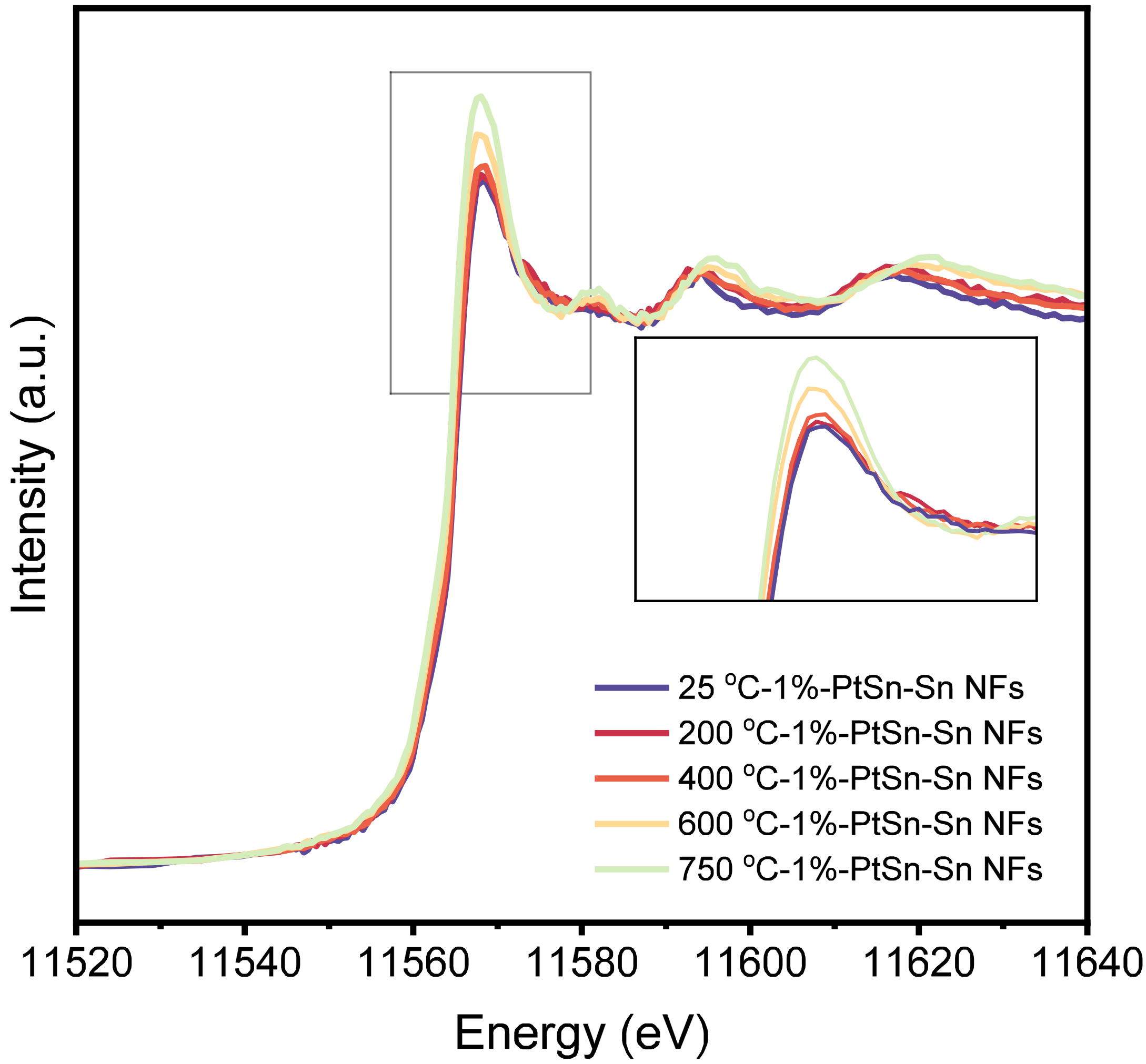

Supplementary Figure 9. XANES spectra of 1%-PtSn-Sn NFs after different temperature treatment.

The white line intensities of the five samples follow the order: 750 ºC > 600 ºC > 400 ºC > 200 ºC > 25 ºC 1%PtSn-Sn NFs. This trend indicates that the oxidation states of Pt increase progressively with higher heating temperature.

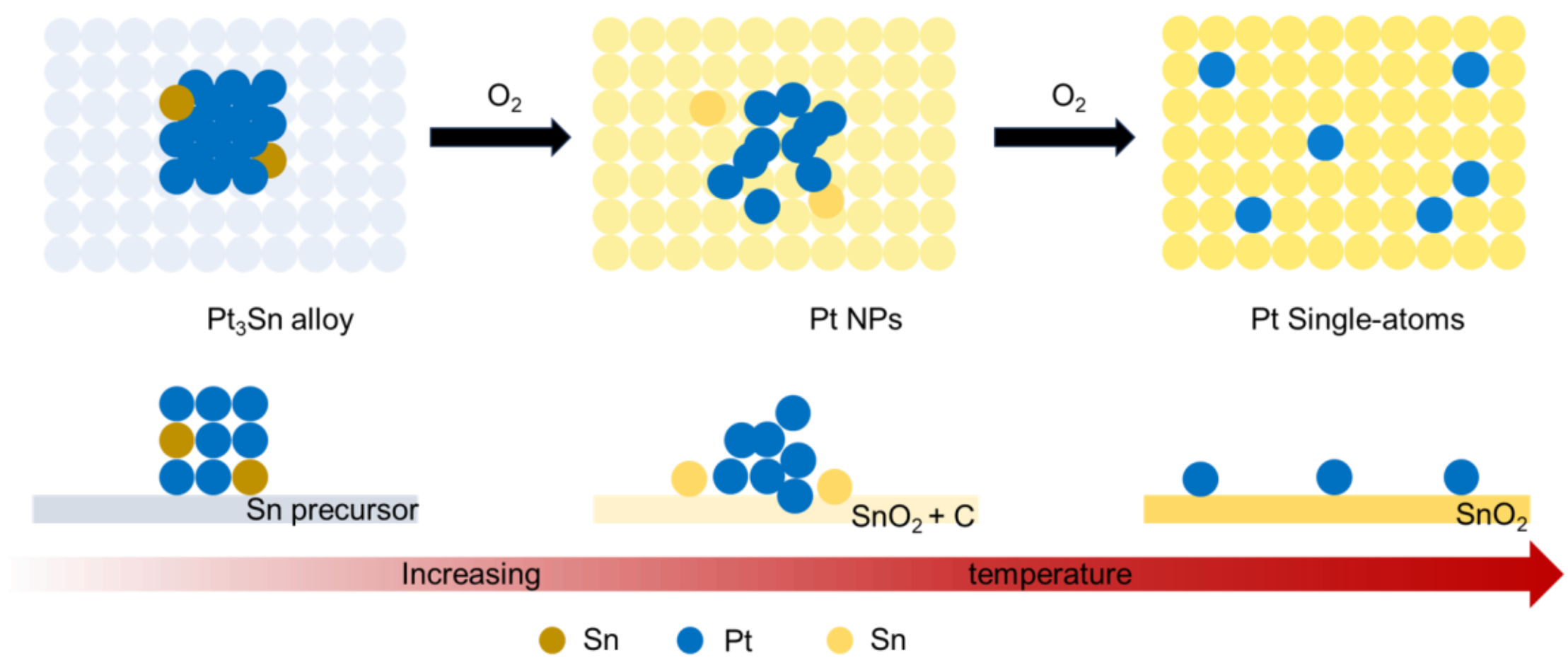

Supplementary Figure 10. Schematic illustration for the synthesis of 1%-PtSn-SnO_2.

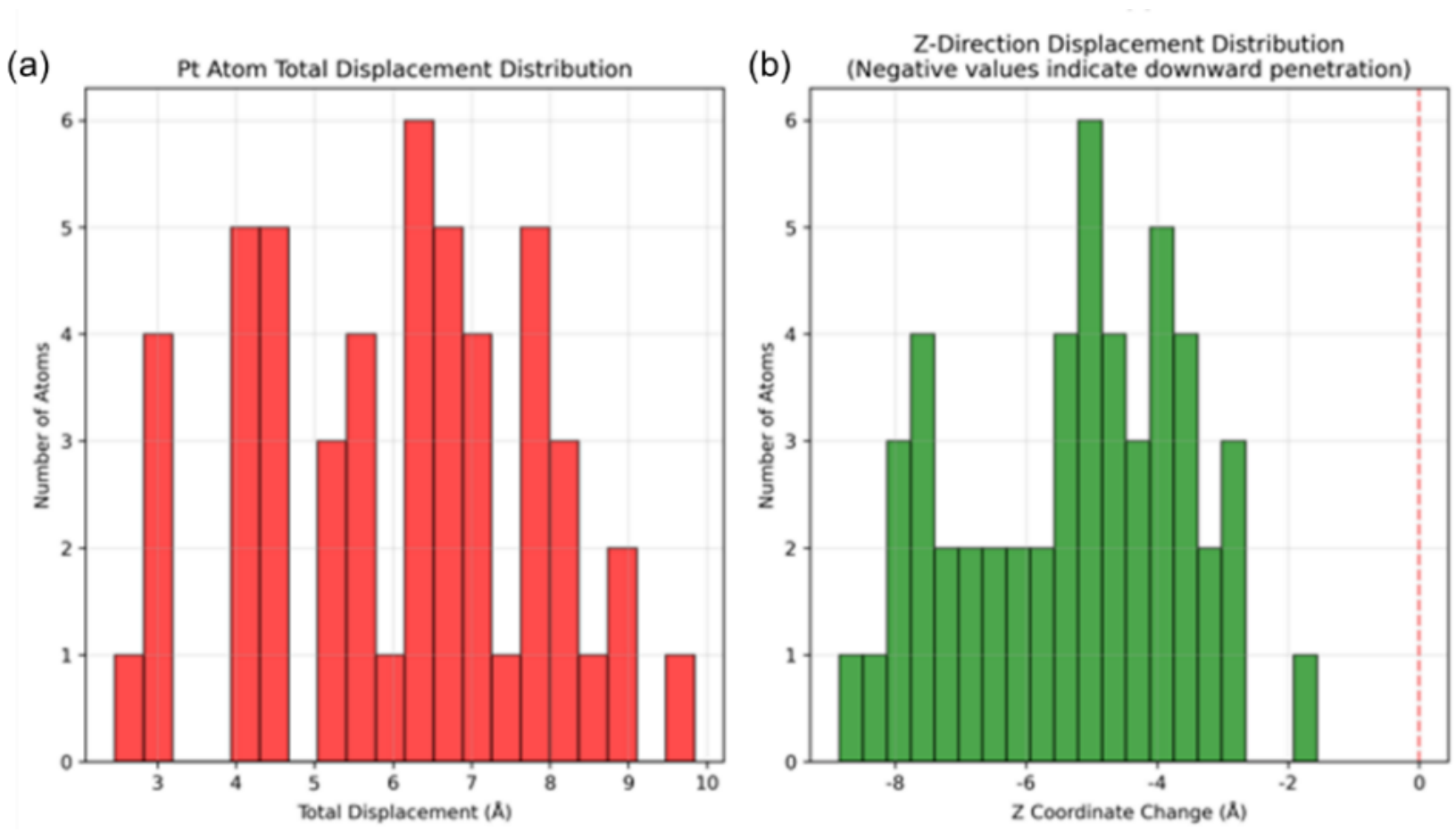

Supplementary Figure 11. Analysis of the results obtained from the MD simulation. (a) The total displacement distribution of Pt atoms. (b) Z-direction distribution.

Analysis of the total displacement distribution of Pt atoms and the distributed changes in their Z-coordinates reveal that Pt atoms gradually penetrate from the Pt_3Sn alloy into the SnO_2 support during the MD simulation.

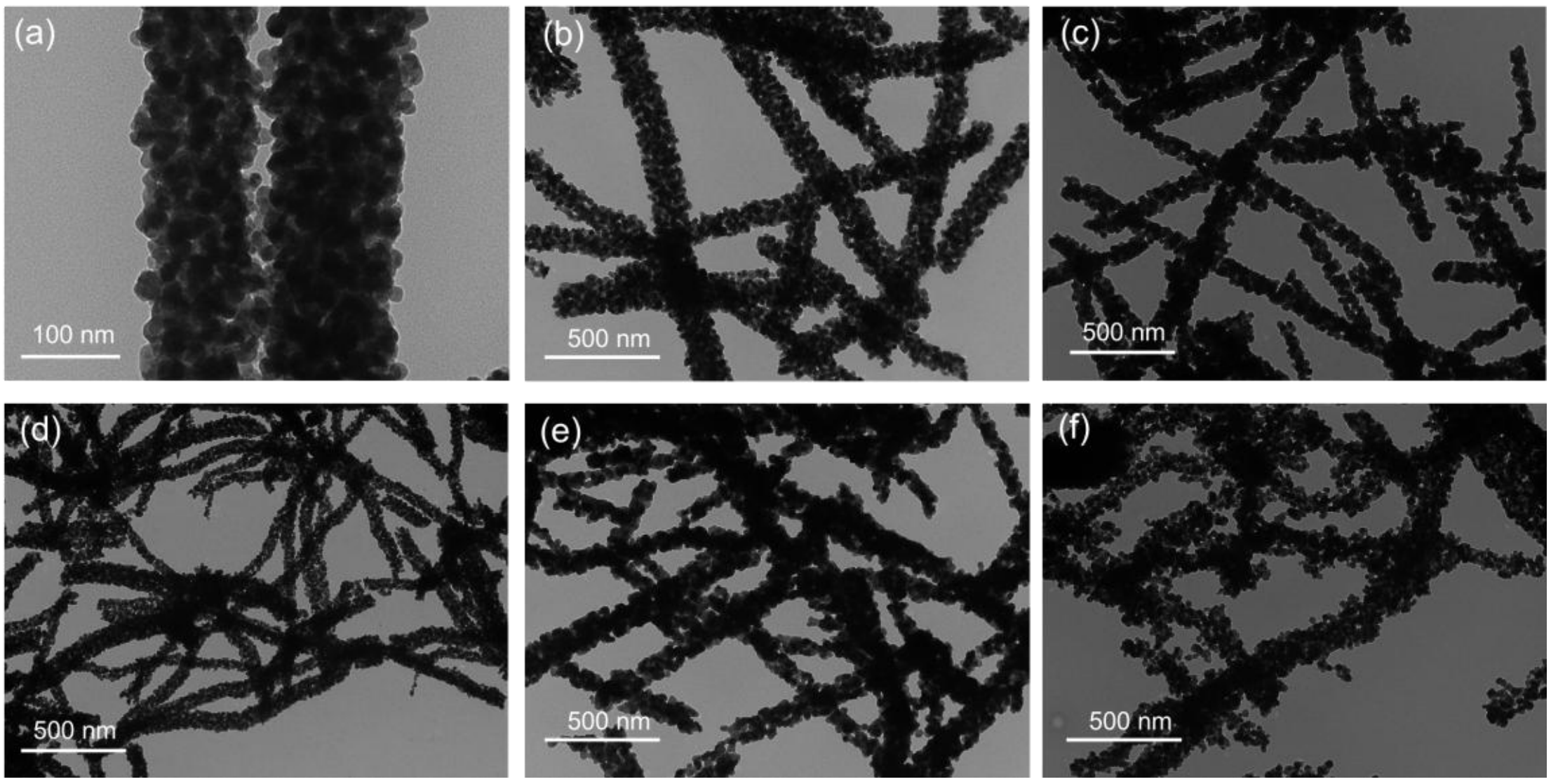

Supplementary Figure 12. TEM images of (a) SnO_2, (b) 0.3%-PtSn-SnO_2, (c) 0.6%-PtSn-SnO_2, (d) 1.0%-PtSn-SnO_2, (e) 1.5%-PtSn-SnO_2, (f) 2.0%-PtSn-SnO_2.

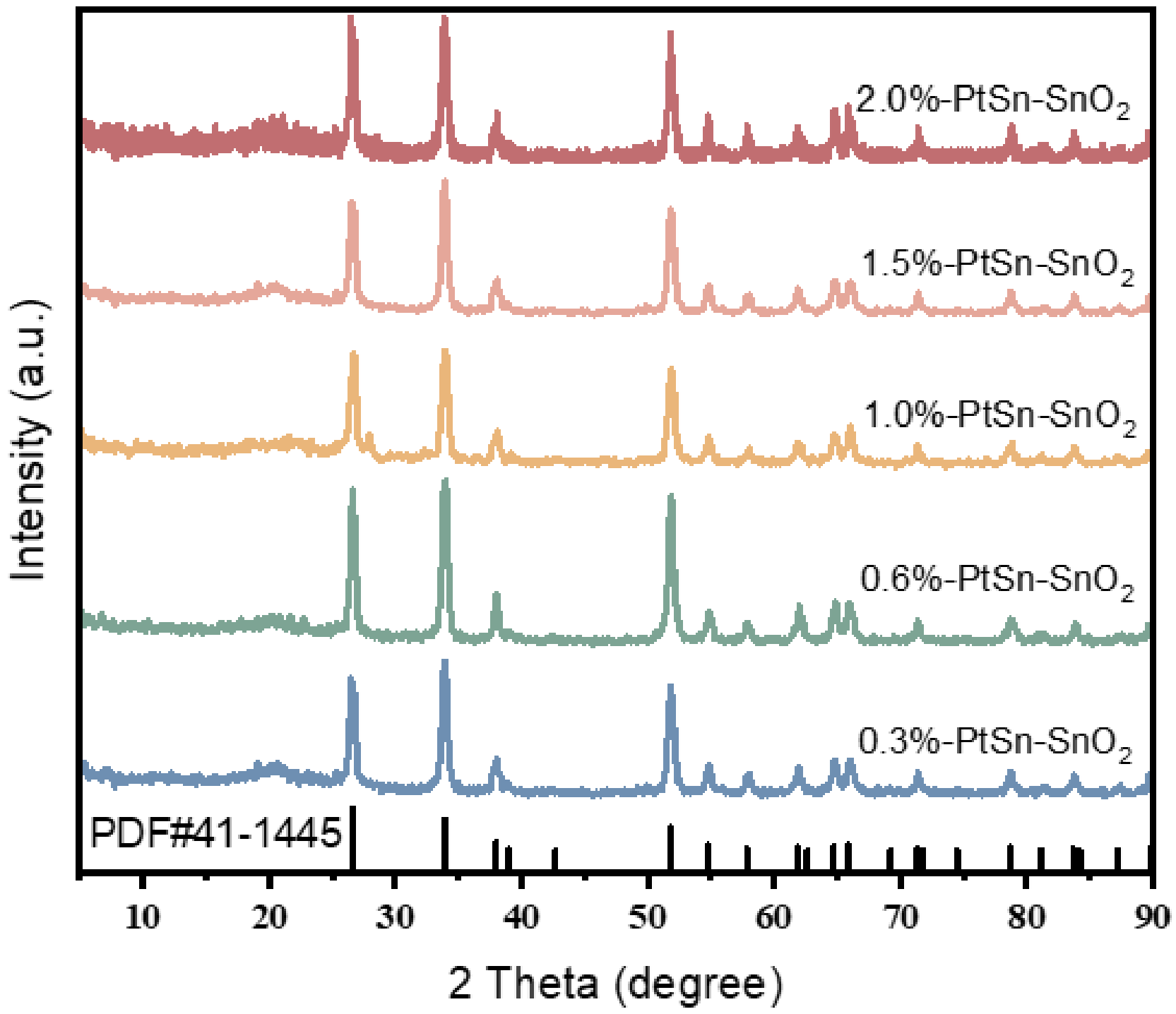

Supplementary Figure 13. XRD spectra of different concentrations of PtSn-SnO_2.

All diffraction peaks in the XRD patterns of these samples can be indexed to the standard PDF #41-1445, indicating the presence of the SnO_2 phase. No characteristic peaks corresponding to the Pt_3Sn alloy or Pt NPs are detected, which could be attributed to the low loading concentration.

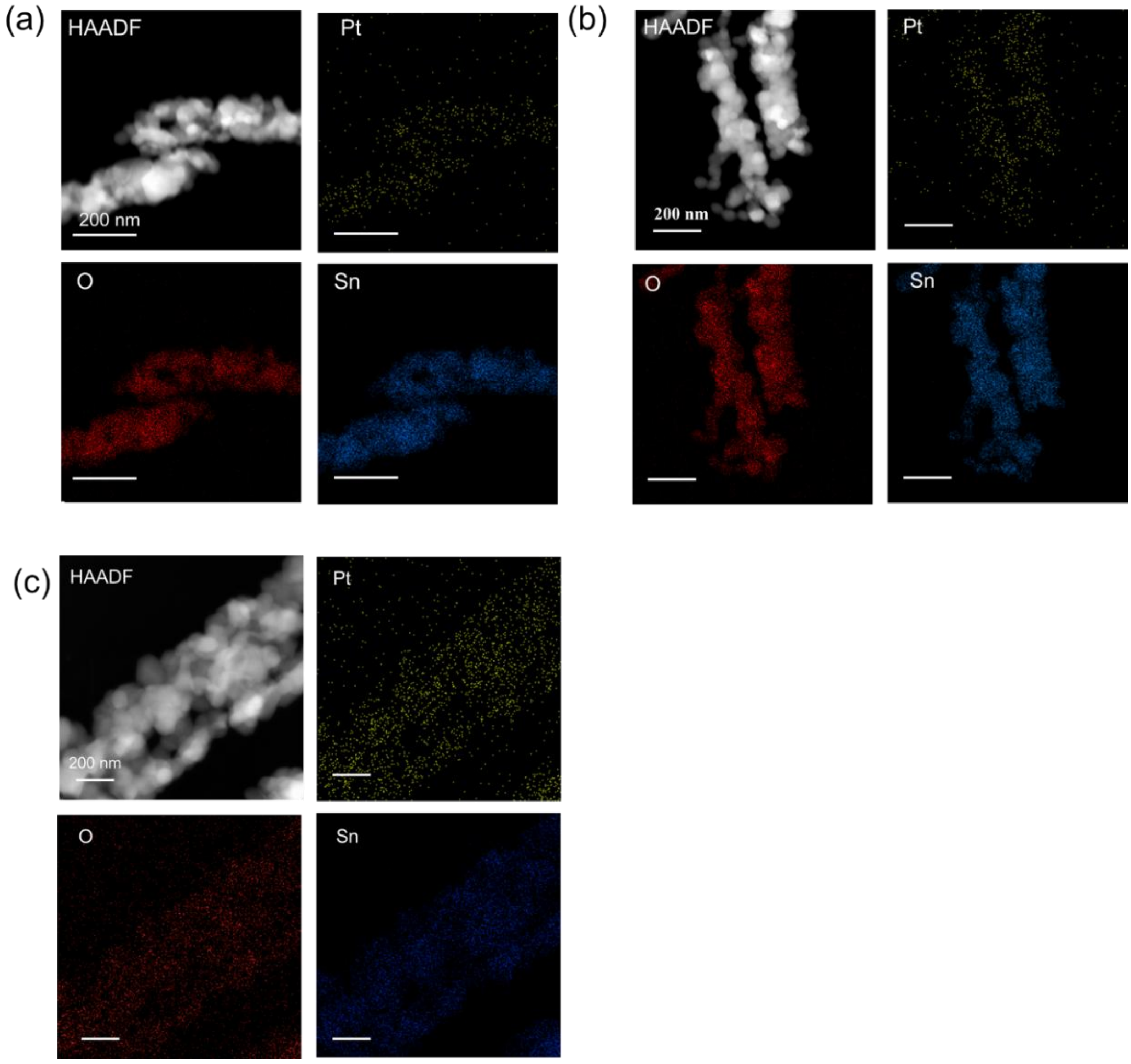

Supplementary Figure 14. EDS-mapping images of (a) 0.3 %-PtSn-SnO_2 and (b) 0.6 %-PtSn-SnO_2 (c) 1.0 %-PtSn-SnO_2.

EDS-mapping results of 0.3 %-PtSn-SnO_2 (Fig.S14(a)), 0.6 %-PtSn-SnO_2 (Fig.S14(b)) and 1.0 %-PtSn-SnO_2 (Fig.S14(c)) samples indicate that no agglomerated Pt particles are observed on the surfaces.

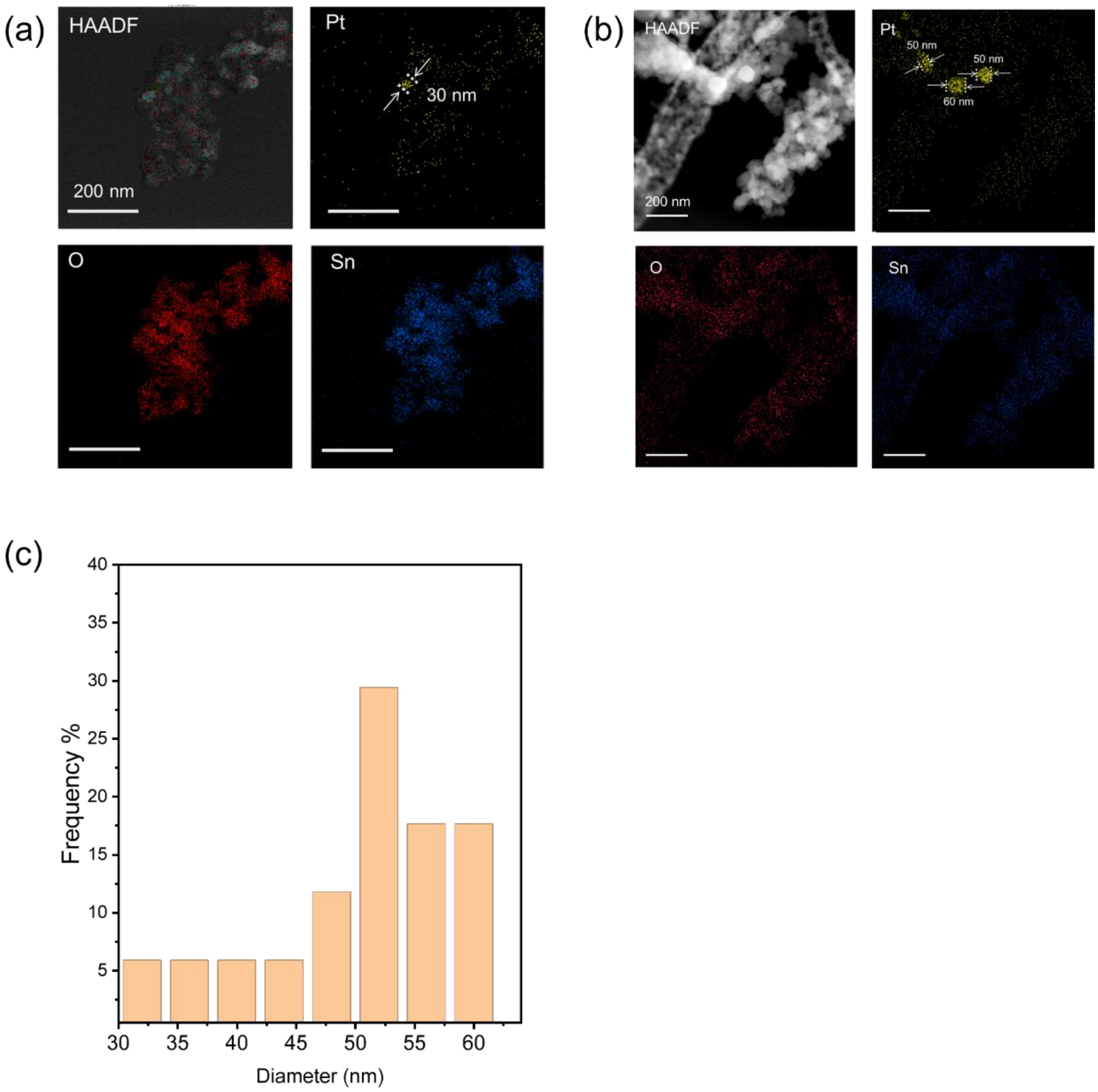

Supplementary Figure 15. EDS-mapping images of (a) 1.5%-PtSn-SnO_2 and (b) 2%-PtSn-SnO_2. (c) Particle size distribution of 1.5%-PtSn-SnO_2 and 2%-PtSn-SnO_2 samples.

EDS-mapping results of 1.5 %-PtSn-SnO_2 (Fig.S15(a)) and 2 %-PtSn-SnO_2 (Fig.S15(b)) show that distinct Pt particles on the surfaces of samples. At lower loadings (≤ 1 wt.%), Pt remains highly dispersed. EDS-mapping results of 1.5%-PtSn-SnO_2 (Fig.S15(a)) and 2%-PtSn-SnO_2 (Fig.S15(b)) show that distinct Pt particles on the surfaces of samples. The particle size distribution histogram of Pt (Fig. S15(c)) indicates that when the loading of the Pt_3Sn alloys exceeds 1.5 wt.%, the Pt species aggregate to form Pt nanoparticles with diameters approximately ranging from 30 to 60 nm.

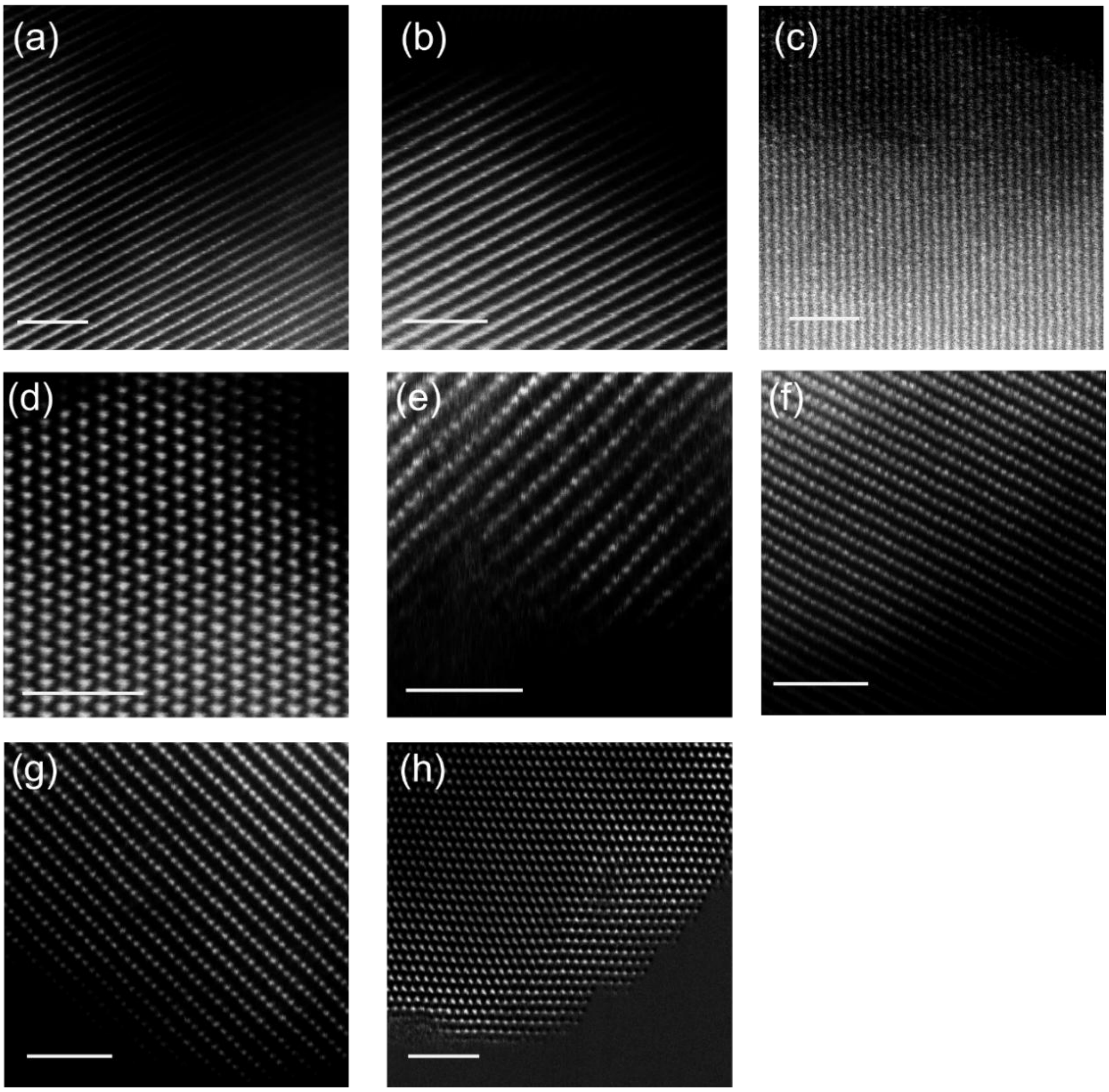

Supplementary Figure 16. Original HAADF-STEM images of 1%-PtSn-SnO_2 in Fig. 3a (the scale bars are 2 nm).

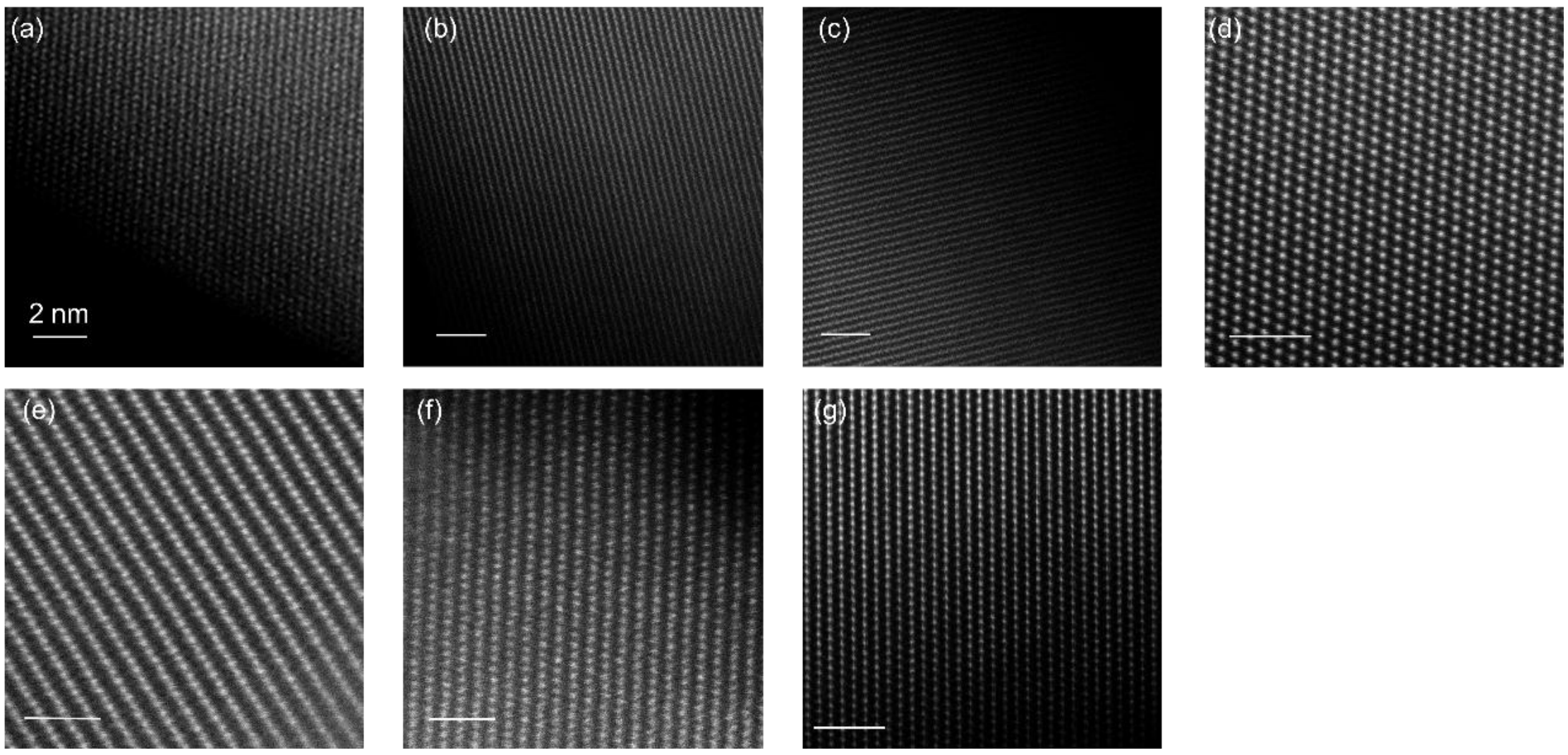

Supplementary Figure 17. Representative HAADF-STEM images of 1%-PtSn-SnO_2 (the scale bars are 2 nm).

As shown in Fig. S17(a-f), monodispersed Pt species are present on the surface of supports, whereas no distinct Pt species are observed in Fig. S17(g).

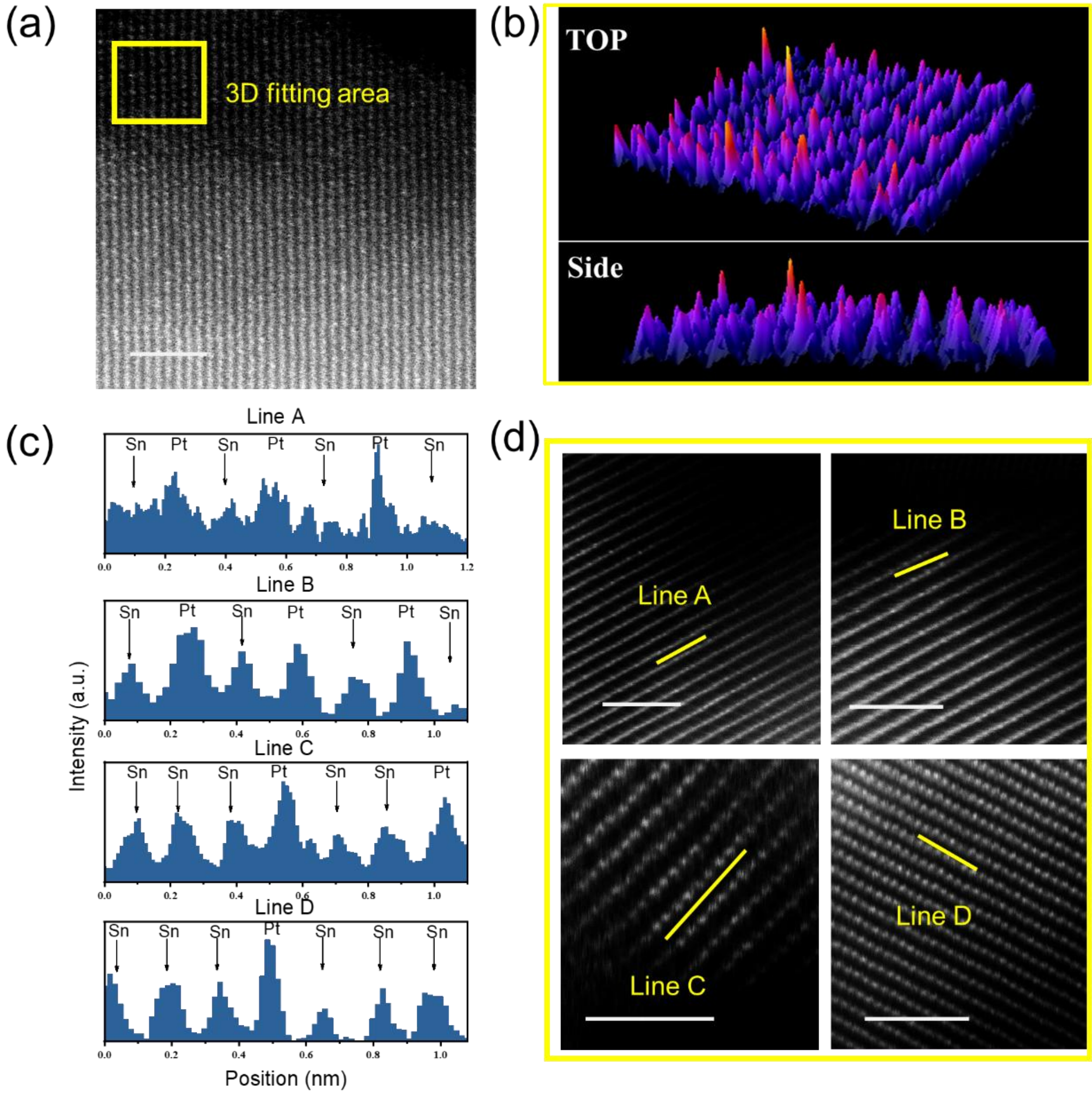

Supplementary Figure 18. (a) HAADF-STEM image of 1%-PtSn-SnO_2. (b) The atom-overlapping Gaussian-function fitting images of selective area scan (measured from white frame of (a)). (c) The intensity profiles obtained in regions X-Y in (d). (d) Other four representative HAADF images of 1%-PtSn-SnO_2.

The HAADF-STEM images in Fig. S18(a, d) cleanly show that the individual Pt atoms atomically disperse over the whole support surface without agglomerate, which is further confirms by the Gaussian-function fitting images (Fig. S18(b)) and the intensity profiles (Fig. S18(c)).

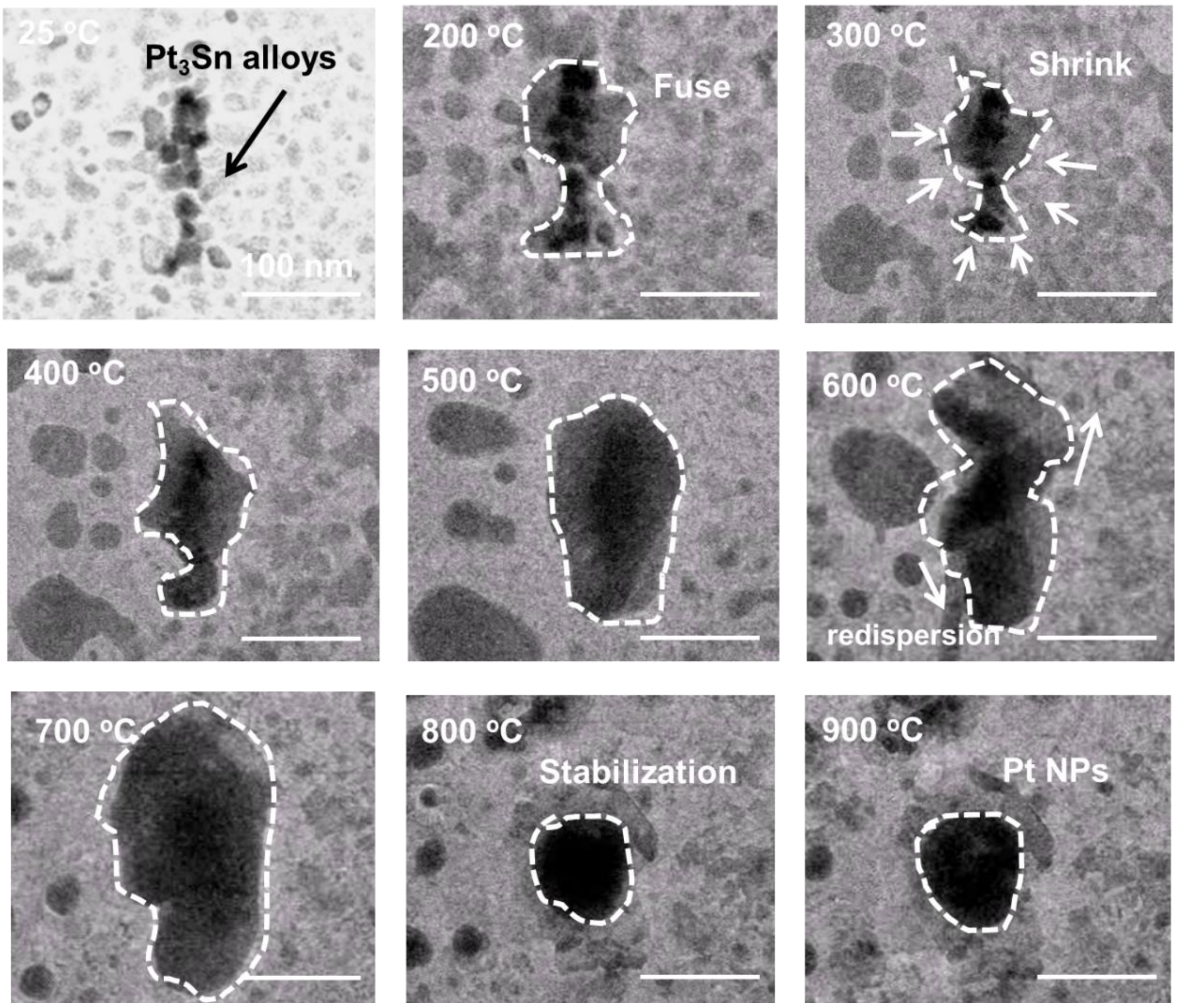

Supplementary Figure 19. In-situ TEM images of the pyrolysis process of 1.5%-PtSn-Sn NFs.

As the rising temperature, the Pt_3Sn alloys first coalesce with the Sn precursor. This is followed by the gradual flow and redispersion of the fusion-body, which evolves into a dark spherical Pt particle.

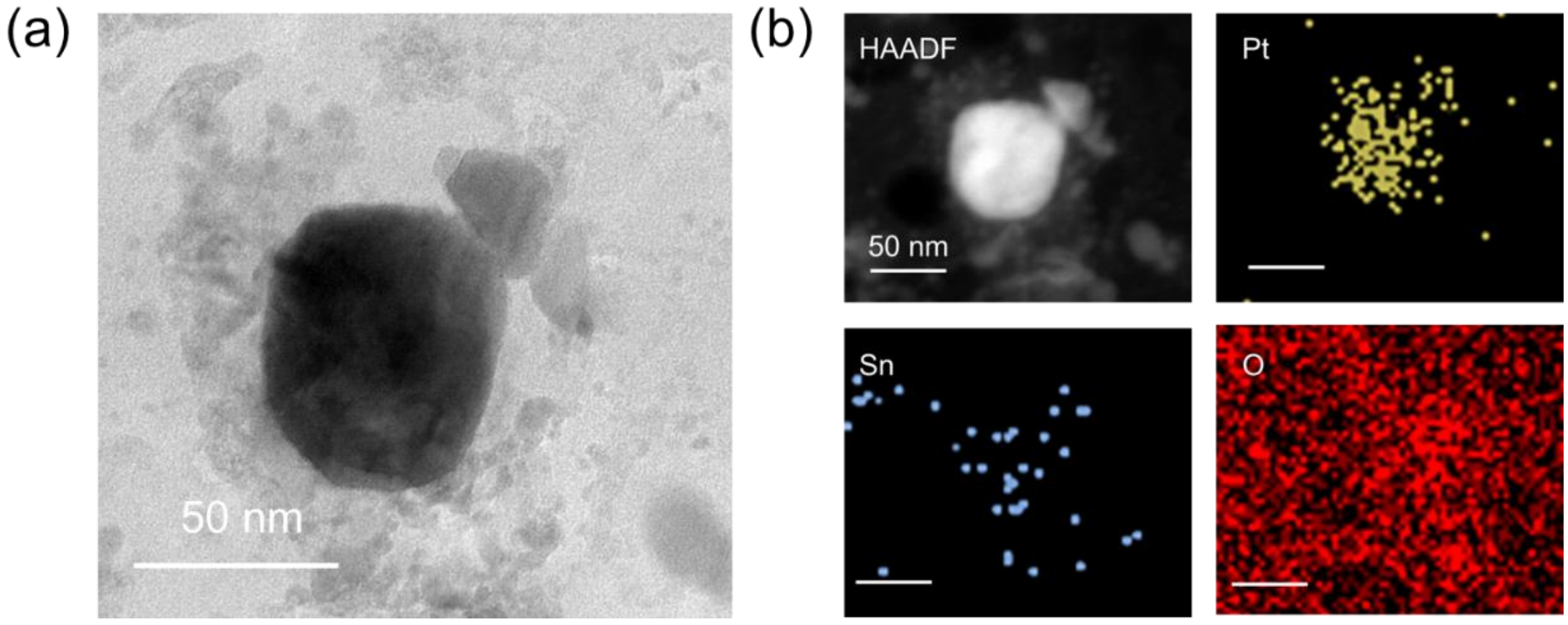

Supplementary Figure 20. (a) TEM image of 1.5%-PtSn-SnO_2 (b) EDS-mapping of 1.5%-PtSn-SnO_2.

As seen in Fig. S20, the Pt species on the surface aggregate into Pt NPs.

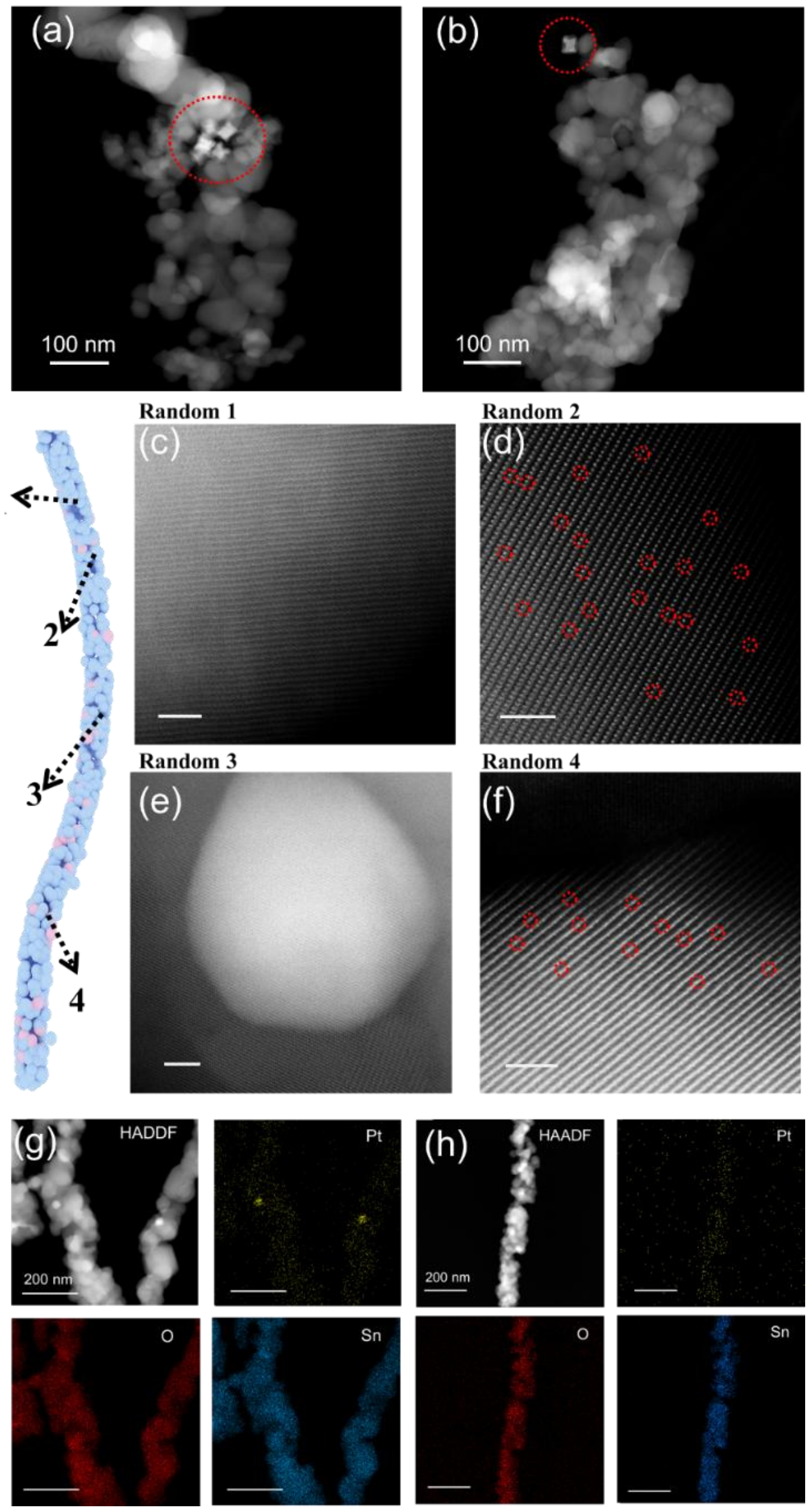

Supplementary Figure 21. (a, b) HAADF images of outer-1%-Pt_3Sn-SnO_2. (c-f) HAADF-STEM images of different areas of outer-1%-PtSn-SnO_2. (g, h) EDS-mapping of two areas of outer-1%-PtSn-SnO_2.

The HAADF images in Fig. S21 (a, b) show that cubic Pt_3Sn alloys were mixed and dispersed within the outer-1%-Pt_3Sn-SnO_2 sample. The sample after pyrolysis at 900 °C in air is named outer-1%-PtSn-SnO_2. As seen in Fig. S21(c-h), four random regions selected from single nanorods exhibit Pt NPs and single dispersed Pt species.

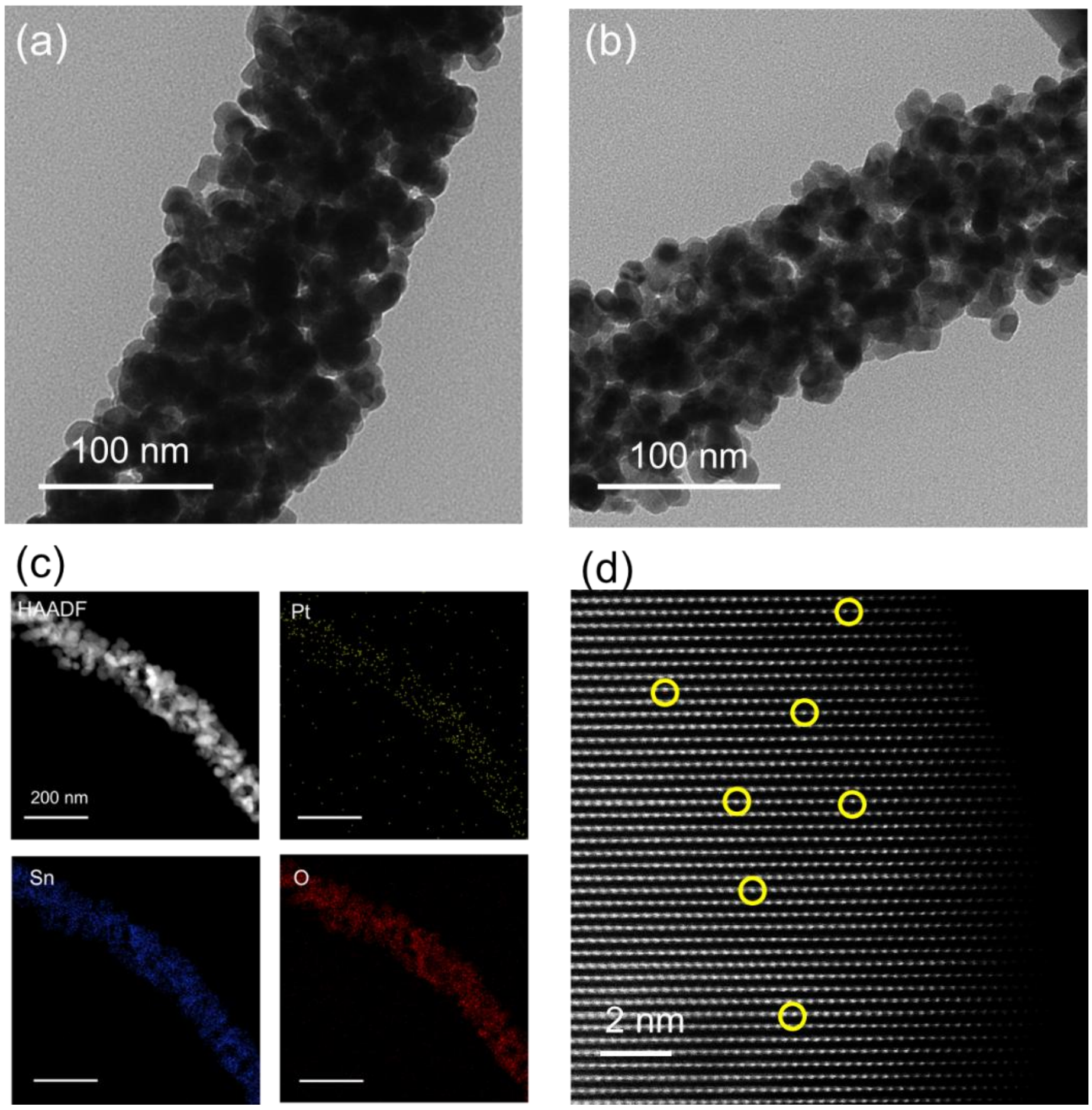

Supplementary Figure 22. (a, b) The TEM images of Pt_1-SnO_2. (c) The EDS elements (Pt, Sn, and O) mapping images of Pt_1-SnO_2. (d) The HADDF-STEM image of Pt_1-SnO_2.

The Pt_1-SnO_2 samples were synthesized via wet-impregnation method. The sample exhibits a nanorod-like morphology with a diameter about 100 nm (Fig. S22(a, b)). As seen in Fig. S22(c), Pt species are uniformly distributed in the SnO_2 surface. The HAADF-STEM images of Pt_1-SnO_2 in Fig. S22(d) shows the presence of Pt atoms on the SnO_2.

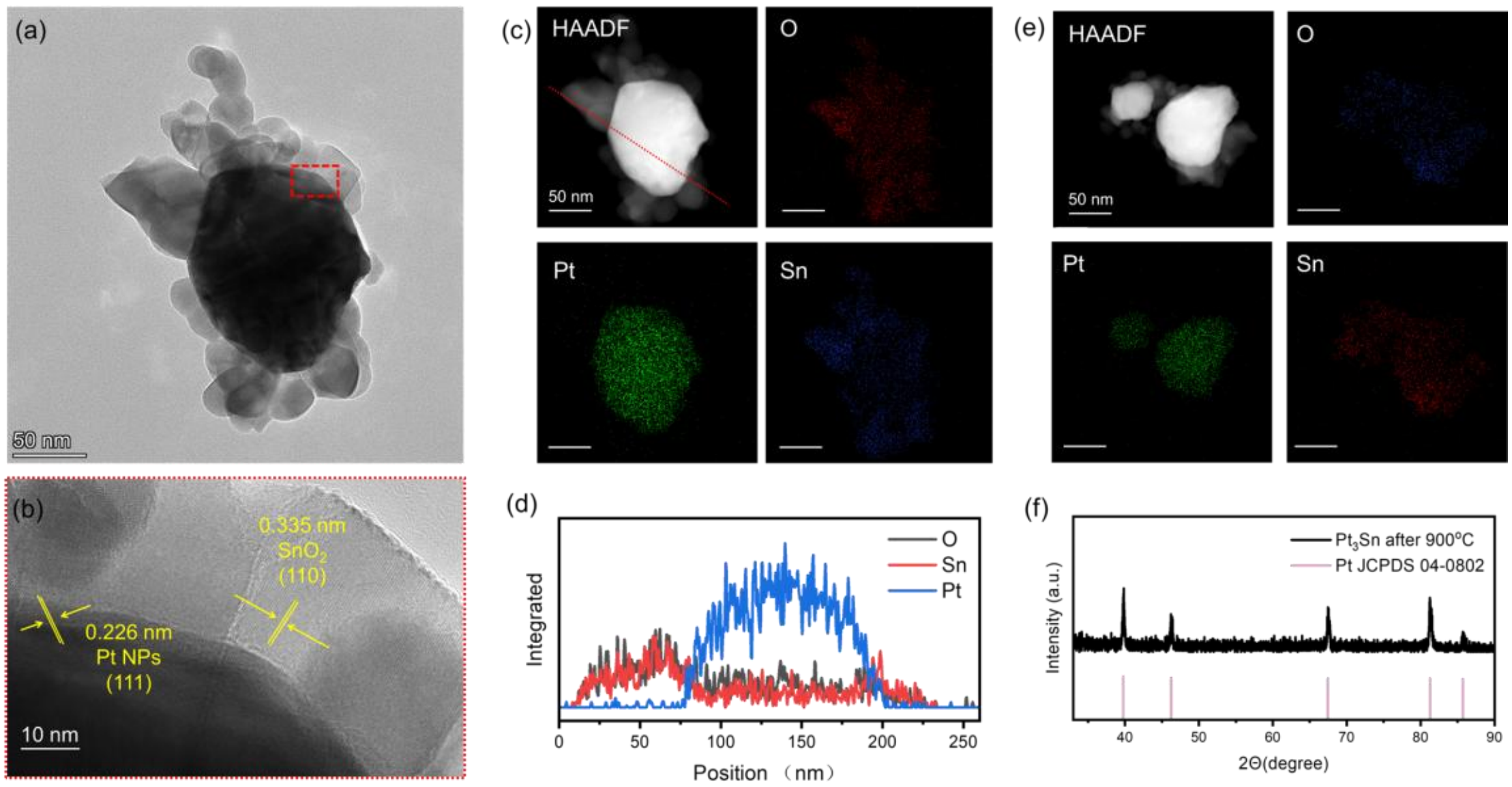

Supplementary Figure 23. (a) TEM image of Pt_3Sn alloys after 900 °C treatment. (b) HRTEM image of the red area in (a). (c, e) EDS-mapping images. (d) The line scan result of the red line in (c). (f) XRD spectra of Pt_3Sn alloys after 900 °C treatment.

Fig. S23(a) shows the morphology of Pt_3Sn alloys after 900 °C treatment. The lattice spacings of 0.226 nm are attributed to the (111) plane of Pt NPs and the lattice spacings of 0.335 nm is attributed to the (110) plane of SnO_2, respectively (Fig. S23(b)). The EDS-mapping and the line scan results (Fig. S23(c, d)) clearly revolve the present of Pt NPs. More respectively EDS-mapping is shown in Fig. S23(e). And the peaks of the samples in the XRD spectra (Fig. S23 (f)) are in good agreement with the standard card (JCPDF:04-0802). Above all, these results indicate that the pyrolysis of the isolated Pt_3Sn alloy yields Pt NPs along with a small amount of SnO_2.

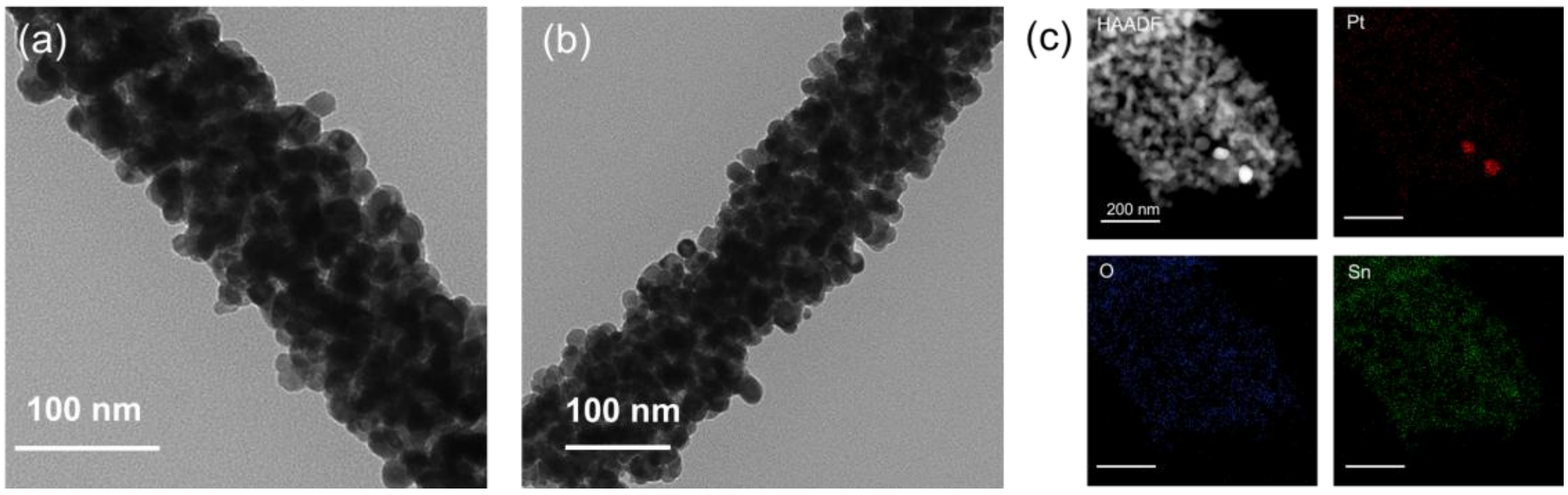

Supplementary Figure 24. (a, b) The TEM images of Pt NPs-SnO_2. (c) The EDS elements (Pt, Sn, and O) mapping images of Pt NPs-SnO_2.

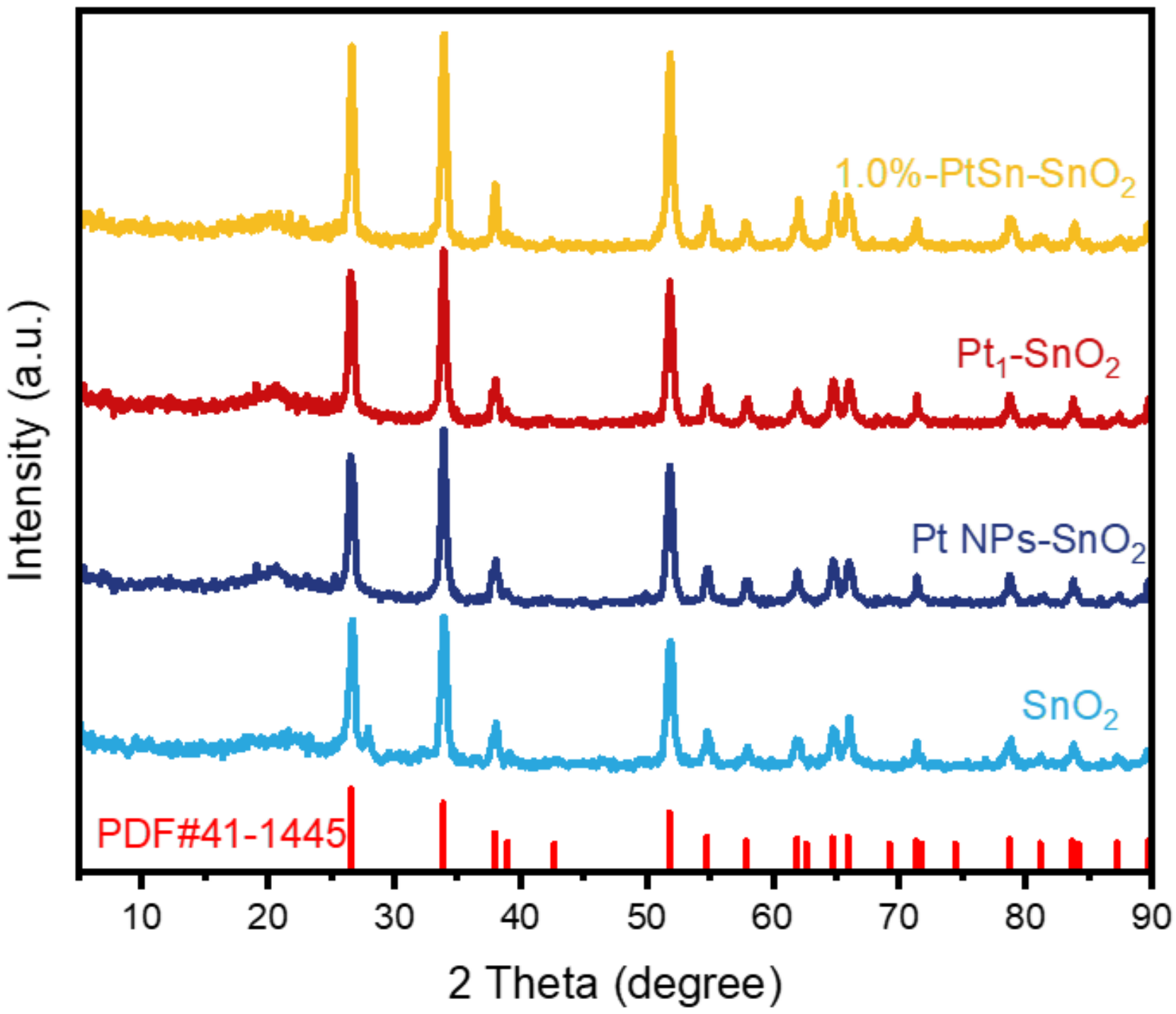

Supplementary Figure 25. XRD spectra of SnO_2, Pt_1- SnO_2, Pt NPs- SnO_2 and 1%-PtSn-SnO_2.

All diffraction peaks in the XRD patterns of these samples can be indexed to the standard PDF #41-1445, indicating the presence of the SnO_2 phase. There are no Pt NPs peaks present in the pattern.

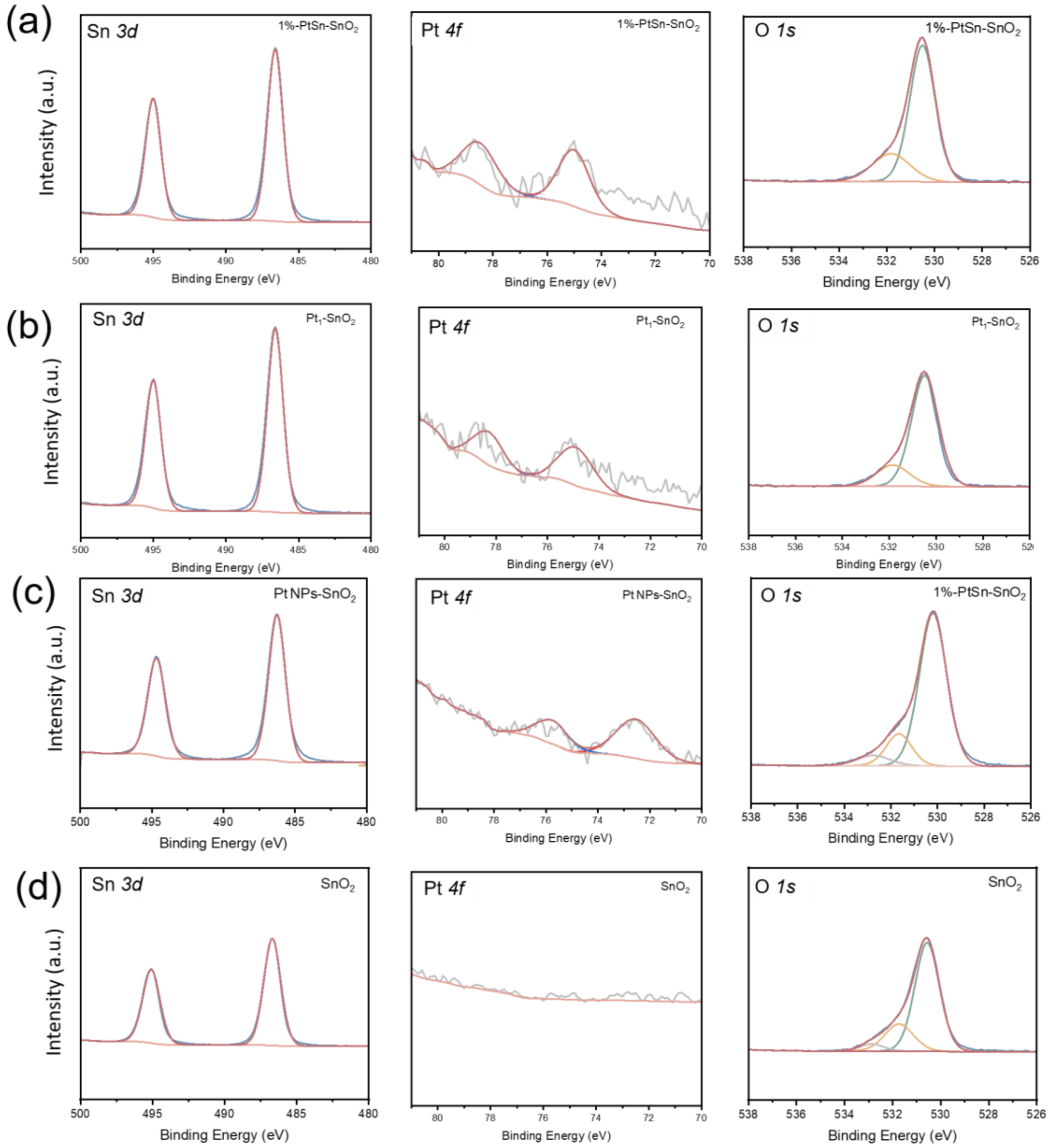

Supplementary Figure 26. Sn 3d, Pt 4f and O1s XPS spectra generated for (a)1%-PtSn-SnO_2, (b)Pt_1-SnO_2, (c)Pt NPs-SnO_2 and (d) SnO_2.

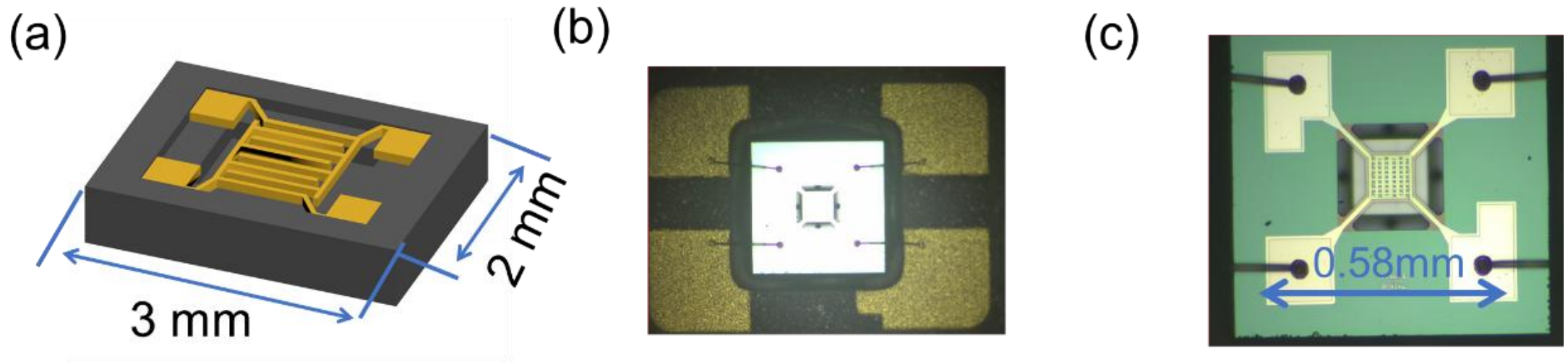

Supplementary Figure 27. (a) The schematic diagram and (b, c) SEM images of the MEMS.

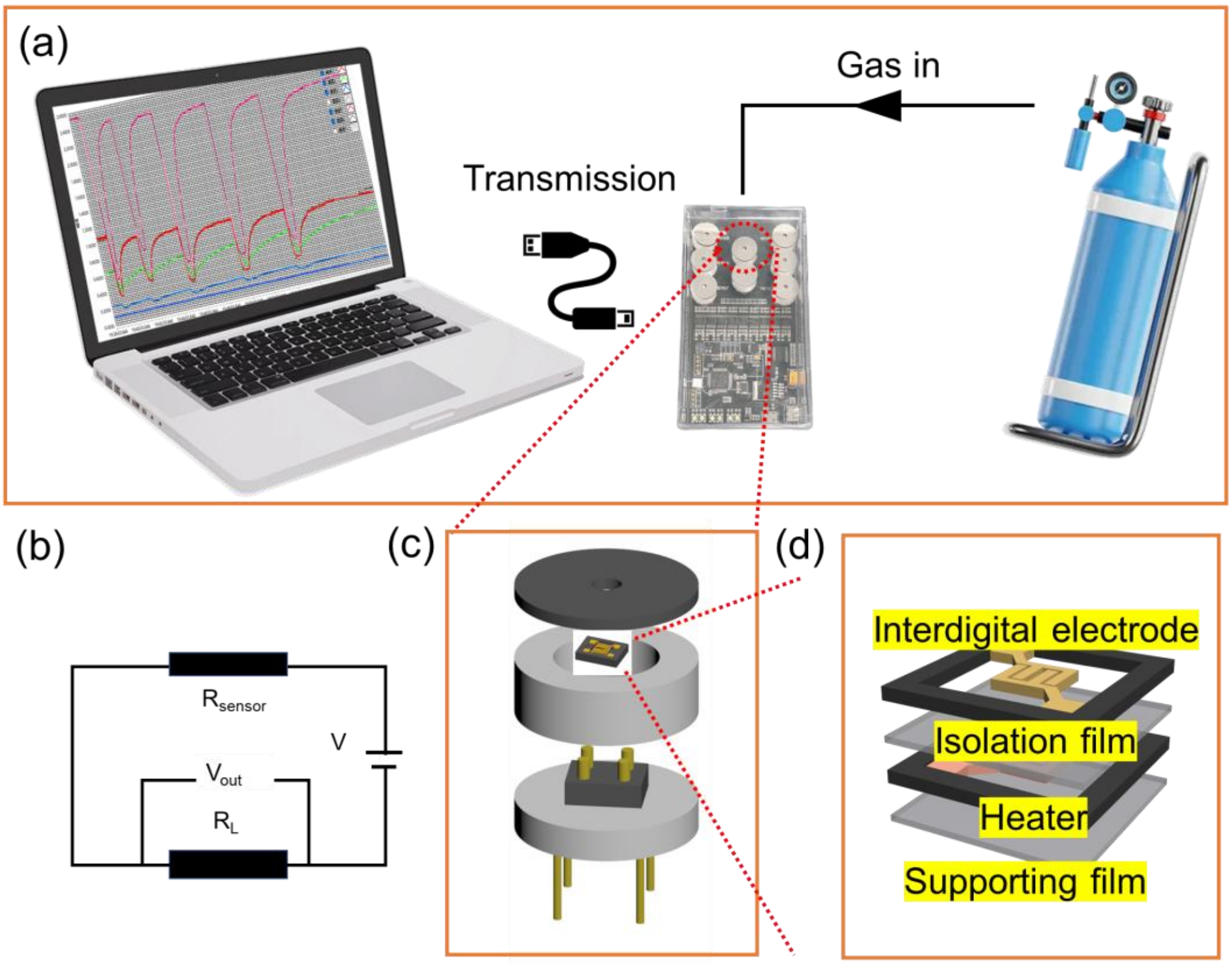

Supplementary Figure 28. Schematic of the fabrication for gas sensing system. (a) the gas sensing system EVM-HP-MC02, (b) measuring circuit of MEMS sensors. (c) the exploded views of test base, (c) MEMS chips.

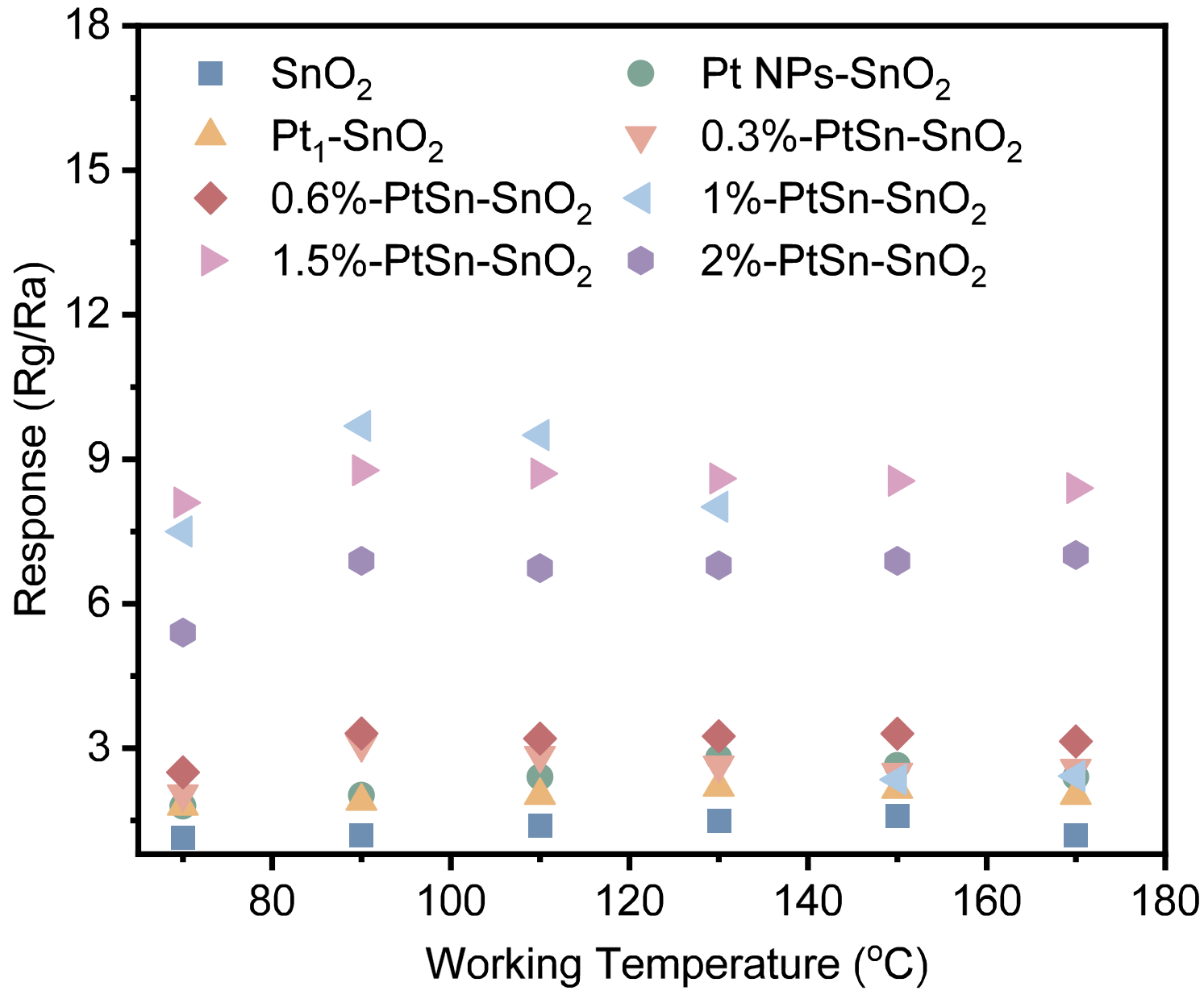

Supplementary Figure 29. Responses of the SnO_2, Pt NPs- SnO_2, Pt_1-SnO_2, 0.3%-PtSn-SnO_2, 0.6%-PtSn-SnO_2, 1%-PtSn-SnO_2, 1.5%-PtSn-SnO_2, 2%-PtSn-SnO_2 sensors to 5 ppm NO_2 at different temperature.

The temperature-dependent response values of all 8 sensors to 5 ppm NO_2 in the temperature ranges of 70 °C to 170 °C are shown in Fig. S29. All the sensors exhibit a trend of increasing and then decreasing response values with a rise in temperature. The gas-sensing reaction in semiconductors is essentially a surface catalytic redox process. At low temperatures, gas molecules lack sufficient activation energy, resulting in sluggish surface reaction kinetics and weak sensing signals. Conversely, at excessively high temperatures, although the chemical reaction rate accelerates, the desorption rate of gas molecules surpasses their adsorption rate, leading to a decline in response. The peak response is achieved only at the optimal working temperature, where adsorption and reaction reach a dynamic equilibrium. Considering the interplay between sensitivity and power consumption, the operating temperature for each sensor was defined as the point of maximum sensitivity. Therefore, the optimum working temperature of SnO_2, Pt NPs-SnO_2, Pt_1-SnO_2, 0.3%-PtSn-SnO_2, 0.6%-PtSn-SnO_2, 1%-PtSn-SnO_2, 1.5%-PtSn-SnO_2, 2%-PtSn-SnO_2 sensors are 150 °C, 130 °C, 130 °C, 90 °C, 90 °C, 90 °C, 90 °C and 90 °C, respectively.

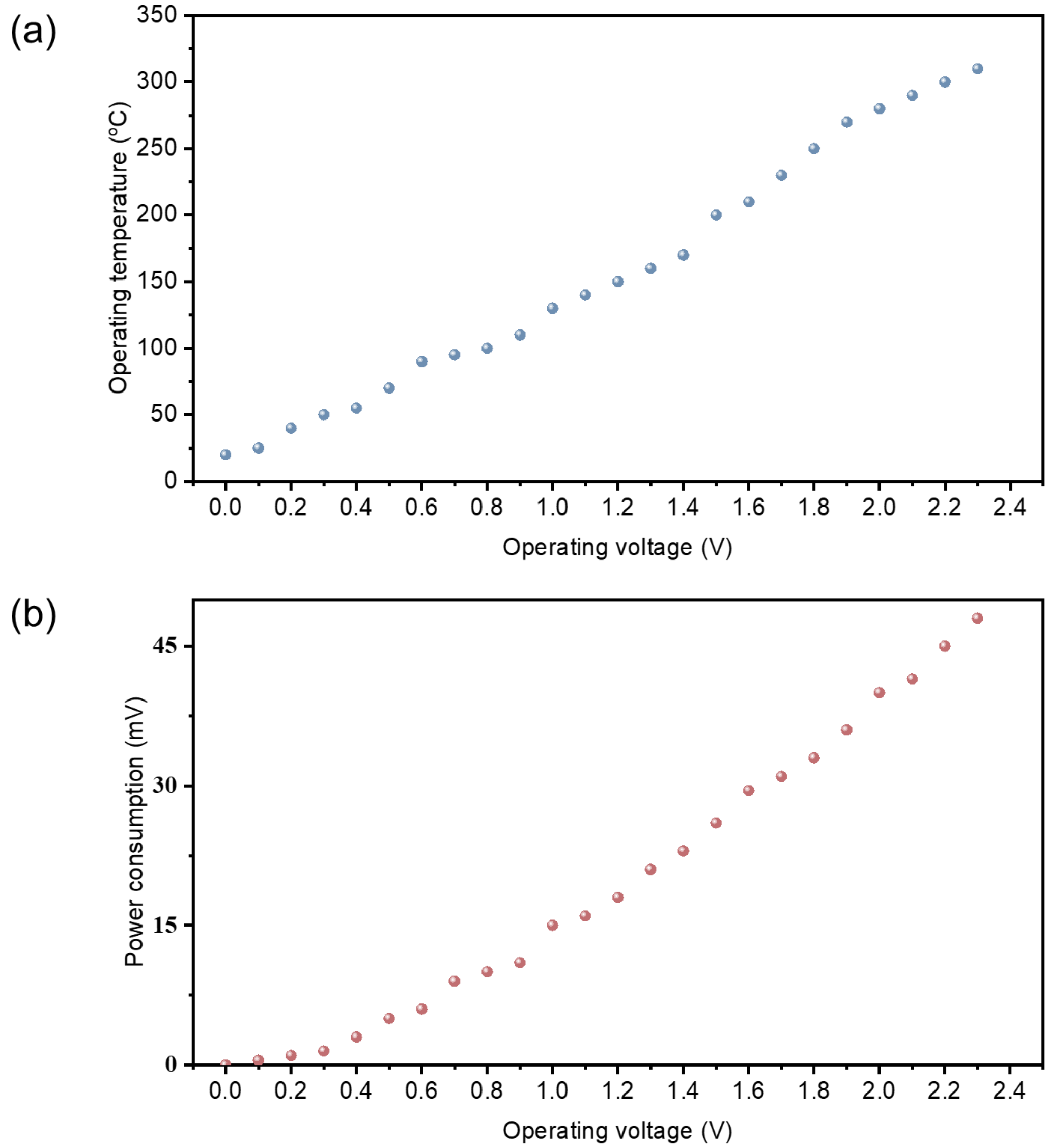

Supplementary Figure 30. (a) The relationship between operating voltage and the working temperature. (b) The relationship between operating voltage and the power consumption of sensors.

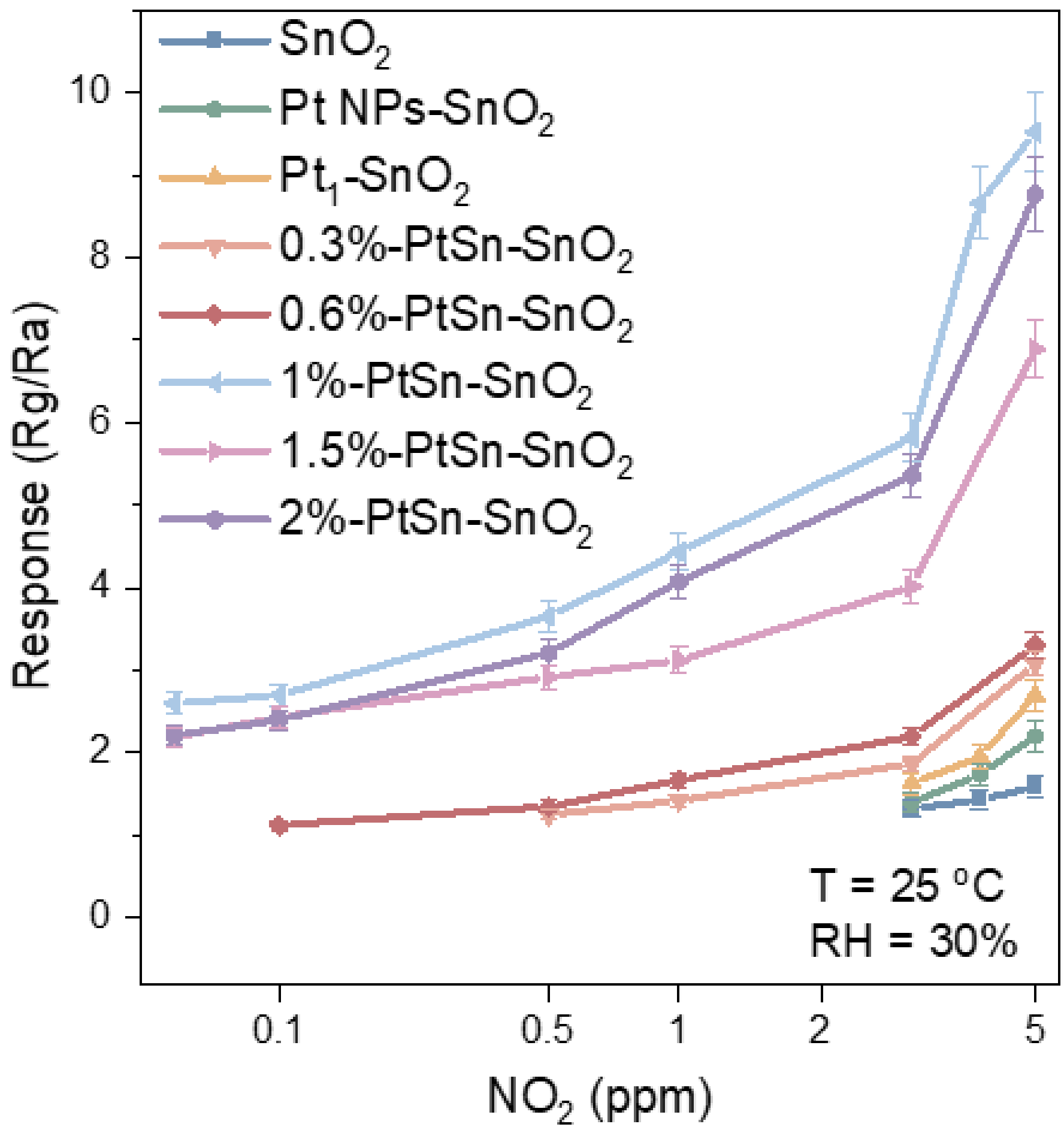

Supplementary Figure 31. Responses of the SnO_2, Pt NPs- SnO_2, Pt_1-SnO_2, 0.3%-PtSn-SnO_2, 0.6%-PtSn-SnO_2, 1%-PtSn-SnO_2, 1.5%-PtSn-SnO_2, 2%-PtSn-SnO_2 sensors towards 0.05-5 ppm NO_2 gas.

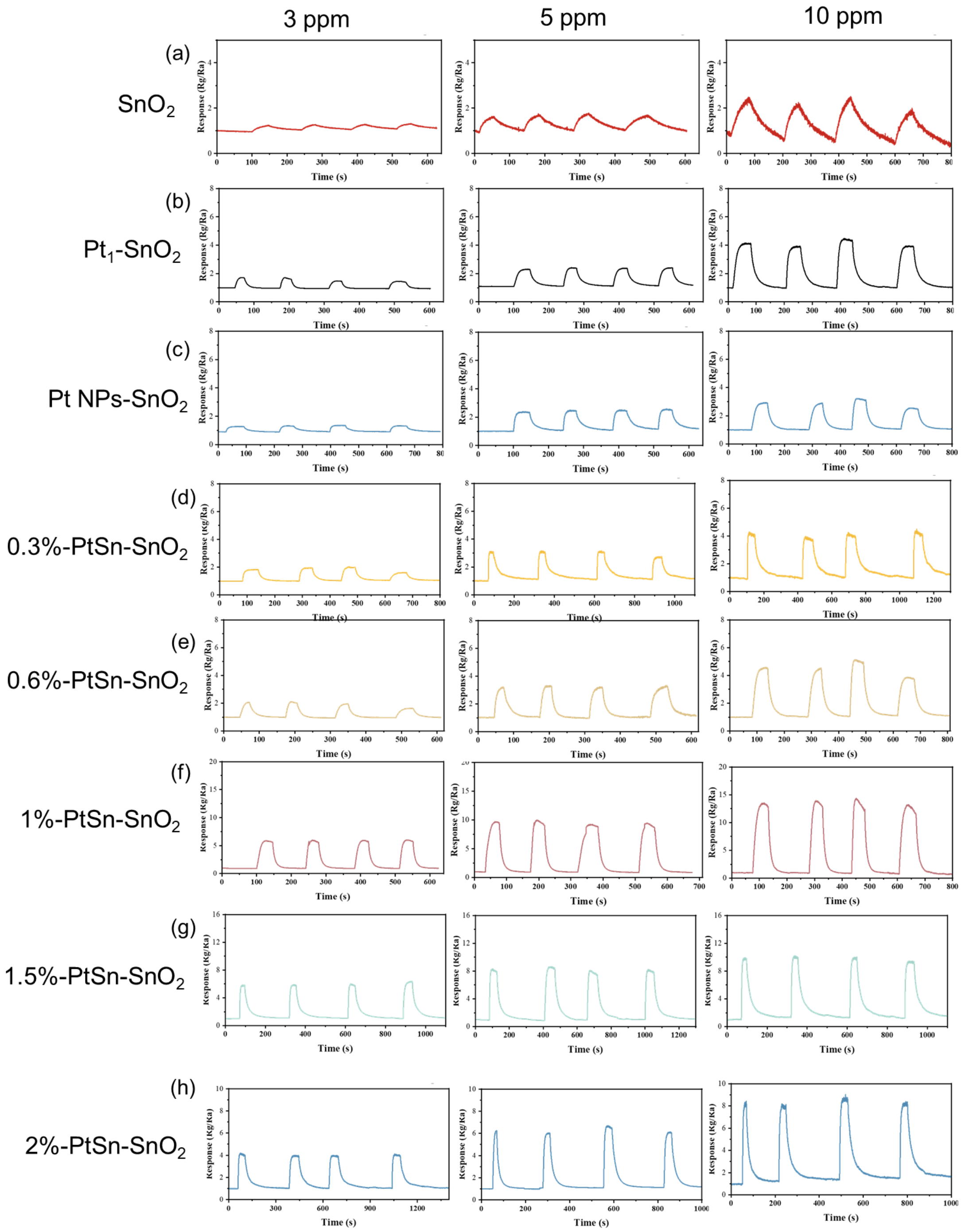

Supplementary Figure 32. Time-related dynamic responses for 3 ppm, 5 ppm and 10 ppm NO_2 of 8 sensors.

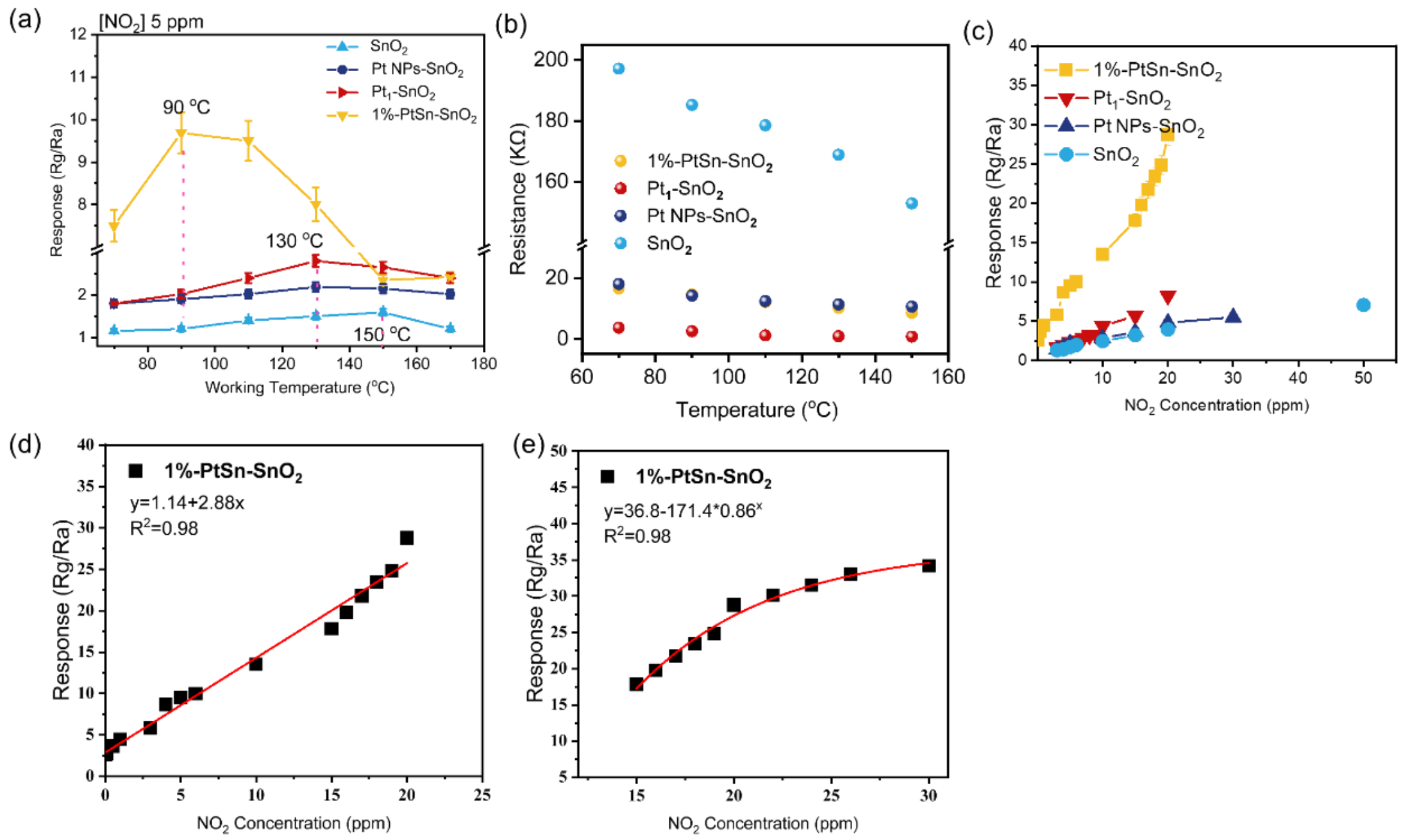

Supplementary Figure 33. (a-c) Gas performances of SnO_2, Pt NPs-SnO_2, Pt_1-SnO_2, 1%-PtSn-SnO_2 sensors. Gas response (d)at low concentration of NO_2 gas (0.05-20 ppm) and (e) high concentration of NO_2 gas (15-30 ppm).

The optimum working temperature of SnO_2, Pt NPs-SnO_2, Pt_1-SnO_2, 1%-PtSn-SnO_2 sensors are firstly evaluated. Fig. S33(a) reveals that the optimum working temperatures of SnO_2, Pt NPs-SnO_2, Pt_1-SnO_2, 1%-PtSn-SnO_2 sensors are 150 ºC, 130 ºC, 130 ºC and 90 ºC, respectively. The temperature-dependent resistances of all sensors are shown in Fig. S33(b). The resistance values decrease with the operating temperature increasing, exhibiting typical semiconductor characteristics. The response values of four sensors to different NO_2 concentrations are shown in Fig. S33(c). The 1%-PtSn-SnO_2 sensors exhibit the highest response value to NO_2. For the 1%-PtSn-SnO_2 sensors, the sensor responses exhibit a linear relationship with NO_2 concentration in the low-concentration regime (Fig. S33(e)). However, as the NO_2 concentration increases beyond the point where the available adsorption sites on the material surface become saturated, the responses transition to a nonlinear dependence on NO_2 concentrations (Fig. S33(f)).

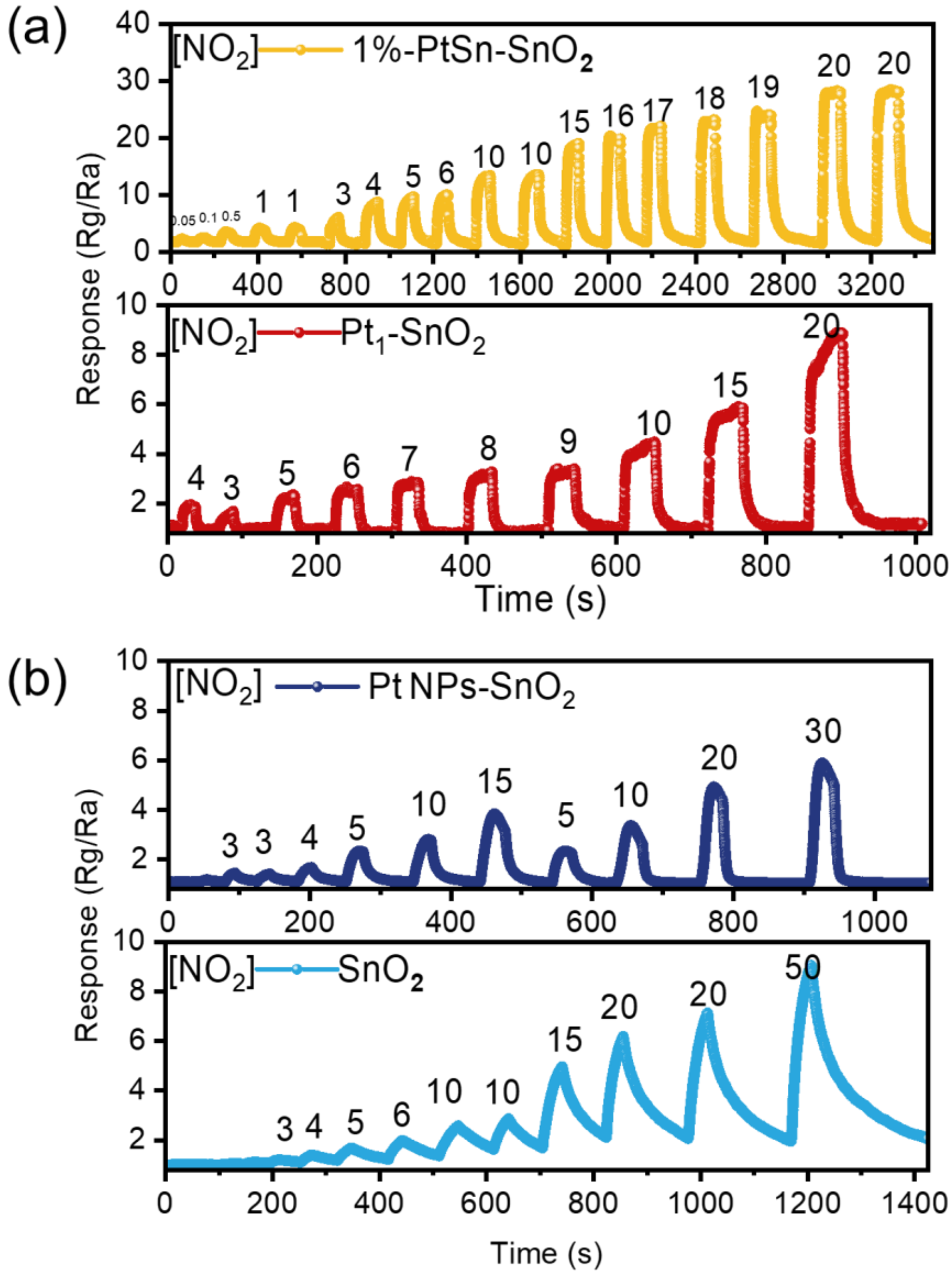

Supplementary Figure 34. Time-related dynamic responses of (a) 1%-PtSn-SnO_2 and Pt_1-SnO_2 and (b) Pt NPs-SnO_2 and SnO_2.

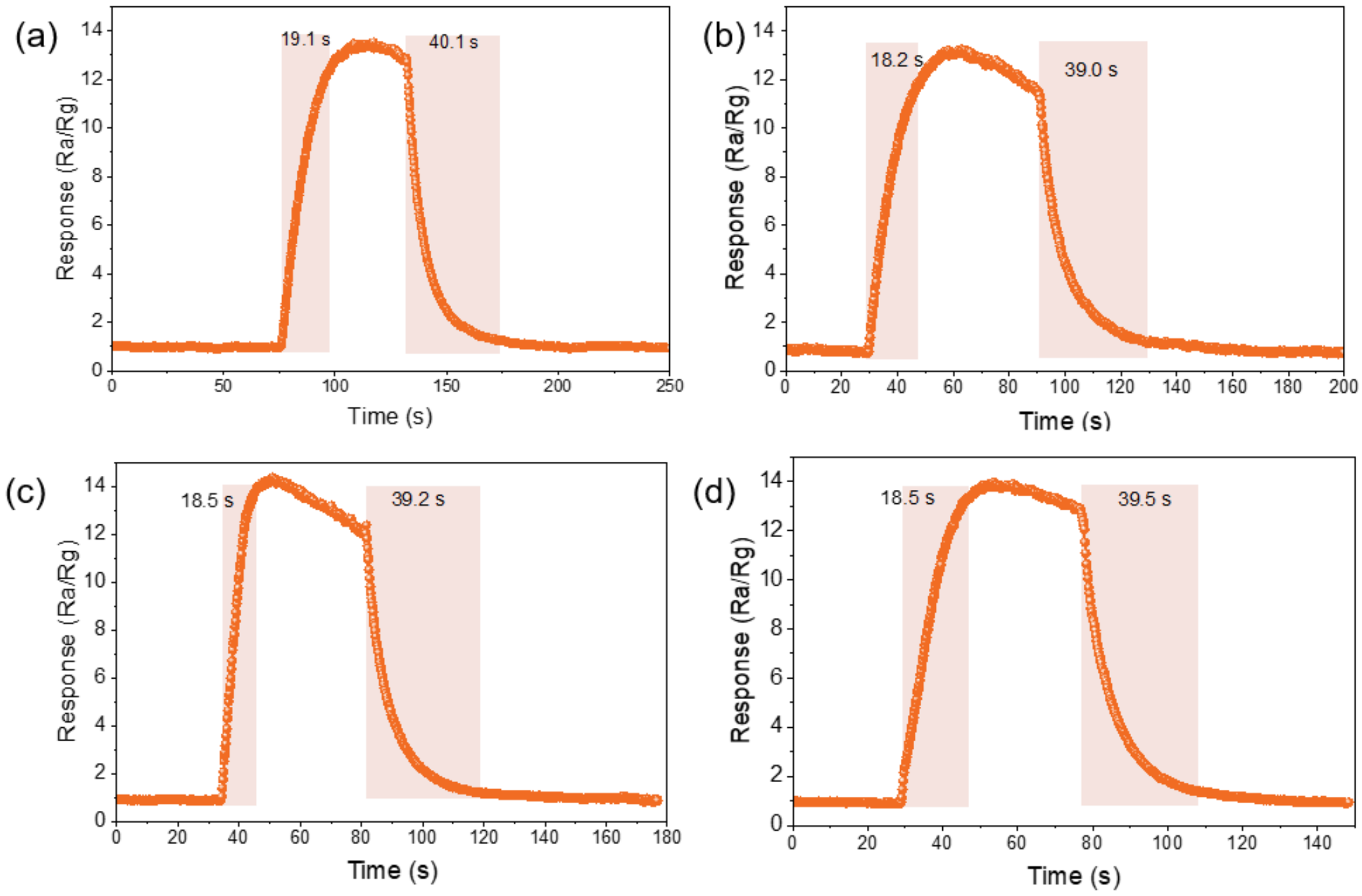

Supplementary Figure 35. Time-related dynamic responses of 1%-PtSn-SnO_2 to 10 ppm NO_2.

The average response time of 1%-PtSn-SnO_2 sensors to 10 ppm NO_2 is 18.6s and the average recover time is 39.4s.

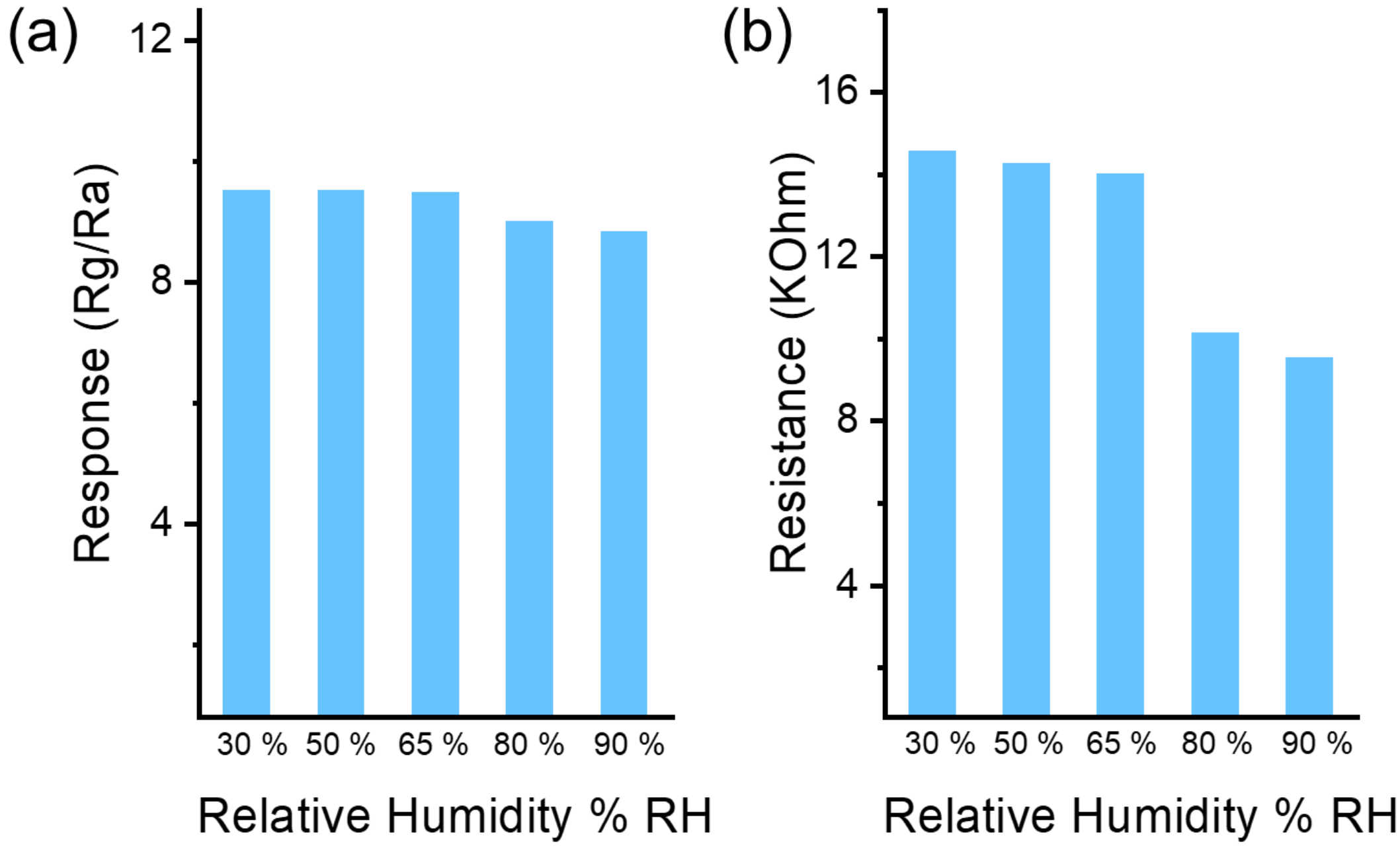

Supplementary Figure 36. The influence of humidity to the 1%-PtSn-SnO_2 sensors on (a) the response value to the NO_2 (5 ppm) and (b) the resistance of the 1%-PtSn-SnO_2.

Considering the possibility of changes in environmental humidity, the impact of humidity on sensor performance was assessed. Sensor humidity tests were conducted at an ambient temperature of 25 °C. The response values change (Fig. S36(a)) and resistances change (Fig. S36(b)) of 1%-PtSn-SnO_2 sensors in the 30%-90% RH were tested. It is observed that the resistance value of 1%-PtSn-SnO_2 sensor slightly decreases as increased RH, which is caused by the water molecules adsorption on SnO_2 surface. And the response vales of 1%-PtSn-SnO_2 sensors exhibit a minimal decrease of only 5% under a high relative humidity (RH) condition of 90%, indicating the excellent moisture resistance of 1%-PtSn-SnO_2 sensors.

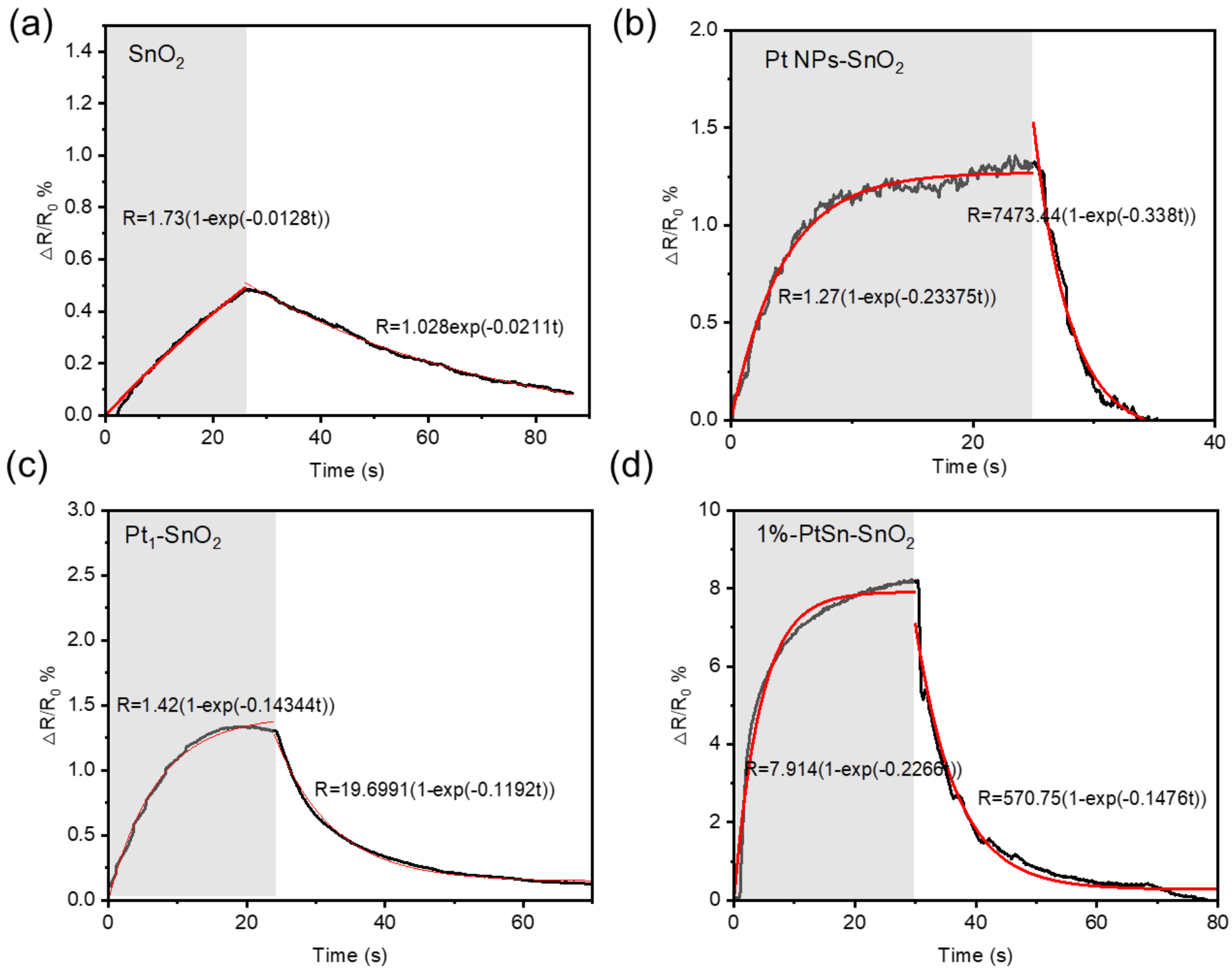

Supplementary Figure 37. Dynamic response transitions and the corresponding response/recovery fitting curves of the sensors.

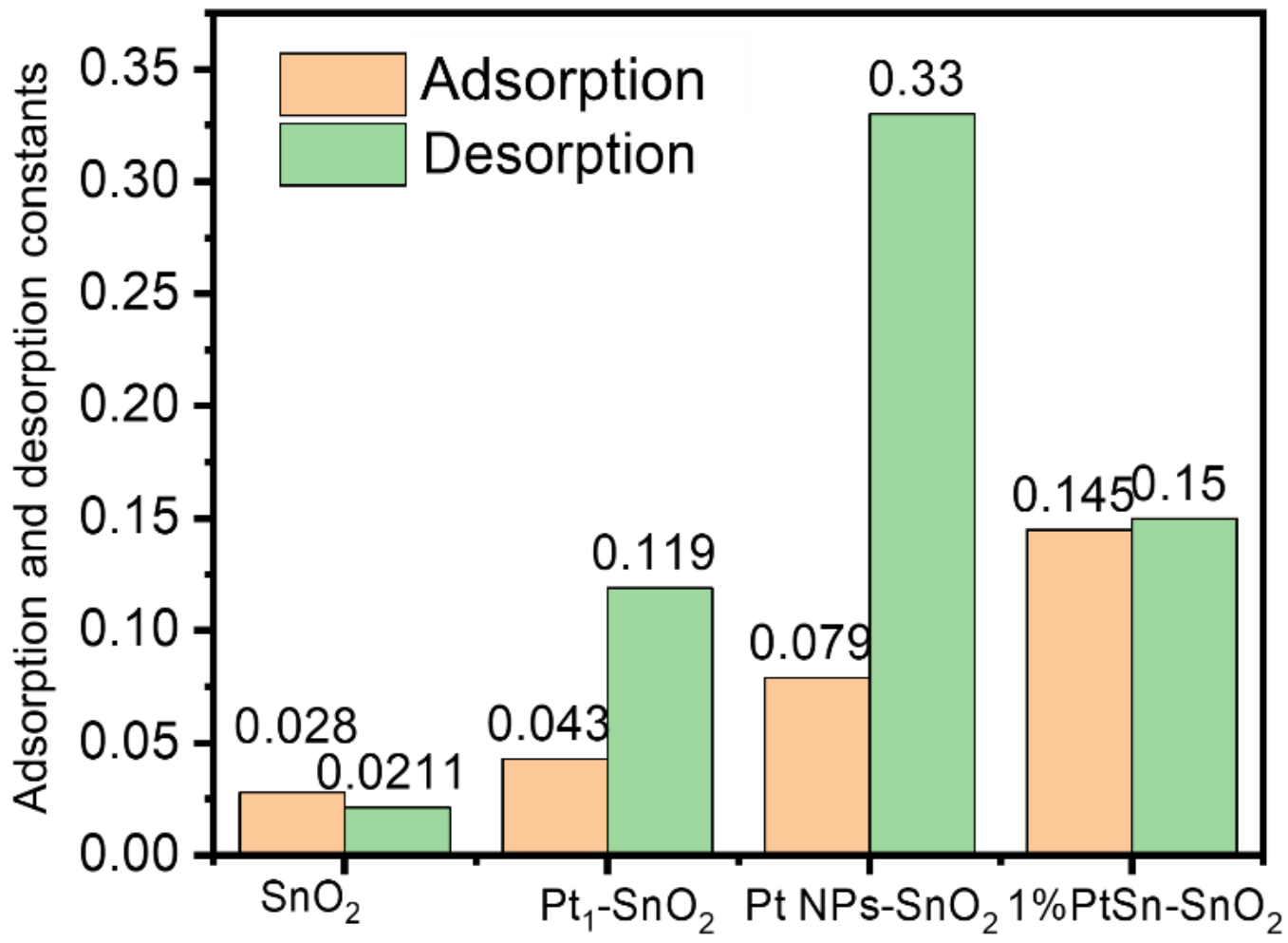

Supplementary Figure 38. Adsorption and desorption rate constant K_{ads} and K_{des} values.

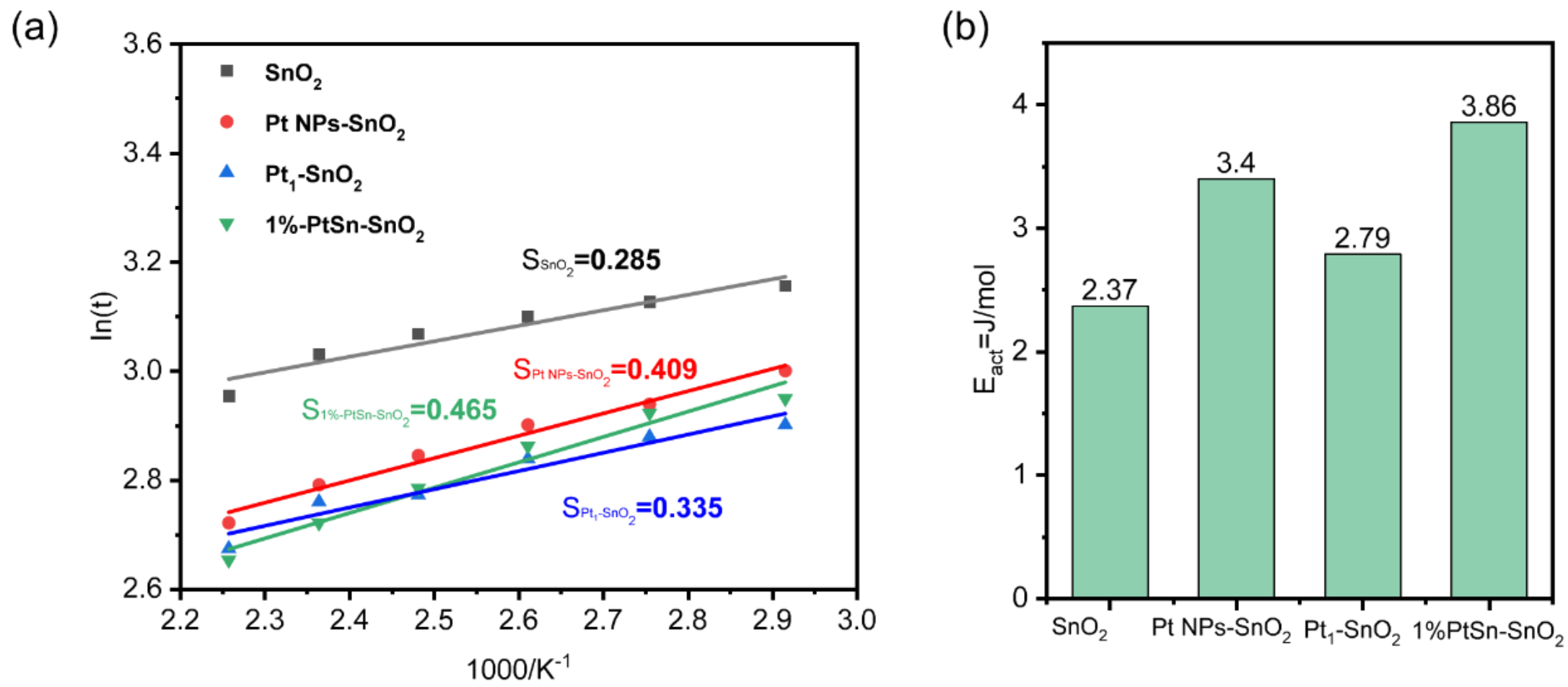

Supplementary Figure 39. (a) Single dynamic curves of response time to 10 ppm NO_2 (b) E_{act} of four sensors.

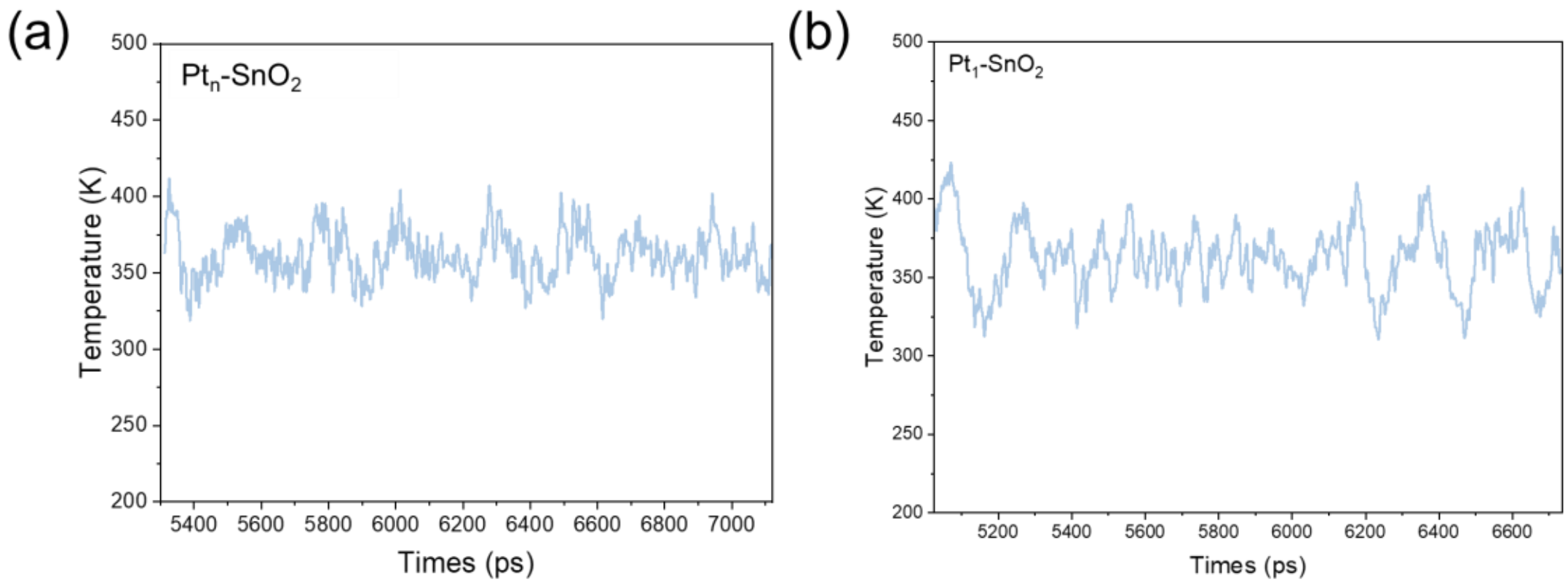

Supplementary Figure 40. A representative example of temperature during AIMD simulation of (a)Pt_n-SnO_2 and (b)Pt_1-SnO_2.

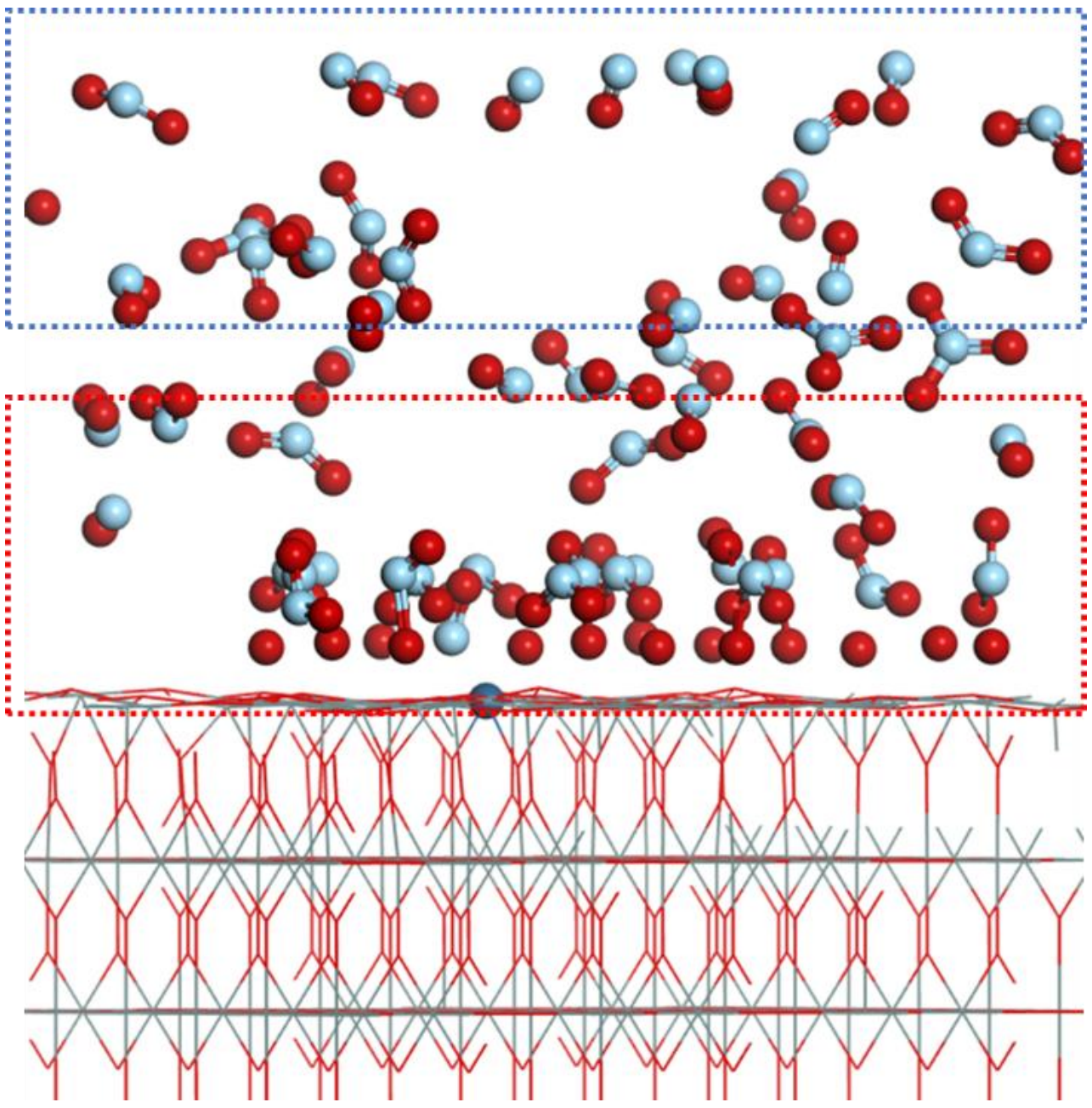

Supplementary Figure 41. Representative snapshot of the Pt_1-SnO_2 for NO_2 molecules.

Due to the periodic boundary conditions of the structure, in a typical structure shown in Fig. S41, the upper part (the blue dashed box) can be equivalently regarded as the adsorption of SnO_2 surface to NO_2 molecules. Compared with the adsorption of Pt_1-SnO_2 surface to NO_2 molecules (the red dashed box), the numbers of NO_2 molecules adsorbed on the surface of the SnO_2 surface are significantly decreased.

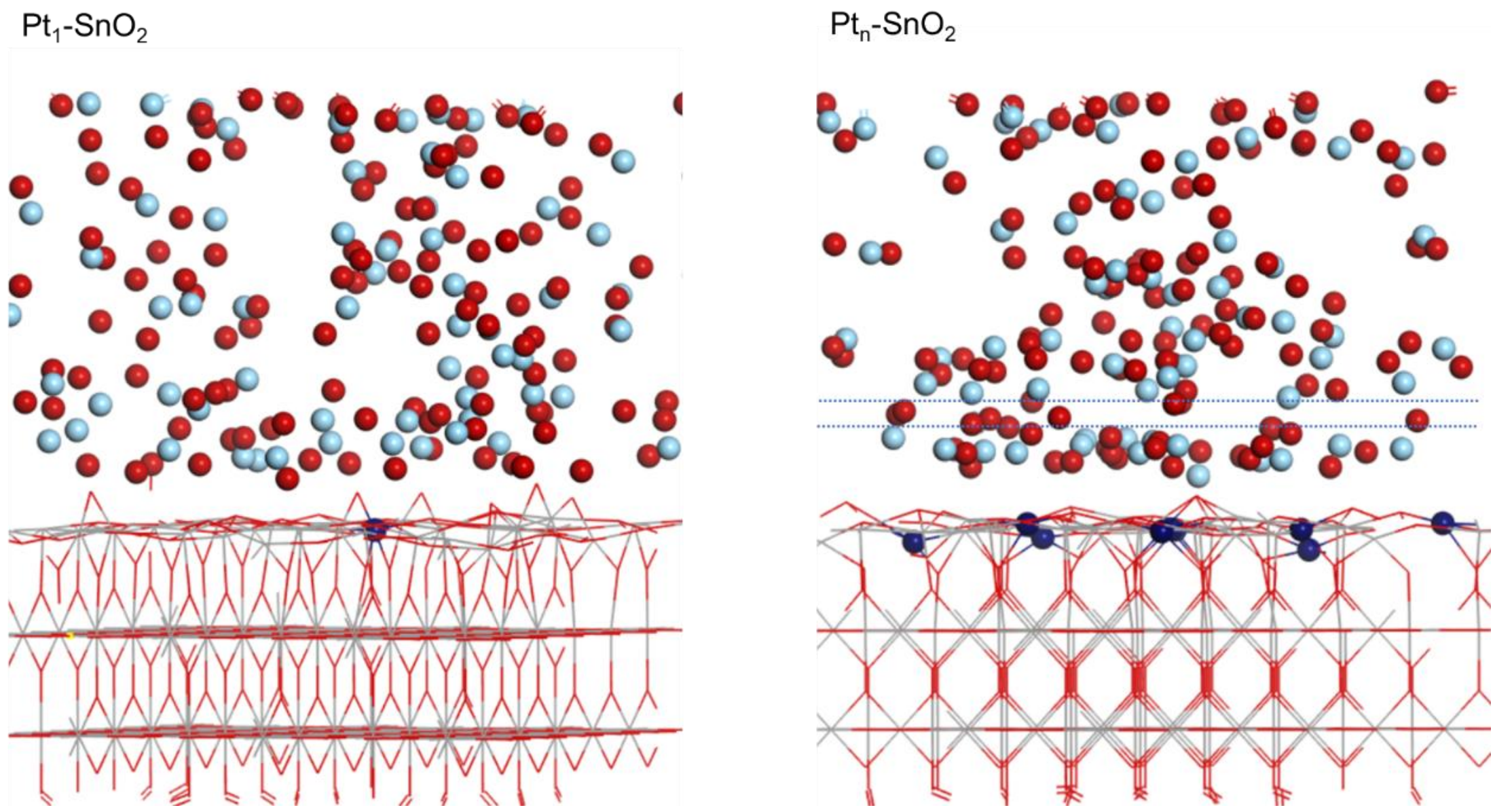

Supplementary Figure 42. Representative snapshots of the interfacial structures for NO_2 molecules over Pt_1-SnO_2 (left) and Pt_n-SnO_2 (right) surface.

As seen in Fig. S42, the distribution pattern of NO_2 molecules shows significant differences on the surfaces of the two different structures. On the surfaces of Pt_n-SnO_2, the NO_2 molecules show an obviously disconnected distribution, while on the surface of Pt_1-SnO_2, the NO_2 molecules present a continuous distribution pattern.

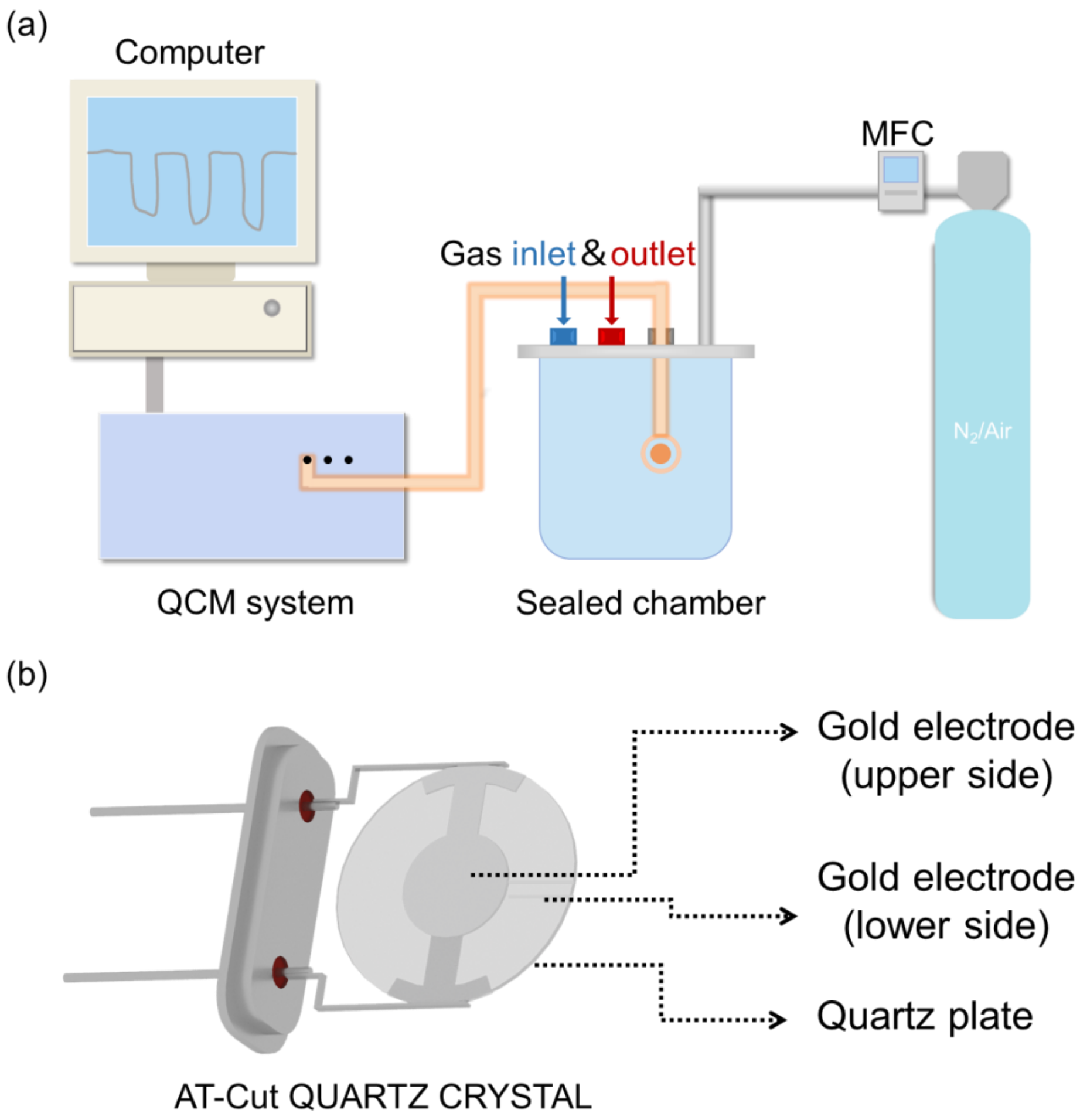

Supplementary Figure 43. (a) Schematic diagram of QCM test equipment. (b) Schematic diagram of quartz crystal using in QCM tests.

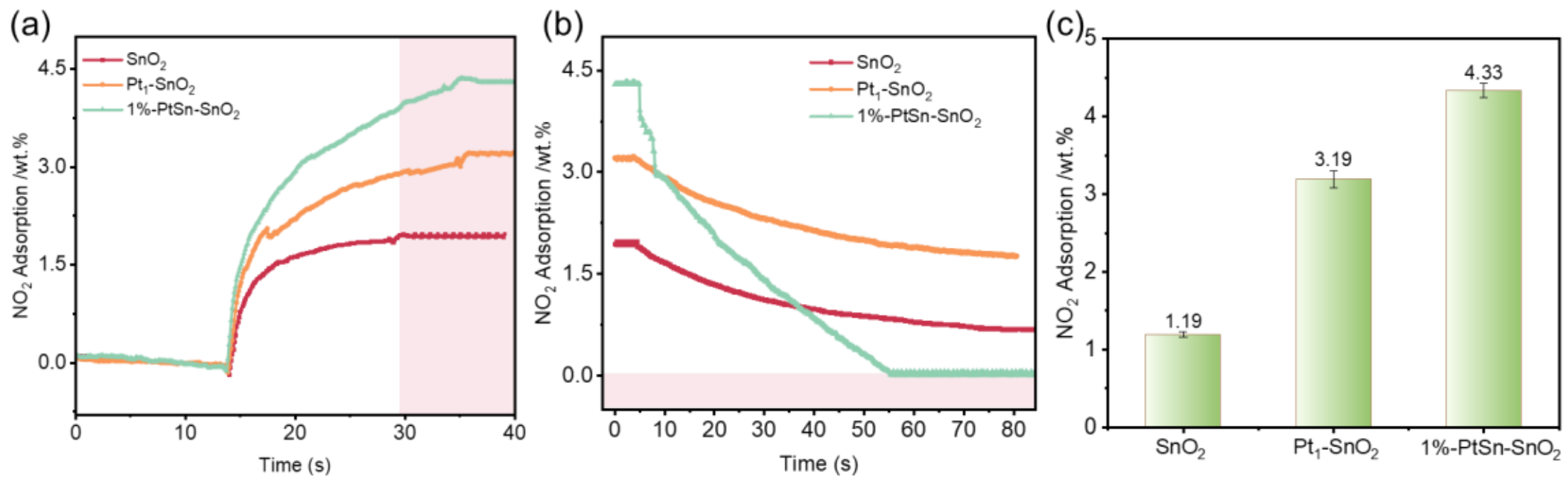

Supplementary Figure 44. (a-c) NO_2 adsorption performance tests of three samples.

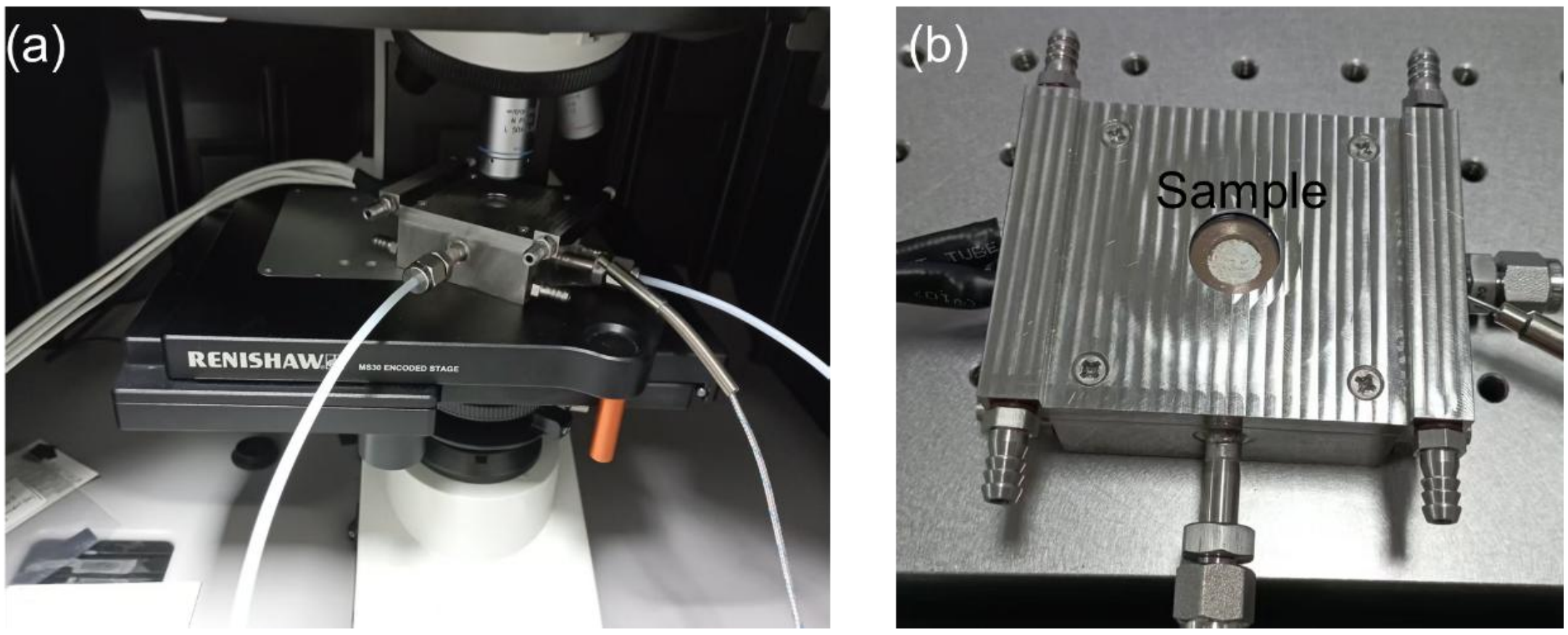

Supplementary Figure 45. (a-b) The photos of the in-situ Raman testing device.

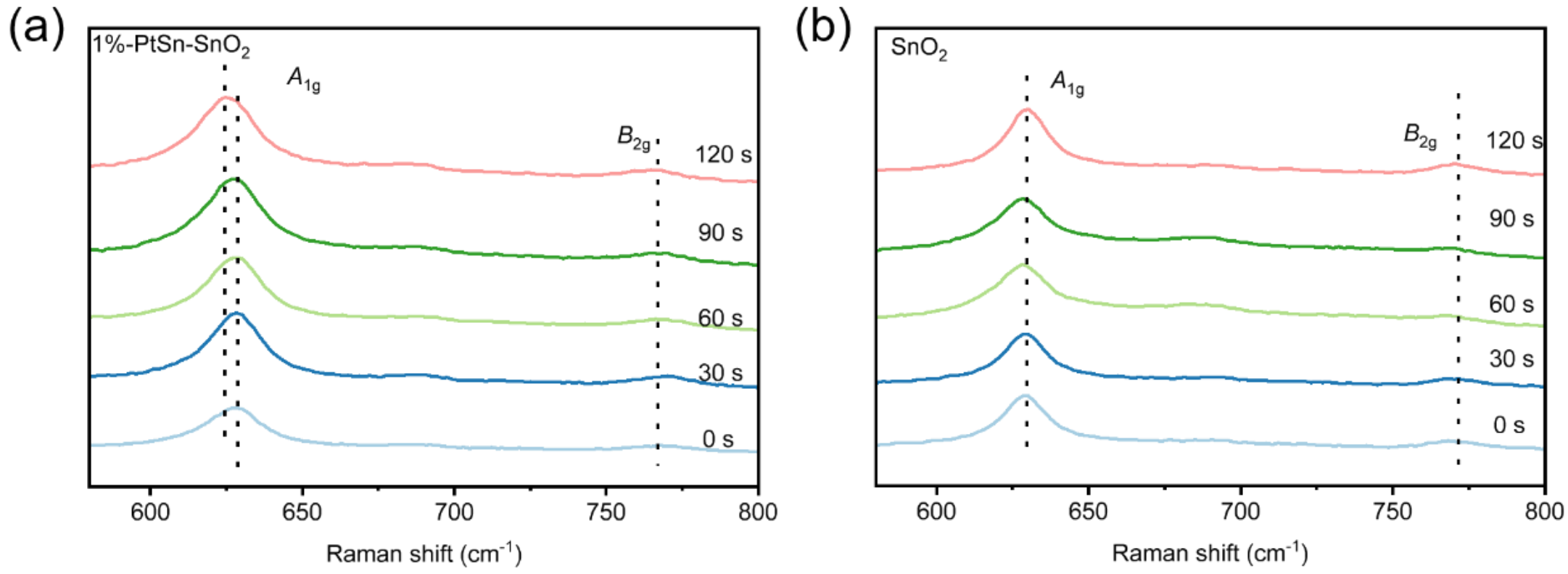

Supplementary Figure 46. In-situ Raman spectra of the (a) 1%-PtSn-SnO_2 and (b) SnO_2 when exposed to 10 ppm NO_2 over a period of 120 s.

(a)

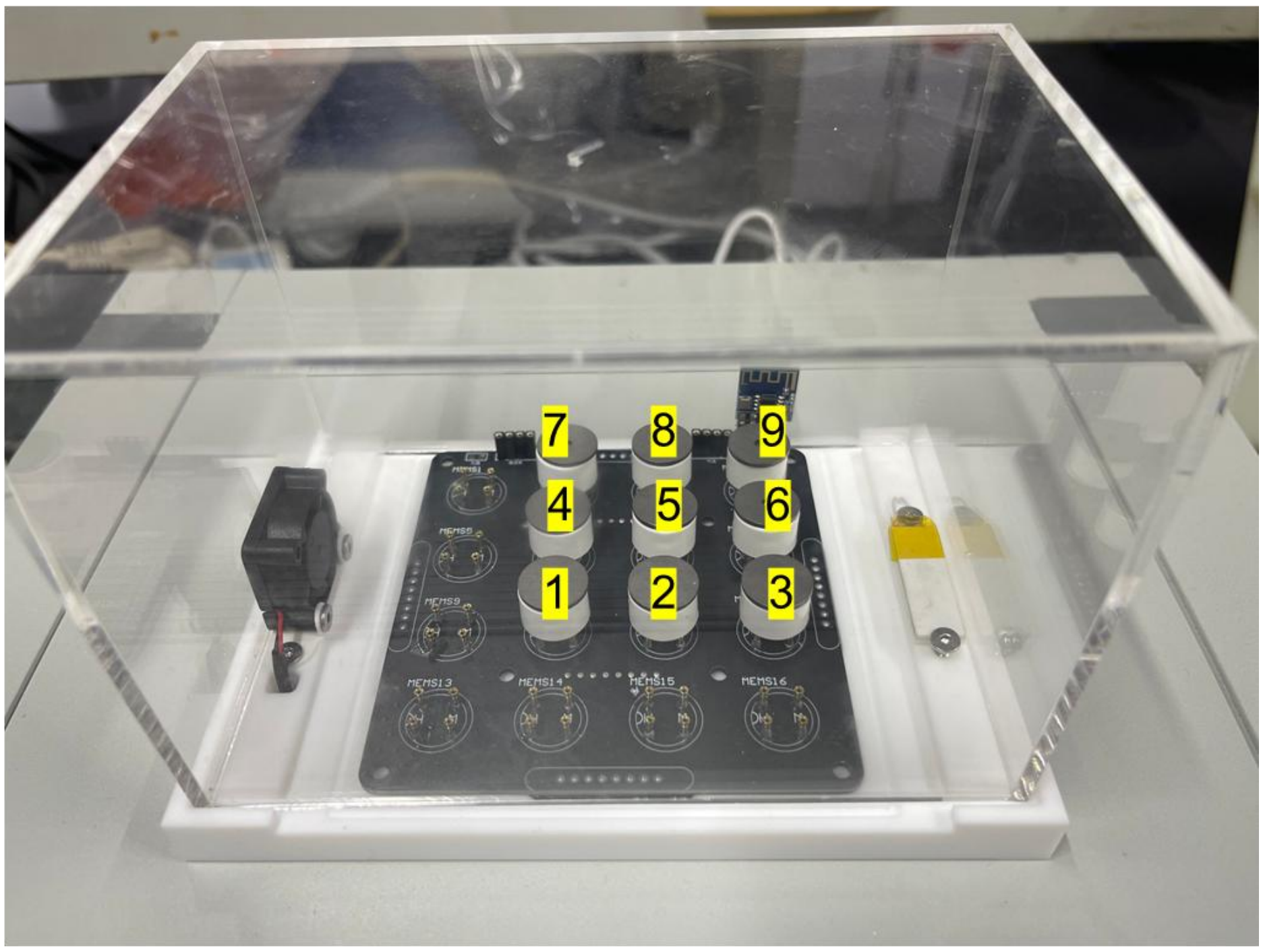

(b)

Sensor 1.SnO_2

Sensor 2.Pt_1-SnO_2

Sensor 3. Pt NPs-SnO_2

Sensor 4. 0.3%-PtSn-SnO_2

Sensor 5. 0.6%-PtSn-SnO_2

Sensor 6. 1%-PtSn-SnO_2

Sensor 7. 1%-PtSn-SnO_2

Sensor 8. 1.5%-PtSn-SnO_2

Sensor 9. 2%-PtSn-SnO_2

Supplementary Figure 47. (a) The photo of 3×3 multi-sensor array. (b) The sequence of multi-sensor array.

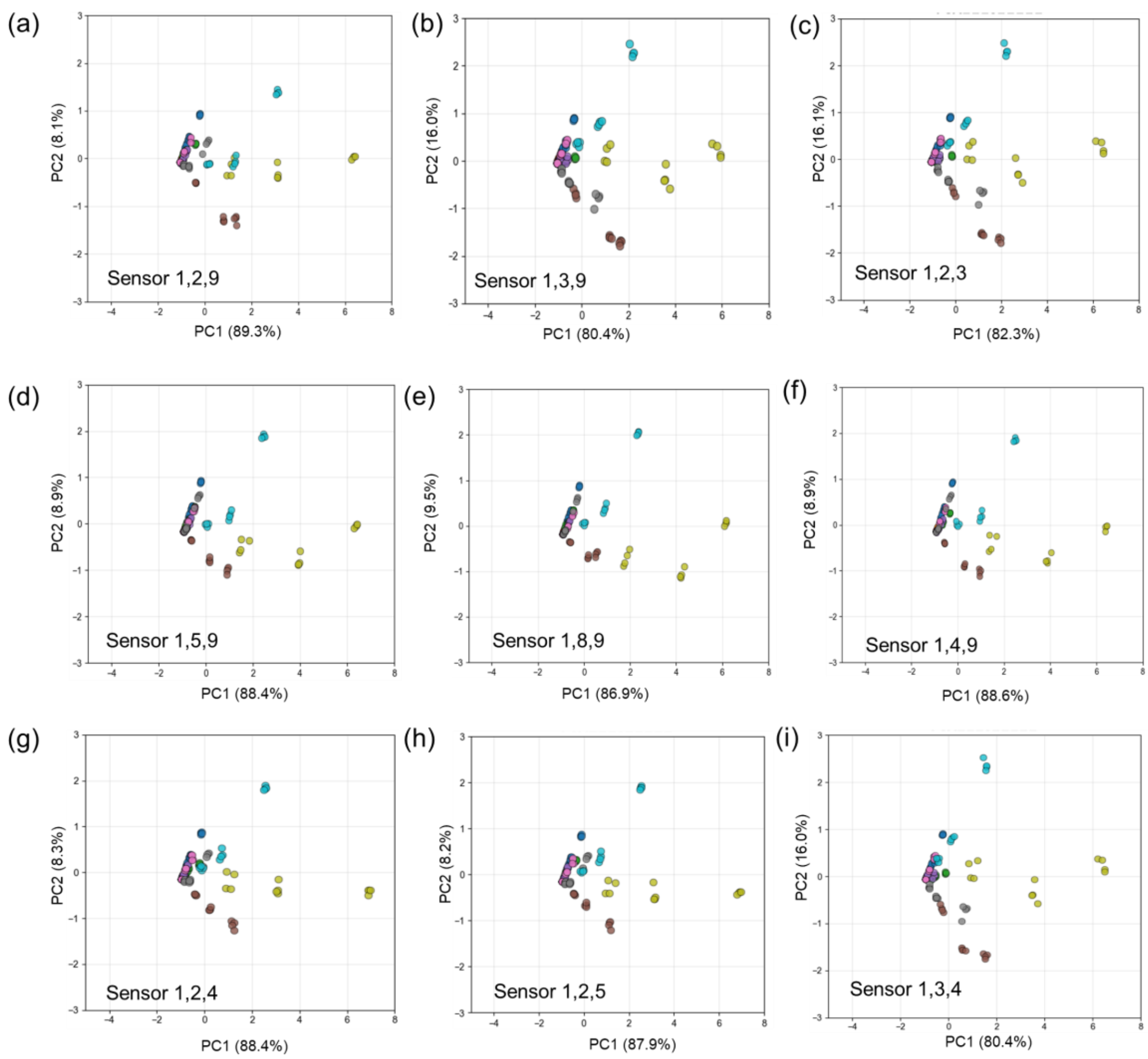

Supplementary Figure 48. PCA plot generated using data obtained from given groups of sensors (a) Sensor 1, 2, 9 (b) Sensor 1, 3, 9 (c) Sensor 1, 2, 3 (d) Sensor 1, 5, 9 (e) Sensor 1, 8, 9 (f) Sensor 1, 4, 9 (g) Sensor 1, 2, 4 (h) Sensor 1, 2, 5 (i) Sensor 1, 3, 4.

Given groups of sensors such as (a), (b) and (c) exhibit poor ability to distinguish NO_2, H_2S and SO_2. But the groups such as (d-i) show favorable discrimination for NO_2, H_2S and SO_2. Because the Sensor 4-7 have distinct characteristics and can made a significant contribution to the differentiation of specific gases.

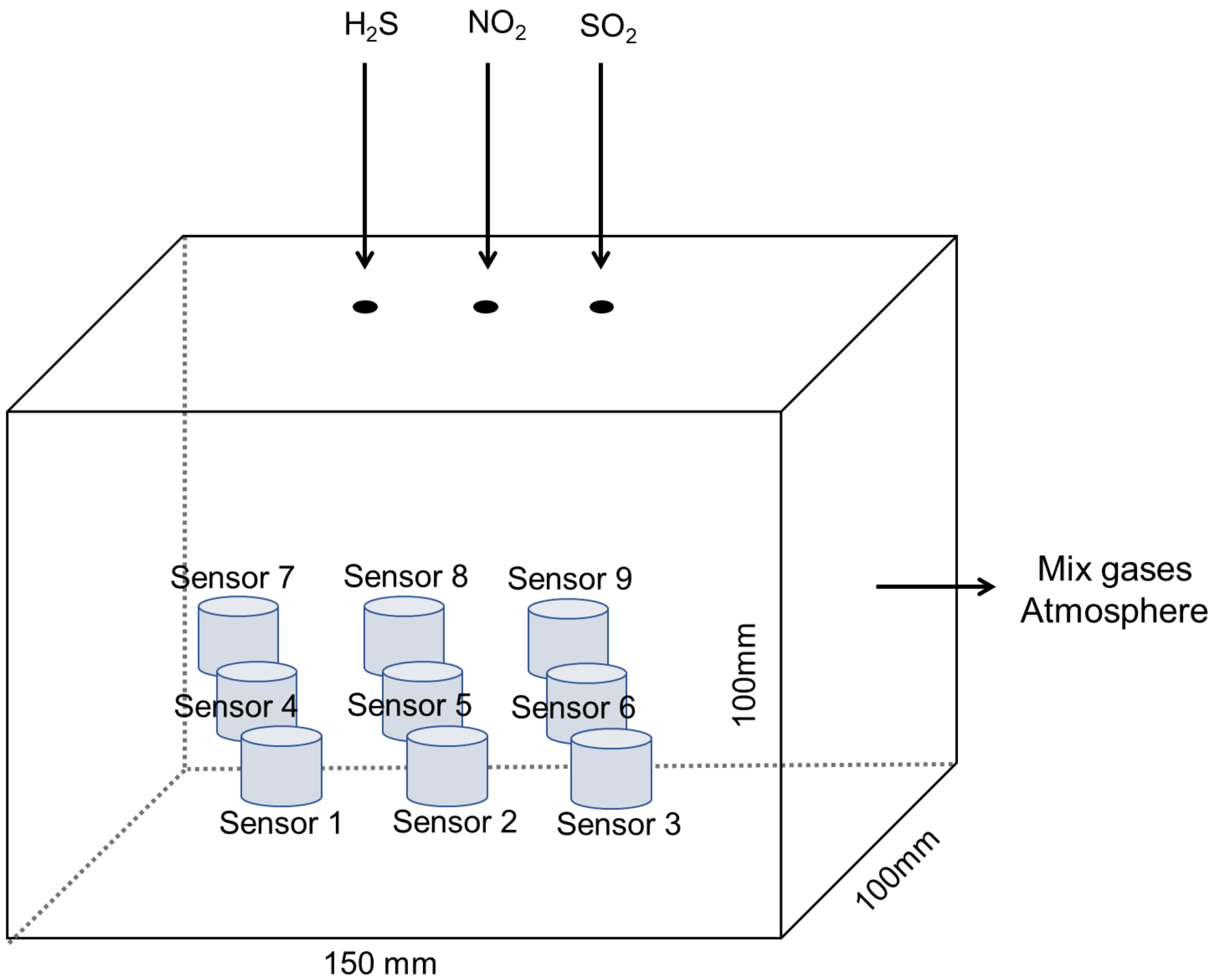

Supplementary Figure 49. Set-up diagram of gas-sensing tests in multicomponent atmospheres.

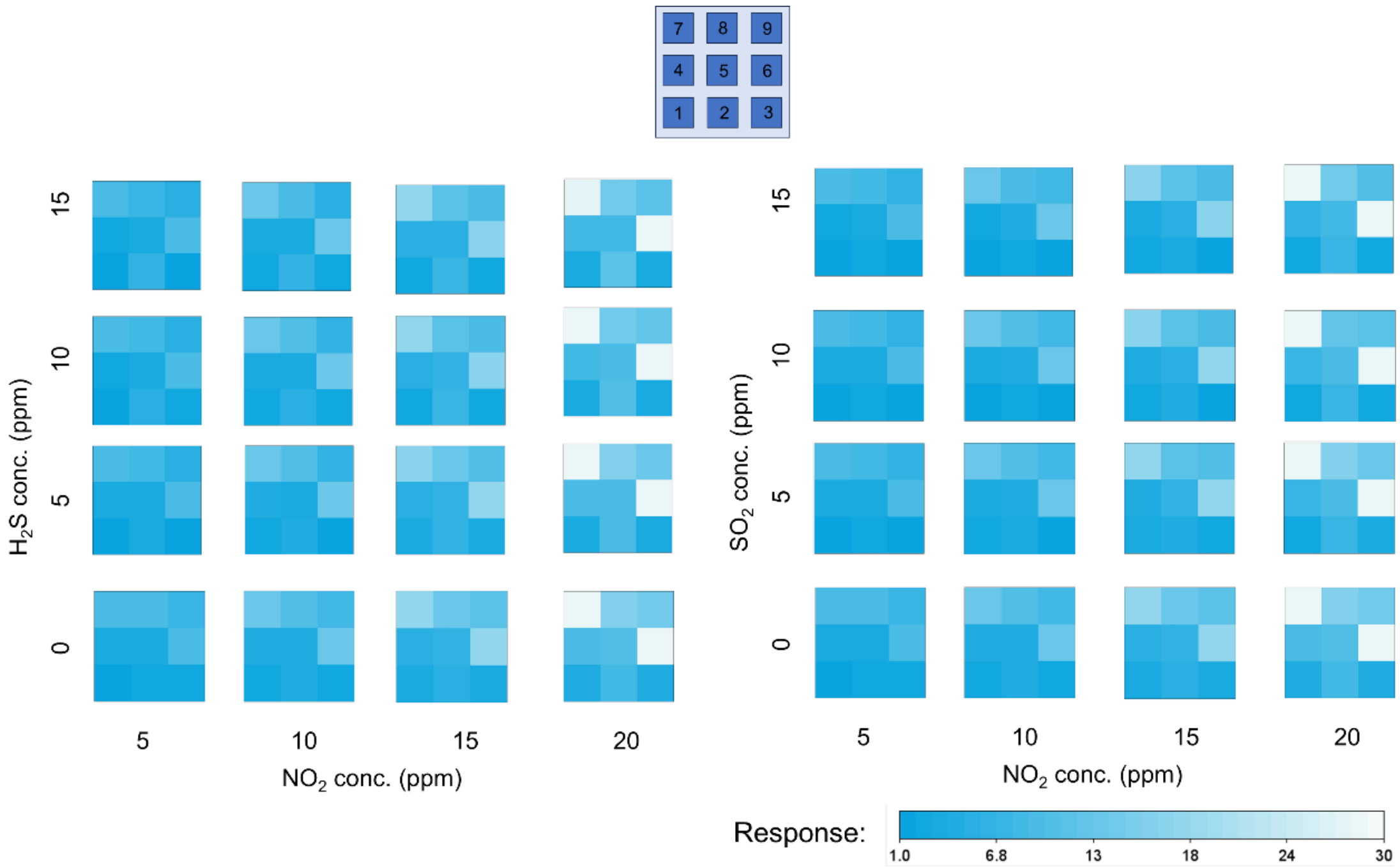

Supplementary Figure 50. Heat maps of the responses of the 3 × 3 sensor array chip to NO_2 mixing with (a) SO_2 and (b) H_2S.

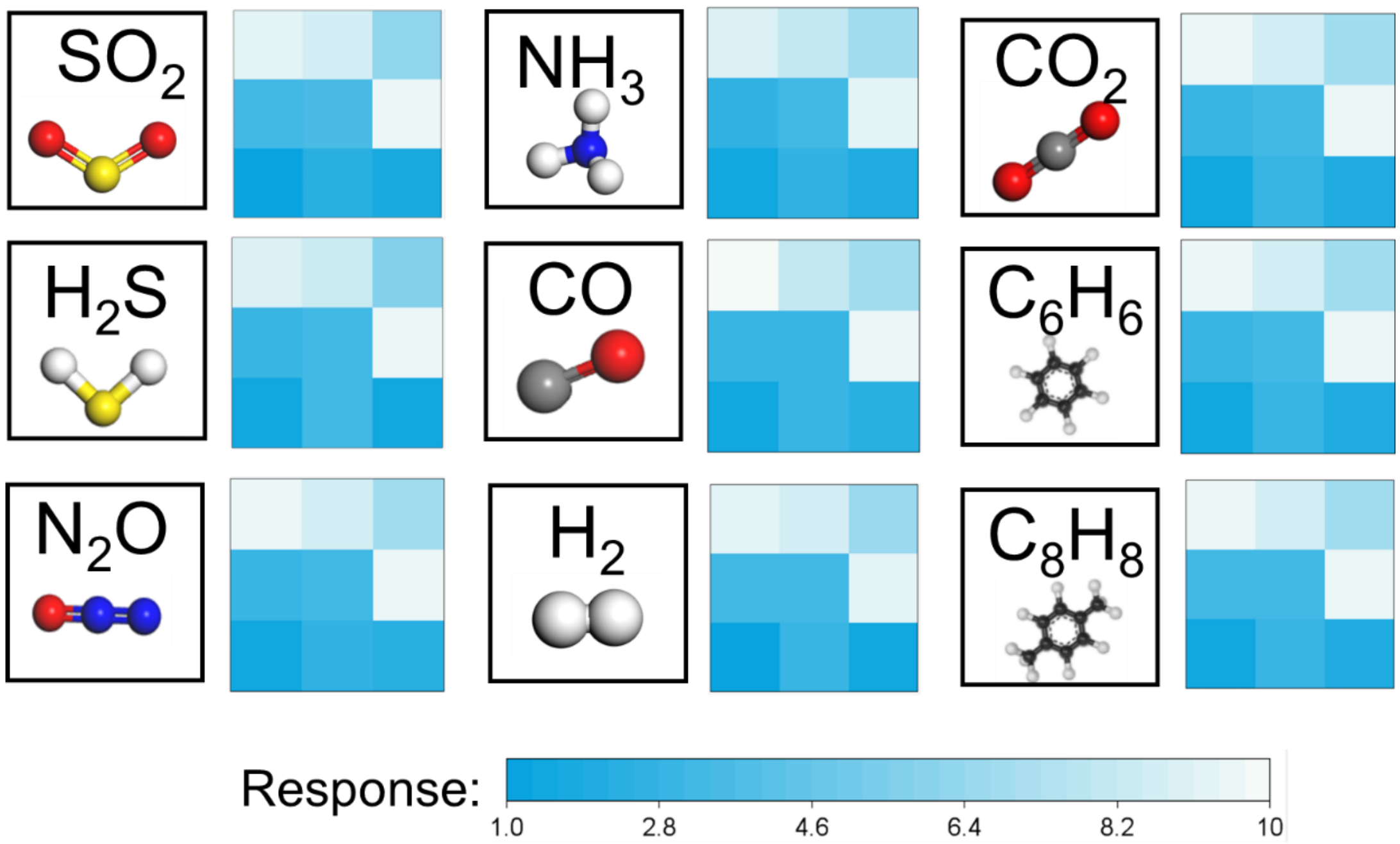

Supplementary Figure 51. Heat map of the response of the 3 × 3 sensor array chip to 5 ppm NO_2 mixing with 5 ppm other gases.

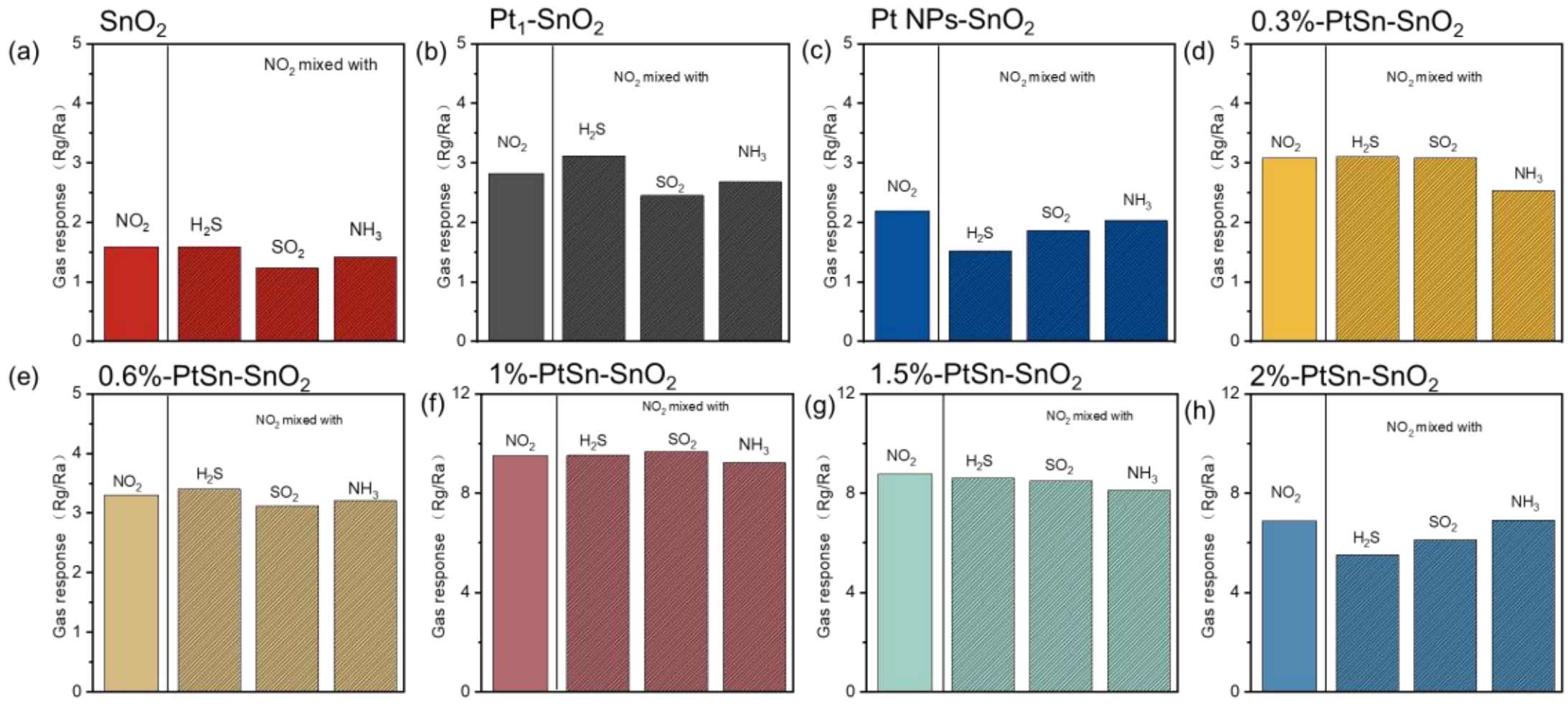

Supplementary Figure 52. Gas responses of sensors based (a) SnO_2 (b) Pt_1-SnO_2 (c) Pt NPs-SnO_2 (d) 0.3%-PtSn-SnO_2 (e) 0.6%-PtSn-SnO_2 (f) 1%-PtSn-SnO_2 (g) 1.5%-PtSn-SnO_2 (h) 2%-PtSn-SnO_2 of 5 ppm NO_2 gas mixing with 5ppm of H_2S, SO_2 and NH_3.

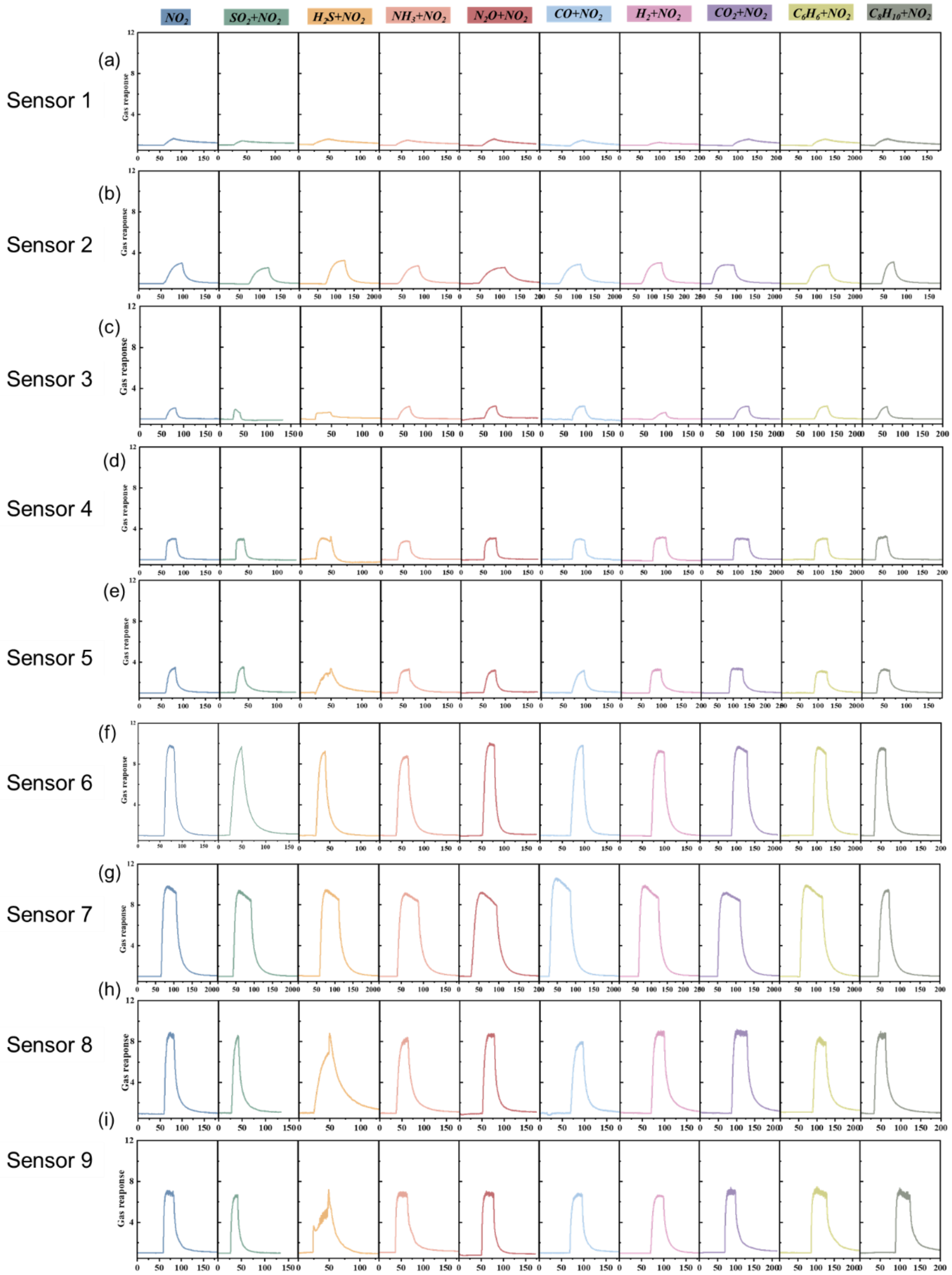

Supplementary Figure 53. Dynamic gas-sensing transients of 9 sensors under 5 ppm NO_2 and 5 ppm other gases.

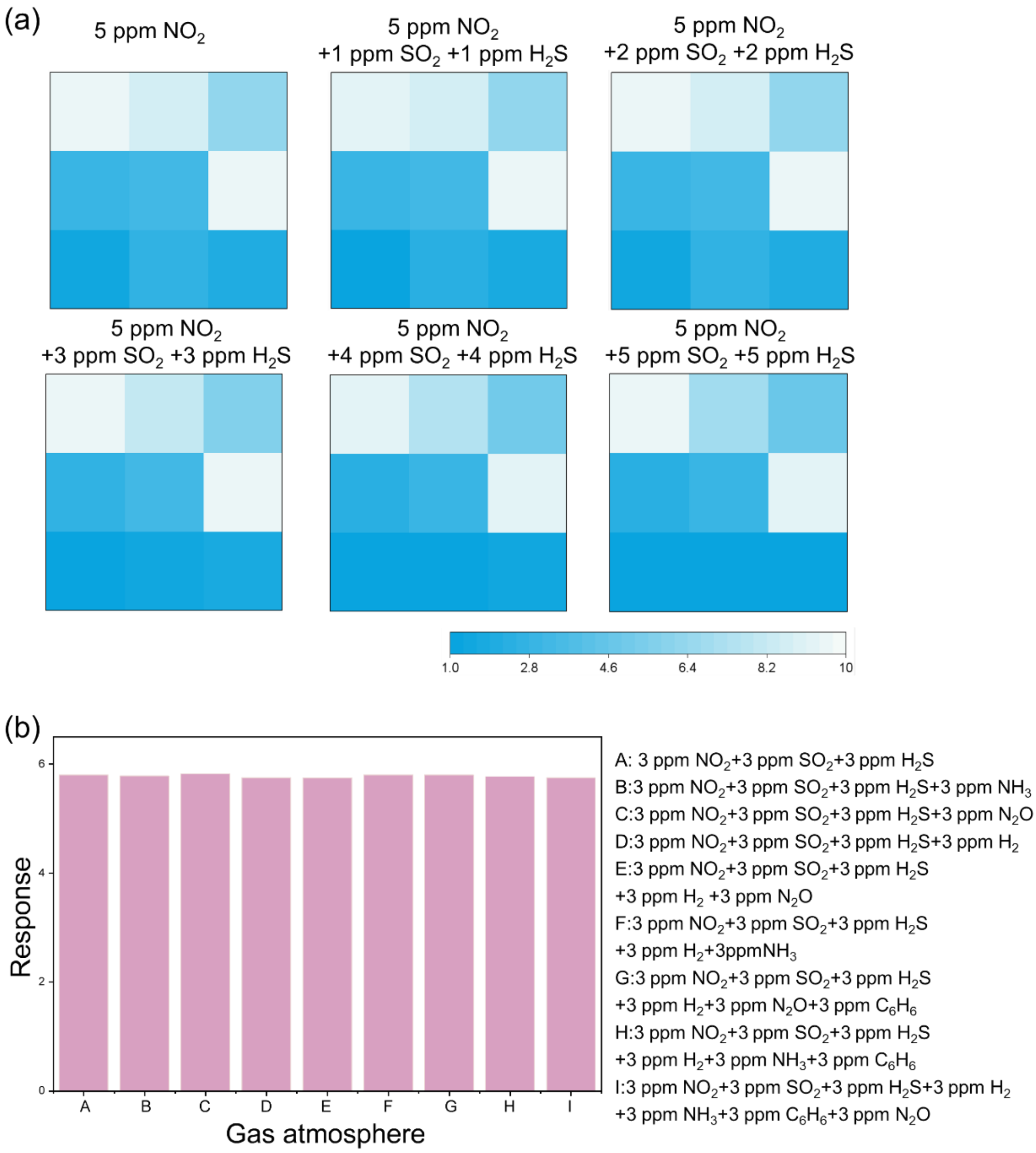

Supplementary Figure 54. Gas responses of the (a) 3 × 3 sensor array chip for 5 ppm NO_2 mixing with different concentration SO_2 and H_2S and the responses of (b) the Sensor 7 at multi-atmosphere.

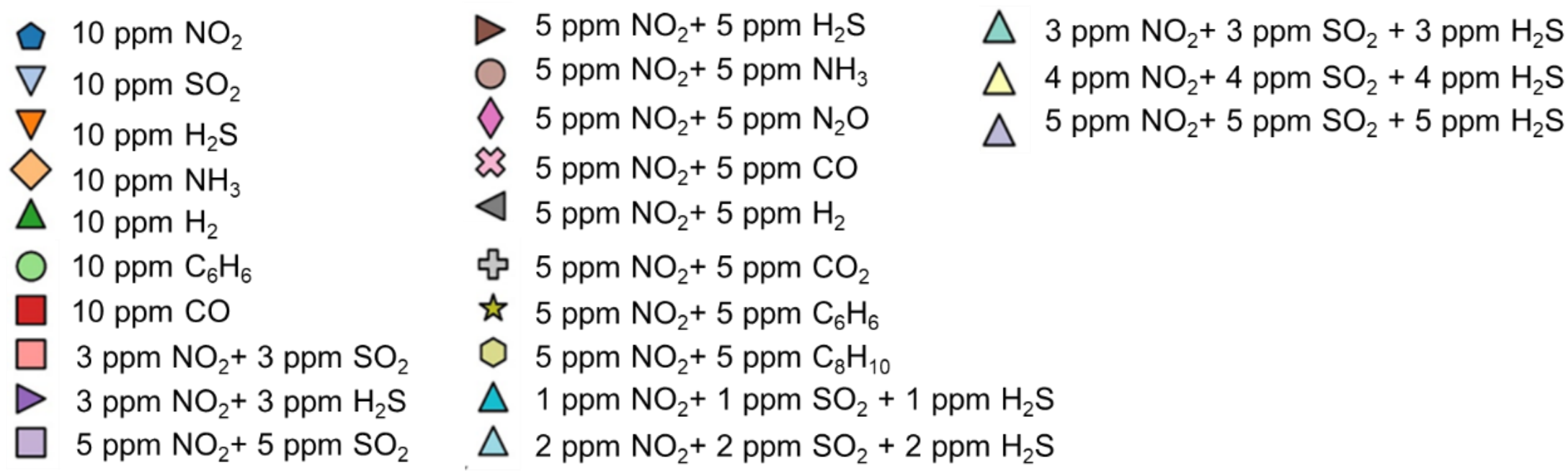

Supplementary Figure 55. The legend of Fig. 5g in the manuscription.

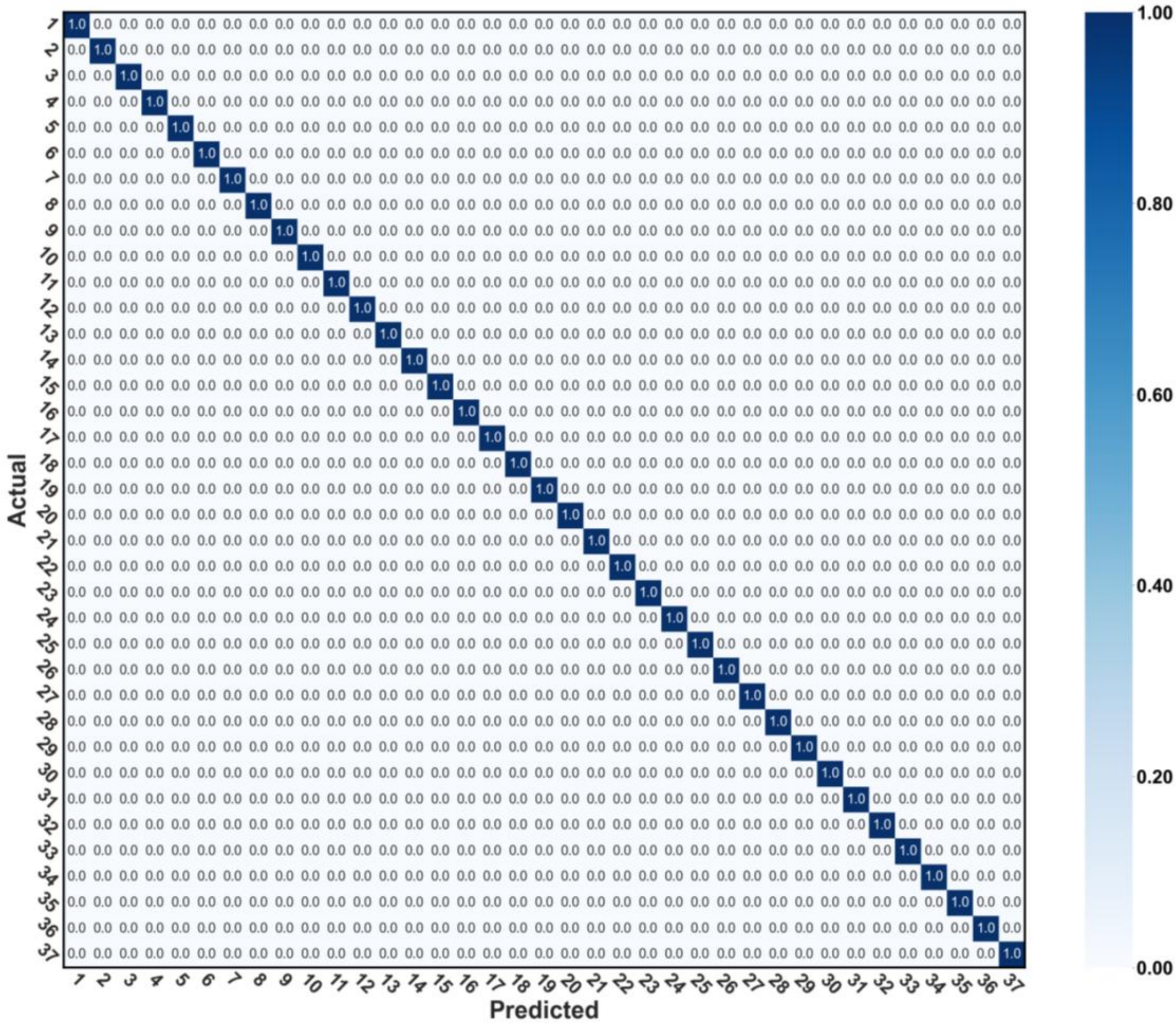

Supplementary Figure 56. Confusion matrix on classifying 37 types of gases using all sensors.

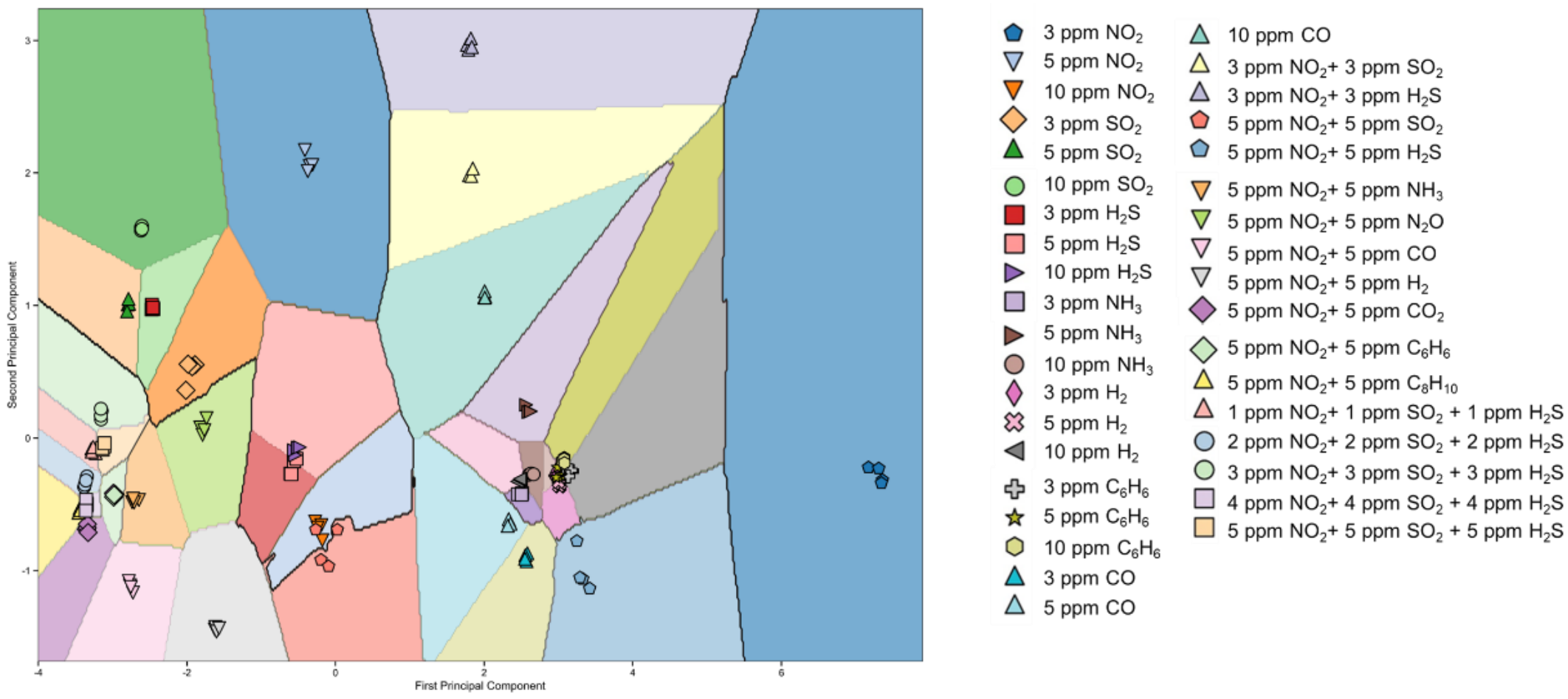

Supplementary Figure 57. KNN classification boundaries for 37 types of gases.

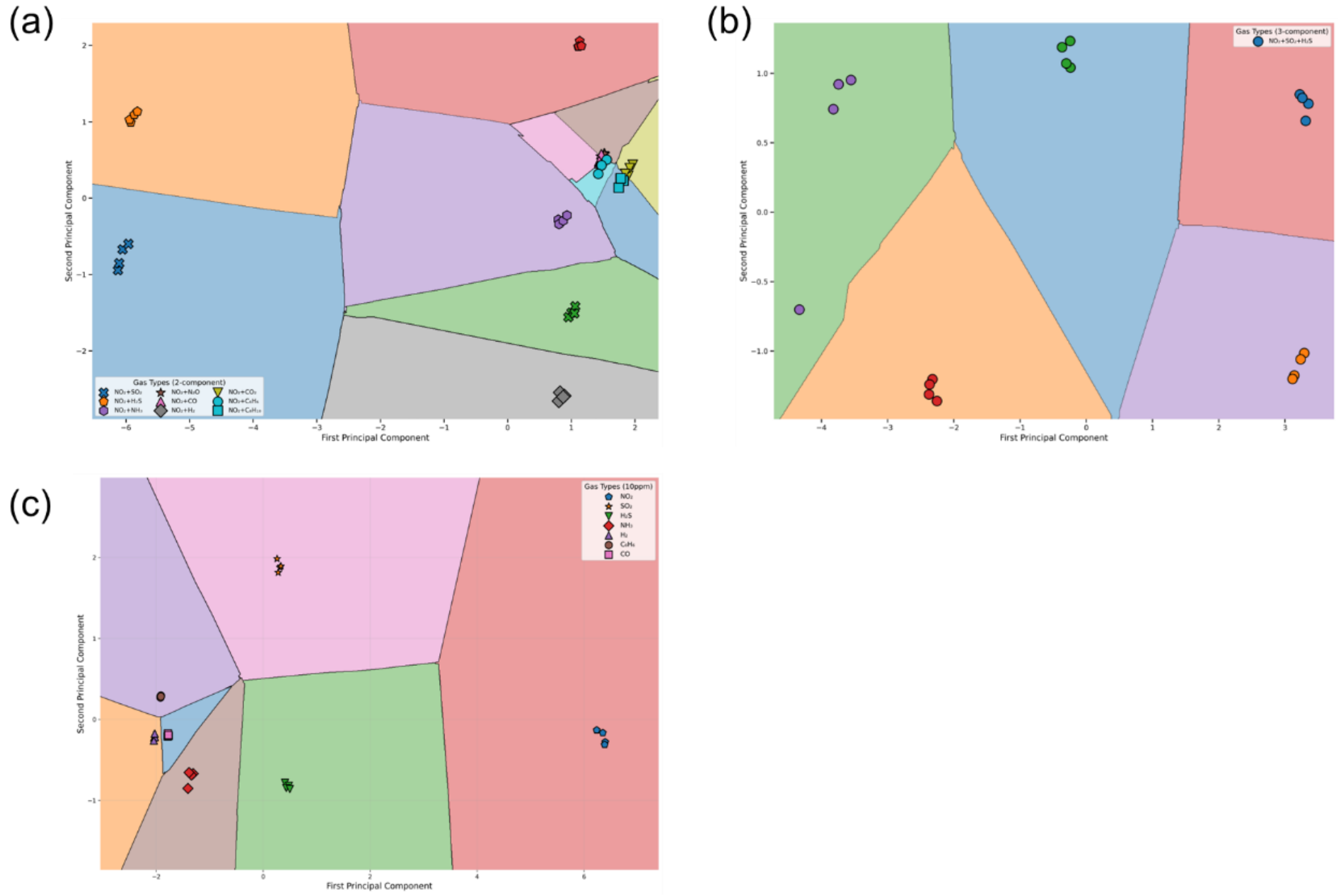

Supplementary Figure 58. KNN classification boundaries for (a) Single-component gases, (b) two-component gases and (c) multicomponent gases.

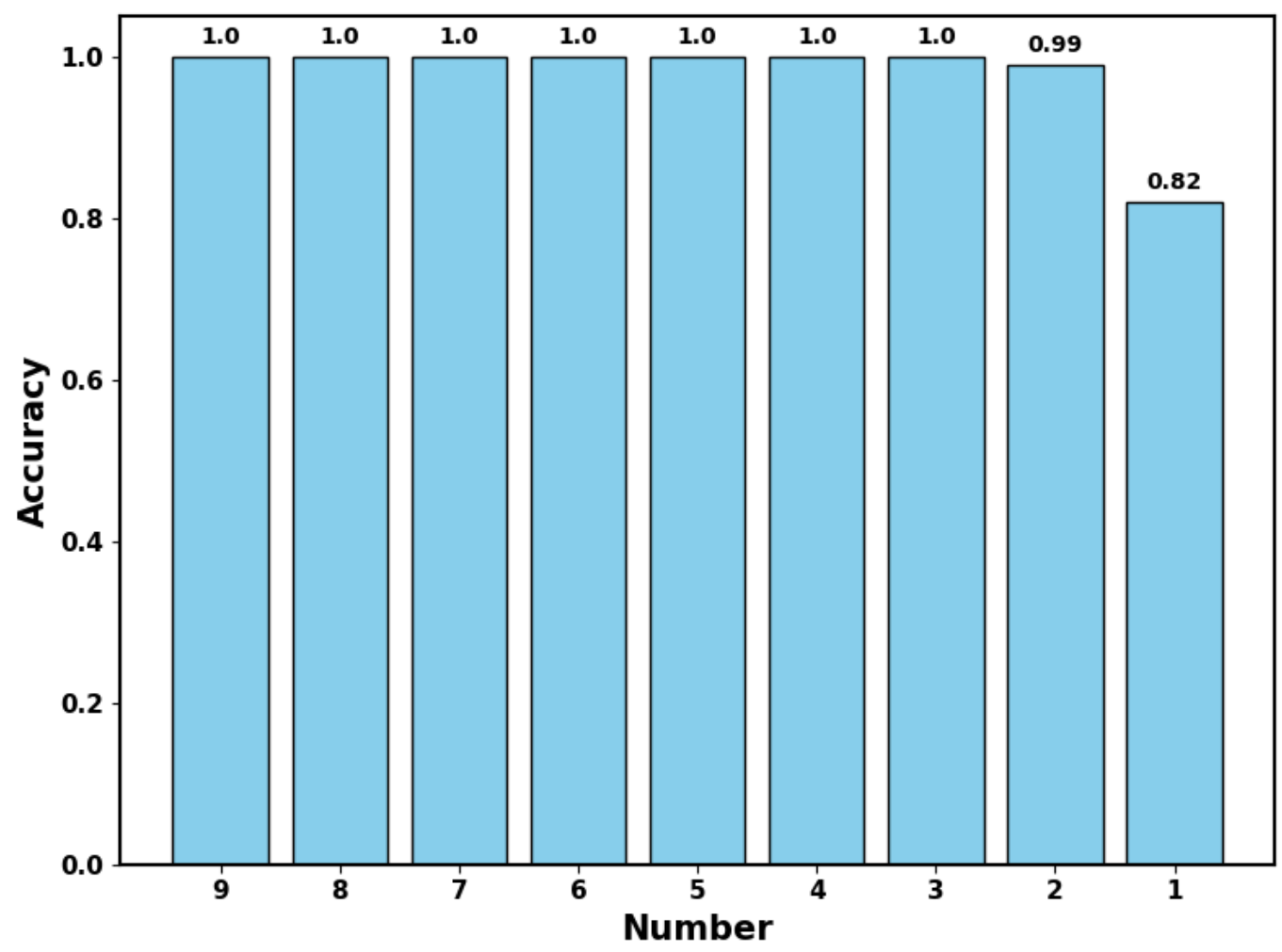

Supplementary Figure 59. The gas recognition accuracy of 9 sensor arrays with different numbers of sensors.

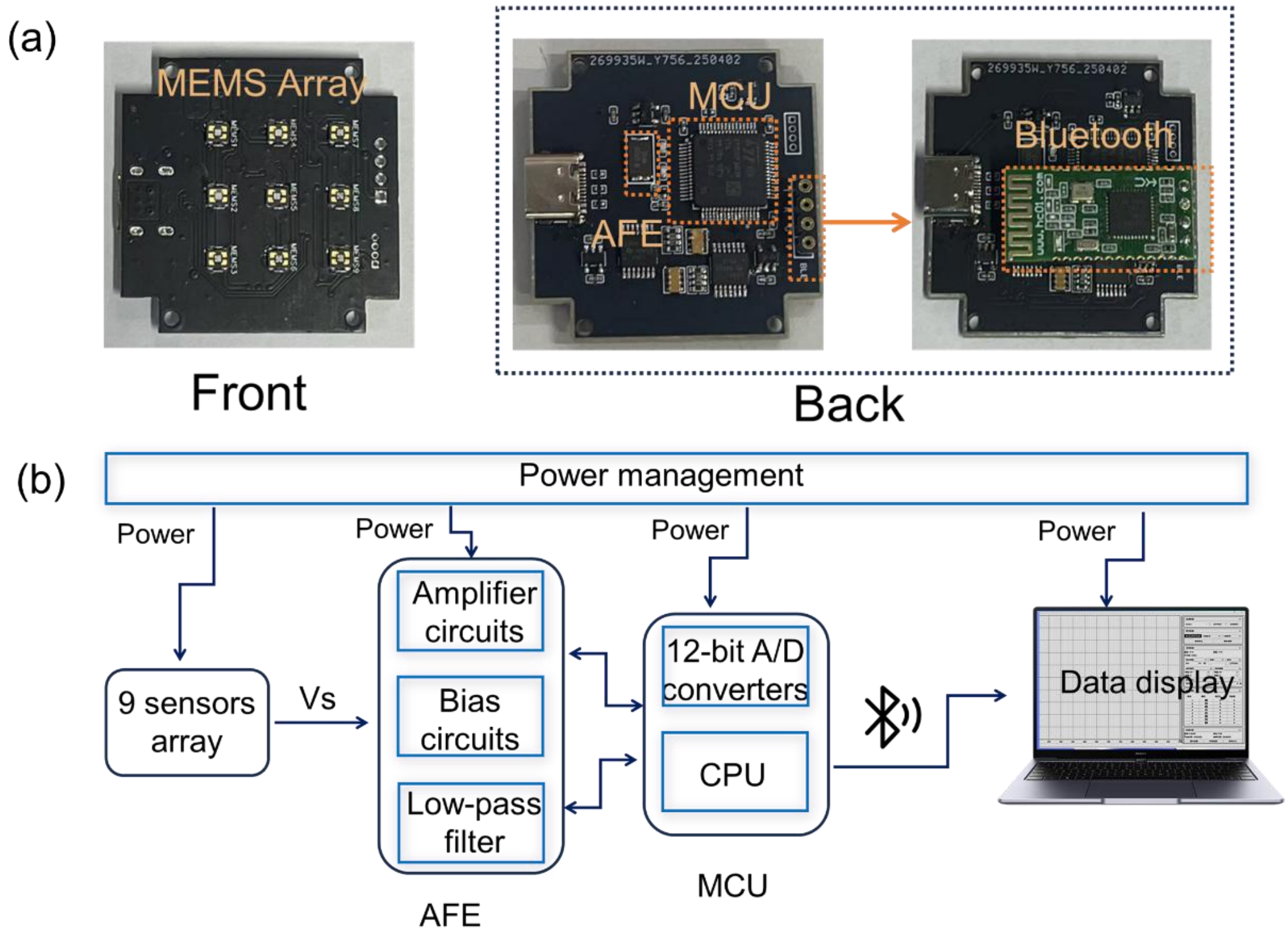

Supplementary Figure 60. (a) Photo of the 9-sensor array and corresponding block modules. (b) Detailed block diagram of the whole system.

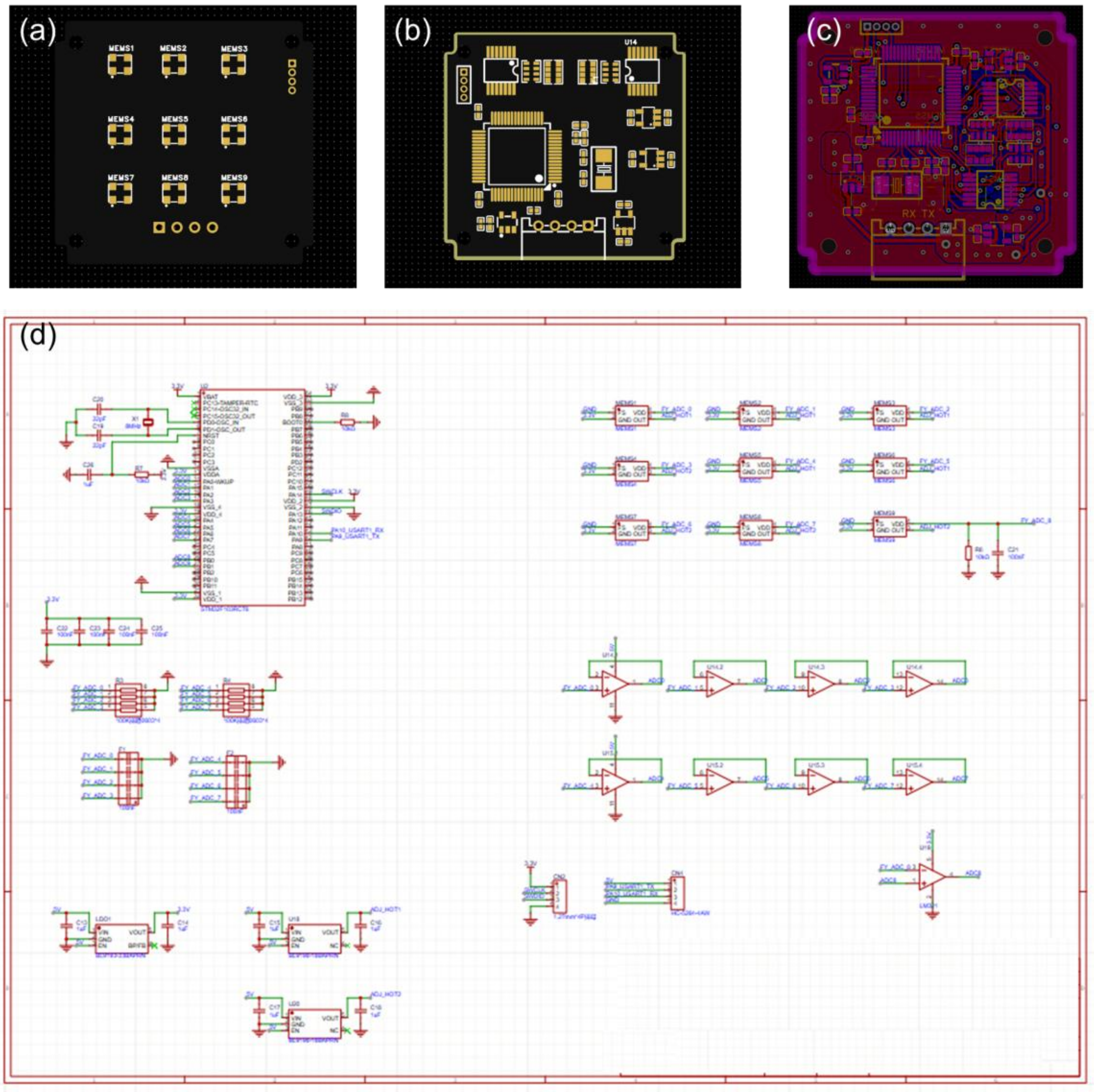

Supplementary Figure 61. (a-c) The layouts of the printed circuit board (PCB). (d) The schematic circuit diagram of circuit board.

(a)

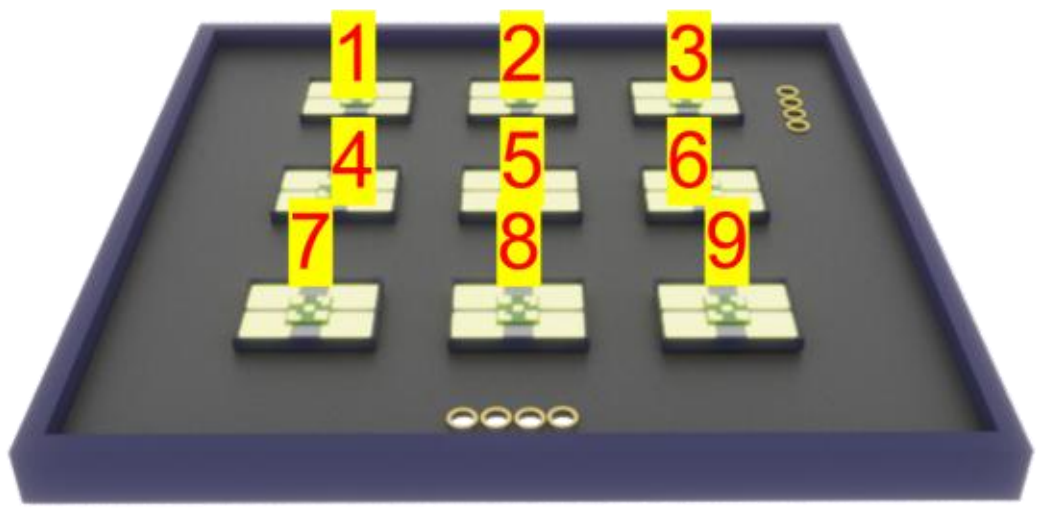

(b)

Sensor 1.SnO_2

Sensor 2.Pt_1-SnO_2

Sensor 3. Pt NPs-SnO_2

Sensor 4. 0.3%-PtSn-SnO_2

Sensor 5. 0.6%-PtSn-SnO_2

Sensor 6. 1%-PtSn-SnO_2

Sensor 7. 1%-PtSn-SnO_2

Sensor 8. 1.5%-PtSn-SnO_2

Sensor 9. 2%-PtSn-SnO_2

Supplementary Figure 62. (a) Schematic of 9-sensors array (b) The sequences of nine sensors on the 9-sensor array.

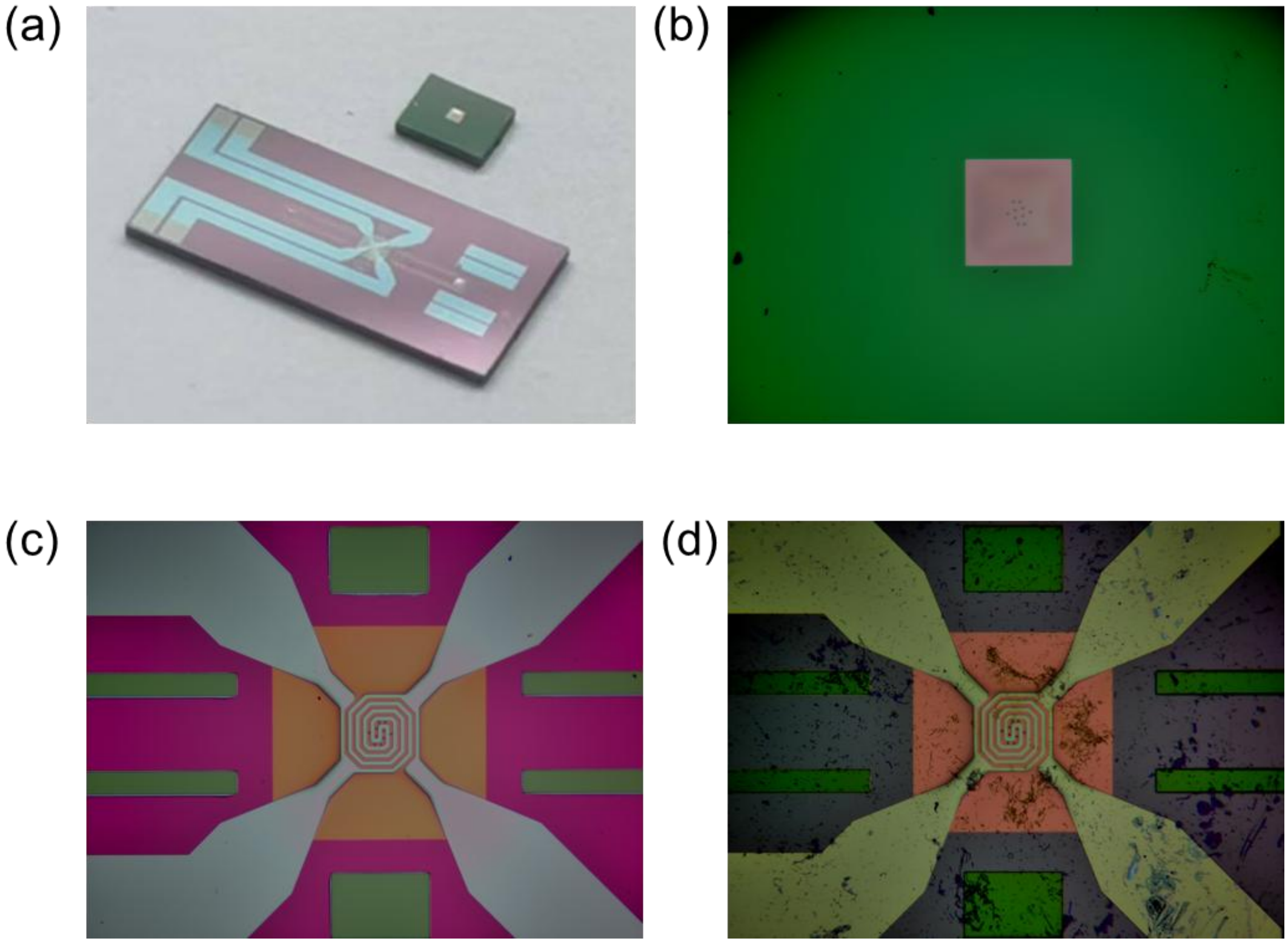

Supplementary Figure 63. (a) The photo of the upper and lower chips of in-situ TEM, Optical microscope photograph of (b)upper chip and (c) lower chip. (d)Optical microscope photograph of lower chip and samples.

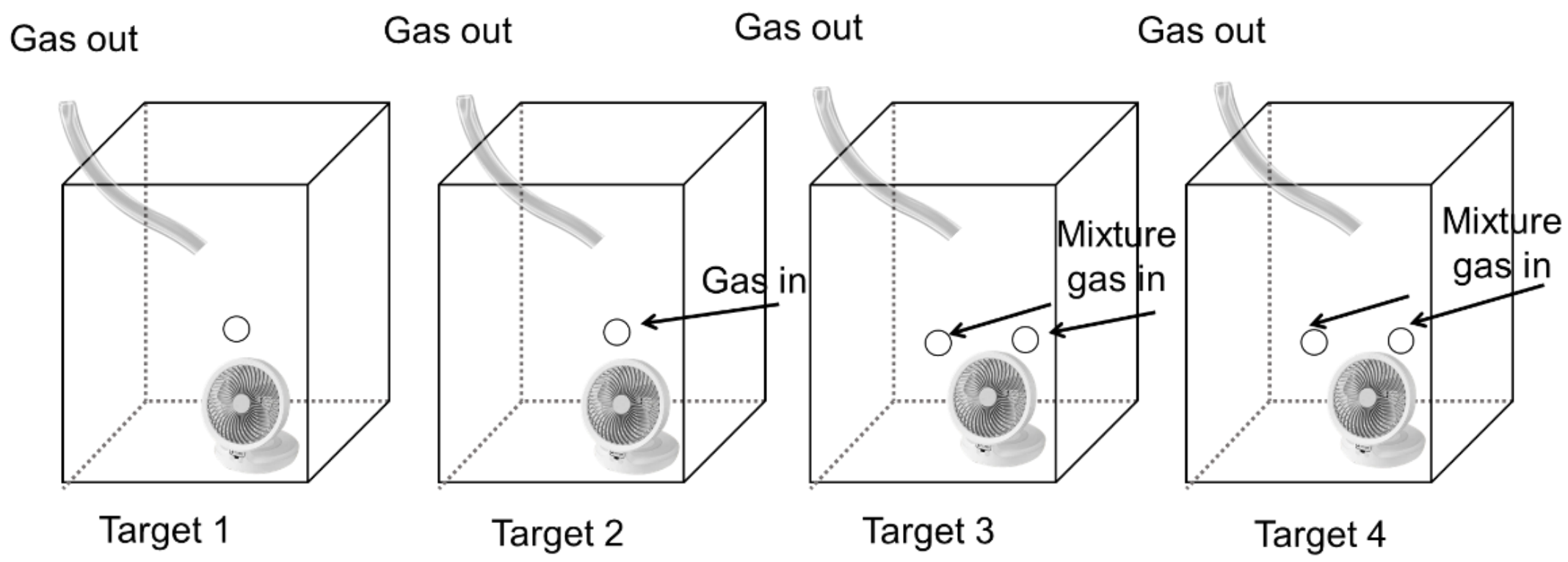

Supplementary Figure 64. Schematic diagram of cruise-monitoring experiments.

Supplementary Table 1. ICP-OES of samples

**Samples**	**Pt (μg/L)**	**Content of Pt (wt%)**
SnO_2	/	/
Pt_1-SnO_2	2.01	0.76
Pt NPs-SnO_2	2.12	0.81
0.3%-PtSn-SnO_2	2.90	0.10
0.6%-PtSn-SnO_2	1.60	0.52
1%-PtSn-SnO_2	1.97	0.72
1.5%-PtSn-SnO_2	2.55	1.1
2%-PtSn-SnO_2	2.23	1.5

Supplementary Table 2. The response time and recovery time towards 10 ppm NO_2 of four MEMS sensors.

**Sample**	**T_{res} (t)**	**T_{des}(t)**
SnO_2	20.7	40.8
Pt NPs-SnO_2	17.2	23.2
Pt_1-SnO_2	16	14.3
1%-PtSn-SnO_2	18.6	39.4

Supplementary Table 3. The K_{ads}, K_{des} and K of four samples.

**Sample**	**K_{ads} (t)**	**K_{des} (t)**	**K**
SnO_2	0.0280	0.0211	1.33
Pt_1-SnO_2	0.0434	0.1192	0.365
Pt NPs-SnO_2	0.0795	0.338	0.235
1%-PtSn-SnO_2	0.145	0.148	0.986

The adsorption (k_{ads}) and desorption (k_{des}) rate constants of all sensors were further calculated by fitting the response versus time curves:

$$R(t) \text{ for } NO_2 \text{ adsorption} = R_{max} \cdot \frac{C_a K}{1+KC_a}\left(1 - e^{\left[-\frac{1-KC_g}{K} \cdot k_{ads} t\right]}\right)$$

$$R(t) \text{ for } NO_2 \text{ desorption} = R_0 e^{[-k_{des} t]}$$

Where R_{max} is the maximum response value, R_0 is the response in air, C_a is the concentration of NO_2 gas, t is the time and K is the equilibrium constant (k_{ads}/k_{des})

Supplementary Table 4. The response time at different temperature of four sensors.

Operating Temperature/°C·K^{-1}	Response time/s			
	SnO_2	Pt NPs-SnO_2	Pt_1-SnO_2	1%-PtSn-SnO_2
70/343	23.5	20.1	18.2	19.1
90/363	22.8	18.9	17.8	18.6
110/383	22.2	18.2	17.1	17.5
130/403	21.5	17.2	16	16.2
150/423	20.7	16.3	15.8	15.2
170/443	19.2	15.2	14.5	14.2

Supplementary Table 5. The QCM tests of three sensors.

Sample	Fundamental frequency （10^6Hz）	Fundamental after coating （10^6Hz）	Frequency Shift （Hz）	Loading mass （ng）
SnO_2	10002530.45	9998933.43	3597.02	1998.3
Pt_1-SnO_2	10002535.68	9997724.27	4811.41	2673.0
1%-PtSn-SnO_2	10002530.46	9997860.15	4670.31	2594.6

The NO_2 adsorption capacity and average adsorption rate are calculated using the following formulas:

NO_2 adsorption = $\frac{Adsorbed\ NO_2\ mass}{QCM\ load\ material\ mass} \times 100\%$ (unit: wt.%)

NO_2 average adsorption rate = $\frac{NO_2\ adsorption}{response\ time\ to\ NO_2}$ (unit: wt.%/s)

Supplementary Table 6. Properties of various materials used for sensing NO_2, as reported in literature and obtained in this study.

**Active materials**	**Working Tempertaure**	**Responsivity (Rg/Ra)**	**Operating range (ppm) & LoD (ppm)**	**Response time (s)**	**Recover time (s)**	**Year**	**Ref.**
**1%-PtSn-SnO_2**	90	9.88 (5 ppm)	0.05-20 & 0.05	18.3(5 ppm)	37.5 (5 ppm)	This work	
**CuO/CeO_2**	100	4.89 (10 ppm)	0.1-50 &600 ppt	15 (10 ppm)	110(10 ppm)	2023	[16]
**SnO_2/NiO**		36 (10 ppm)	1-10 &1	163 (10 ppm)	261(10 ppm)	2018	[17]
**MoS_2-ZnO NWs**		31.2% (50 ppm)	0.2-50 &0.2	300(50 ppm)	240(50 ppm)	2018	[18]
**Cu_2O-CuO**	187	10.2 (1 ppm)	0.005-1 &0.005	35 (1 ppm)	47 (1 ppm)	2022	[19]
**SnO_2-Cu_2O C-S NWs**		200% (10 ppm)	1-10 &1	62 (5 ppm)	177(5 ppm)	2019	[20]
**ZnO/TeO_2**	100	80% (1 ppm)	0.2-1 &0.2	13 (1 ppm)	38(1 ppm)	2024	[21]
**NiO-SnO_2**		80.4% (0.05 ppm)	0.025-0.15 &0.025	75.2 (0.05 ppm)	49.1 (0.05 ppm)	2022	[22]
**Ti_3C_2Tx/Bi_2S_3**	25	12.6 (10 ppm)	0.05-1 &0.05	5 (10 ppm)	28(10 ppm)	2024	[23]
**Co_3O_4/Al_2O_3 @$Ti_3C_2T_x$**		40.3 (100 ppm)	0.01-100 &0.01	1.3 (100 ppm)	13.3(100 ppm)	2022	[24]

Active materials	Working Tempertaure	Responsivity (Rg/Ra)	Operating range (ppm) & LoD (ppm)	Response time (s)	Recover time (s)	Year	Ref.
**ZnO/MXene**		3.67 (20 ppm)	0.5-100 &0.5	22(20 ppm)	10 (20 ppm)	2022	25
**MXene/WO_3**		6.1(50 ppm)	0.5-50 %0.5	96(50 ppm)	129(50 ppm)	2021	26
**ZnO-PANI (25 wt %) composite NFs**		40 (10ppm)	0.2-50 &0.2	130(10 ppm)	140(10 ppm)	2023	27
**V_2O_5**		13%(100 ppm)	10-100 &10	4 (100 ppm)	44(100 ppm)	2022	28
**Soft template assisted m-SnO_2**	80	8.6×10^3 (10 ppm)	10	1 (10 ppm)	155 (10 ppm)	2025	29
**CuO-Co_3O_4**		48.03 (20 ppm)	0.2-20 &0.2	48.03 (20 ppm)	160 (20 ppm)	2022	30
**In_2O_3@GO**		78 (40 ppm)	10-100 &10	106s (40 ppm)	42 (40 ppm)	2022	31
**SnO_2**	25	205% (10 ppm)	0.5-10 &0.5	250 (10 ppm)	1100(10 ppm)	2023	32
**MoS_2/$Ti_3C_2T_x$**		25.1 (10 ppm)	1-50 &1	530(10 ppm)	140(10 ppm)	2022	33
**Polycrystalline ZnO Nanofibers**		16%(25 ppb)	0.02-0.2 &0.02	178 (25 ppb)	374(25 ppb)	2018	34

Supplementary Table 7. In Fig. S54, the numbers 1 to 37 in the matrix are confused with the corresponding gases.

**1**	3 ppm NO_2	**14**	5 ppm H_2	**27**	5 ppm NO_2+ 5 ppm N_2O
**2**	5 ppm NO_2	**15**	10 ppm H_2	**28**	5 ppm NO_2+ 5 ppm CO
**3**	10 ppm NO_2	**16**	3 ppm C_6H_6	**29**	5 ppm NO_2+ 5 ppm H_2
**4**	3 ppm SO_2	**17**	5 ppm C_6H_6	**30**	5 ppm NO_2+ 5 ppm CO_2
**5**	5 ppm SO_2	**18**	10 ppm C_6H_6	**31**	5 ppm NO_2+ 5 ppm C_6H_6
**6**	10 ppm SO_2	**19**	3 ppm CO	**32**	5 ppm NO_2+ 5 ppm C_8H_{10}
**7**	3 ppm H_2S	**20**	5 ppm CO	**33**	1 ppm NO_2+ 1 ppm SO_2 + 1 ppm H_2S
**8**	5 ppm H_2S	**21**	10 ppm CO	**34**	2 ppm NO_2+ 2 ppm SO_2 + 2 ppm H_2S
**9**	10 ppm H_2S	**22**	3 ppm NO_2+ 3 ppm SO_2	**35**	3 ppm NO_2+ 3 ppm SO_2 + 3 ppm H_2S
**10**	3 ppm NH_3	**23**	3 ppm NO_2+ 3 ppm H_2S	**36**	4 ppm NO_2+ 4 ppm SO_2 + 4 ppm H_2S
**11**	5 ppm NH_3	**24**	5 ppm NO_2+ 5 ppm SO_2	**37**	5 ppm NO_2+ 5 ppm SO_2 + 5 ppm H_2S
**12**	10 ppm NH_3	**25**	5 ppm NO_2+ 5 ppm H_2S		
**13**	3 ppm H_2	**26**	5 ppm NO_2+ 5 ppm NH_3		

Supplementary Table 8. In Fig. 5f, the numbers 1 to 23 in the matrix are confused with the corresponding gases.

**1**	10 ppm NO_2	**14**	5 ppm NO_2+ 5 ppm CO
**2**	10 ppm SO_2	**15**	5 ppm NO_2+ 5 ppm H_2
**3**	10 ppm H_2S	**16**	5 ppm NO_2+ 5 ppm CO_2
**4**	10 ppm NH_3	**17**	5 ppm NO_2+ 5 ppm C_6H_6
**5**	10 ppm H_2	**18**	5 ppm NO_2+ 5 ppm C_8H_{10}
**6**	10 ppm C_6H_6	**19**	1 ppm NO_2+ 1 ppm SO_2 + 1 ppm H_2S
**7**	10 ppm CO	**20**	2 ppm NO_2+ 2 ppm SO_2 + 2 ppm H_2S
**8**	3 ppm NO_2+ 3 ppm SO_2	**21**	3 ppm NO_2+ 3 ppm SO_2 + 3 ppm H_2S
**9**	3 ppm NO_2+ 3 ppm H_2S	**22**	4 ppm NO_2+ 4 ppm SO_2 + 4 ppm H_2S
**10**	5 ppm NO_2+ 5 ppm SO_2	**23**	5 ppm NO_2+ 5 ppm SO_2 + 5 ppm H_2S
**11**	5 ppm NO_2+ 5 ppm H_2S		
**12**	5 ppm NO_2+ 5 ppm NH_3		
**13**	5 ppm NO_2+ 5 ppm N_2O		

Supplementary References

1. Wang, X. *et al.* Quantitative structure–activity relationship of nanowire adsorption to SO_2 revealed by in situ TEM technique. *Nano Lett.* **21**, 1679–1687 (2021).
2. Wang, X. *et al.* In situ TEM technique revealing the deactivation mechanism of bimetallic Pd–Ag nanoparticles in hydrogen sensors. *Nano Lett.* **22**, 3157–3164 (2022).
3. Wang, X. *et al.* Uncoordinated amine groups of metal–organic frameworks to anchor single Ru sites as chemoselective catalysts toward the hydrogenation of quinoline. *J. Am. Chem. Soc.* **139**, 9419–9422 (2017).
4. Luo, N. *et al.* Ultralow detection limit MEMS hydrogen sensor based on SnO_2 with oxygen vacancies. *Sensors and Actuators B: Chemical* **354**, 130982 (2022).
5. Guo, M. *et al.* Fast-response MEMS xylene gas sensor based on CuO/WO_3 hierarchical structure. *Journal of Hazardous Materials* **429**, 127471 (2022).
6. Qiao, X. *et al.* Progresses on the theory and application of quartz crystal microbalance. *Appl. Phys. Rev.* **3**, 031106 (2016).
7. Perdew, J. P. *et al.* Generalized gradient approximation made simple. *Phys. Rev. Lett.* **77**, 3865–3868 (1996).
8. Kresse, G. *et al.* Efficient iterative schemes for *ab initio* total-energy calculations using a plane-wave basis set. *Phys. Rev. B* **54**, 11169–11186 (1996).
9. Grimme, S. *et al.* H. A consistent and accurate *ab initio* parametrization of density functional dispersion correction (DFT-D) for the 94 elements H-Pu. *The Journal of Chemical Physics* **132**, 154104 (2010).

10. Petousis, I. *et al.* High-throughput screening of inorganic compounds for the discovery of novel dielectric and optical materials. *Scientific Data* **4**, 160134 (2017).

11. Jain, A. *et al.* Commentary: The Materials Project: A materials genome approach to accelerating materials innovation. *APL Mater.* **1**, 011002 (2013).

12. Wen, M. *et al.* A KIM-compliant potfit for fitting sloppy interatomic potentials: application to the EDIP model for silicon. *Modelling Simul. Mater. Sci. Eng.* **25**, 014001 (2016).

13. Sun, Y. *et al.* Mesoporous high-entropy oxides nanoplatform decodes paired-plasma metabolic fingerprinting of pancreatic cancer. *Advanced Materials* **n/a**, e13988.

14. Plimpton, S. Fast parallel algorithms for short-range molecular dynamics. *Journal of Computational Physics* **117**, 1–19 (1995).

15. Stukowski, A. Visualization and analysis of atomistic simulation data with OVITO–the Open Visualization Tool. *Modelling Simul. Mater. Sci. Eng.* **18**, 015012 (2009).

16. Fang, H. *et al.* A chemiresistive ppt level NO_2 gas sensor based on CeO2 nanoparticles modified CuO nanosheets operated at 100 °C. *Sensors and Actuators B: Chemical* **393**, 134277 (2023).

17. Kim, J.-H. *et al.* SnO_2 (*n*)-NiO (*p*) composite nanowebs: Gas sensing properties and sensing mechanisms. *Sensors and Actuators B: Chemical* **258**, 204–214 (2018).

18. Zhao, S. *et al.* Vertically aligned MoS_2/ZnO nanowires nanostructures with highly enhanced NO_2 sensing activities. *Applied Surface Science* **456**, 808–816 (2018).

19. Wang, N. *et al.* Highly sensitive and selective NO_2 gas sensor fabricated from

Cu_2O-CuO microflowers. *Sensors and Actuators B: Chemical* **362**, 131803 (2022).

20. Kim, J.-H. *et al.* Enhancement of CO and NO_2 sensing in n-SnO_2-p-Cu_2O core-shell nanofibers by shell optimization. *Journal of Hazardous Materials* **376**, 68–82 (2019).

21. Nagarjuna, Y. *et al.* TeO_2 doped ZnO nanostructure for the enhanced NO_2 gas sensing on MEMS sensor device. *Sensors and Actuators B: Chemical* **401**, 134891 (2024).

22. Ding, Y. *et al.* An ultrasensitive NO_2 gas sensor based on a NiO-SnO_2 composite with a sub-ppb detection limit at room temperature. *Sensors and Actuators B: Chemical* **414**, 135916 (2024).

23. Li, C. *et al.* $Ti_3C_2T_x$ nanosheet/Bi_2S_3 nanobelt composites for NO_2 gas sensing. *ACS Appl. Nano Mater.* **7**, 13062–13070 (2024).

24. Sun, B. *et al.* Room-temperature gas sensors based on three-dimensional Co_3O_4/Al_2O_3@$Ti_3C_2T_x$ MXene nanocomposite for highly sensitive NO_x detection. *Sensors and Actuators B: Chemical* **368**, 132206 (2022).

25. Fan, C. *et al.* Fast and recoverable NO_2 detection achieved by assembling ZnO on $Ti_3C_2T_x$ MXene nanosheets under UV illumination at room temperature. *Nanoscale* **14**, 3441–3451 (2022).

26. Wang, D. *et al.* Multifunctional poly(vinyl alcohol)/Ag nanofibers-based triboelectric nanogenerator for self-powered MXene/tungsten oxide nanohybrid NO_2 gas sensor. *Nano Energy* **89**, 106410 (2021).

27. Bonyani, M. *et al.* Enhanced NO_2 gas sensing properties of ZnO-PANI composite

nanofibers. *Ceramics International* **49**, 1238–1249 (2023).

28. Babar, B. M. *et al.* Concentration modulated vanadium oxide nanostructures for NO_2 gas sensing. *Sensors and Actuators B: Chemical* **351**, 130947 (2022).
29. Singh, A. *et al.* Low temperature NO_2 gas sensing achieved by mesoporous SnO_2. *Journal of Alloys and Compounds* **1013**, 178592 (2025).
30. Fang, H. *et al.* Enhanced NO_2 gas sensing performance by hierarchical CuO–Co_3O_4 spheres. *Sensors and Actuators B: Chemical* **352**, 131068 (2022).
31. Shah, S. *et al.* NO_2 gas sensing responses of In_2O_3 nanoparticles decorated on GO nanosheets. *Ceramics International* **48**, 12291–12298 (2022).
32. Cheng, Y. F. *et al.* Liquid-tin-printed two-dimensional SnO for optoelectronic NO_2 gas sensing at room temperature. *J. Mater. Chem. C* **11**, 14187–14198 (2023).
33. Thanh Hoai Ta, Q. *et al.* Improved NO_2 gas sensing performance of 2D $MoS_2/Ti_3C_2T_x$ MXene nanocomposite. *Applied Surface Science* **604**, 154624 (2022).
34. Aziz, A. *et al.* Core–Shell Electrospun Polycrystalline ZnO Nanofibers for Ultra-Sensitive NO_2 Gas Sensing. *ACS Appl. Mater. Interfaces* **10**, 43817–43823 (2018).
