# Supplementary material for: Creating locally enriched single-atom catalysts via bio-inspired internalization process for interference-resistant NO2 detection in complex environments
Source: Nat Commun. 2026 Aug 20;17:9949. doi: 10.1038/s41467-026-76850-4 (PMC13586179; doi:10.1038/s41467-026-76850-4)
Supplement: Supplementary file 2 [file 41467_2026_76850_MOESM2_ESM.pdf]

**Description of Additional Supplementary Files**

**Supplementary Movie 1:** In situ TEM recording of the structural evolution during pyrolysis of 1.0%-PtSn-Sn nanofibers in air.
