# Supplementary material for: Creating locally enriched single-atom catalysts via bio-inspired internalization process for interference-resistant NO2 detection in complex environments
Source: Nat Commun. 2026 Aug 20;17:9949. doi: 10.1038/s41467-026-76850-4 (PMC13586179; doi:10.1038/s41467-026-76850-4)
Supplement: Supplementary file 3 [file 41467_2026_76850_MOESM4_ESM.pdf]

nature portfolio

Corresponding author(s): Zhenggang Xue

Last updated by author(s): Jul 24, 2026

Reporting Summary

Nature Portfolio wishes to improve the reproducibility of the work that we publish. This form provides structure for consistency and transparency in reporting. For further information on Nature Portfolio policies, see our Editorial Policies and the Editorial Policy Checklist.

Statistics

For all statistical analyses, confirm that the following items are present in the figure legend, table legend, main text, or Methods section.

n/a	Confirmed	
☐	☒	The exact sample size (*n*) for each experimental group/condition, given as a discrete number and unit of measurement
☐	☒	A statement on whether measurements were taken from distinct samples or whether the same sample was measured repeatedly
☐	☒	The statistical test(s) used AND whether they are one- or two-sided<br>*Only common tests should be described solely by name; describe more complex techniques in the Methods section.*
☐	☒	A description of all covariates tested
☒	☐	A description of any assumptions or corrections, such as tests of normality and adjustment for multiple comparisons
☐	☒	A full description of the statistical parameters including central tendency (e.g. means) or other basic estimates (e.g. regression coefficient) AND variation (e.g. standard deviation) or associated estimates of uncertainty (e.g. confidence intervals)
☒	☐	For null hypothesis testing, the test statistic (e.g. *F*, *t*, *r*) with confidence intervals, effect sizes, degrees of freedom and *P* value noted<br>*Give P values as exact values whenever suitable.*
☒	☐	For Bayesian analysis, information on the choice of priors and Markov chain Monte Carlo settings
☒	☐	For hierarchical and complex designs, identification of the appropriate level for tests and full reporting of outcomes
☒	☐	Estimates of effect sizes (e.g. Cohen's *d*, Pearson's *r*), indicating how they were calculated

Our web collection on statistics for biologists contains articles on many of the points above.

Software and code

Policy information about availability of computer code

Data collection	Excel,txt, python
Data analysis	Excel, Origin 2026, python

For manuscripts utilizing custom algorithms or software that are central to the research but not yet described in published literature, software must be made available to editors and reviewers. We strongly encourage code deposition in a community repository (e.g. GitHub). See the Nature Portfolio guidelines for submitting code & software for further information.

Data

Policy information about availability of data

All manuscripts must include a data availability statement. This statement should provide the following information, where applicable:

- Accession codes, unique identifiers, or web links for publicly available datasets
- A description of any restrictions on data availability
- For clinical datasets or third party data, please ensure that the statement adheres to our policy

The data supporting the findings of this study are reported in the main text or the Supplementary Information. Source data are provided with this paper.

Research involving human participants, their data, or biological material

Policy information about studies with human participants or human data. See also policy information about sex, gender (identity/presentation), and sexual orientation and race, ethnicity and racism.

Reporting on sex and gender	No applicable.
Reporting on race, ethnicity, or other socially relevant groupings	No applicable.
Population characteristics	No applicable.
Recruitment	No applicable.
Ethics oversight	No applicable.

Note that full information on the approval of the study protocol must also be provided in the manuscript.

Field-specific reporting

Please select the one below that is the best fit for your research. If you are not sure, read the appropriate sections before making your selection.

☐ Life sciences ☐ Behavioural & social sciences ☒ Ecological, evolutionary & environmental sciences

For a reference copy of the document with all sections, see nature.com/documents/nr-reporting-summary-flat.pdf

Ecological, evolutionary & environmental sciences study design

All studies must disclose on these points even when the disclosure is negative.

Study description	This study reports the synthesis and characterization of Pt single-atom catalysts for nitrogen dioxide detection, encompassing an evaluation of material structural evolution and gas-sensing performances.
Research sample	At least five independent experiments were conducted ($n \geq 5$). Due to the consistency of the results across trials, representative data are presented herein.
Sampling strategy	To ensure the reproducibility and reliability of the sensor performance, at least four sensing cycles were conducted for each individual sensor under identical conditions ($n \geq 4$). The sample size was predetermined based on the high consistency observed across replicates, ensuring that the data are sufficient to validate the stability and reliability of the gas-sensing results.
Data collection	Gas sensing measurements were performed in a custom-built test chamber . Nitrogen dioxide (NO2) and other gases were introduced at defined levels, and sensor responses were recorded in real time using a Source meter. Data were recorded by the authors using a High-precision multi-channel sensor testing system (model: EVM-HP-MC02) together with its dedicated software program. The instrument automatically logged all measurement data.
Timing and spatial scale	All experiments were done in the laboratory from 2023/10 to 2026/01
Data exclusions	No data were excluded from this study.
Reproducibility	Experiments were repeated independently on four distinct sensors to confirm reproducibility. Consistent results were observed across all replicates.
Randomization	Sensor fabrication and testing were conducted under controlled procedures without randomization.
Blinding	Data collection and analysis were performed without blinding.

Did the study involve field work? ☐ Yes ☒ No

Reporting for specific materials, systems and methods

We require information from authors about some types of materials, experimental systems and methods used in many studies. Here, indicate whether each material, system or method listed is relevant to your study. If you are not sure if a list item applies to your research, read the appropriate section before selecting a response.

Materials & experimental systems

n/a	Involved in the study
☒	☐ Antibodies
☒	☐ Eukaryotic cell lines
☒	☐ Palaeontology and archaeology
☒	☐ Animals and other organisms
☒	☐ Clinical data
☒	☐ Dual use research of concern
☒	☐ Plants

Methods

n/a	Involved in the study
☒	☐ ChIP-seq
☒	☐ Flow cytometry
☒	☐ MRI-based neuroimaging

Plants

Seed stocks	No applicable.
Novel plant genotypes	No applicable.
Authentication	No applicable.
