# Supplementary material for: Creating locally enriched single-atom catalysts via bio-inspired internalization process for interference-resistant NO2 detection in complex environments
Source: Nat Commun. 2026 Aug 20;17:9949. doi: 10.1038/s41467-026-76850-4 (PMC13586179; doi:10.1038/s41467-026-76850-4)
Supplement: Supplementary file 4 [file 41467_2026_76850_MOESM5_ESM.pdf]

nature portfolio

Peer Review File

Bio-inspired internalization process for robust gas-sensing system

Corresponding Author: Dr Zhenggang Xue

This file contains all reviewer reports in order by version, followed by all author rebuttals in order by version.

Attachments originally included by the reviewers as part of their assessment can be found at the end of this file.

Version 0:

Reviewer comments:

Reviewer #1

(Remarks to the Author)
Accurate NO_2 detection in complex atmospheres is critical for public safety but is hindered by gas interactions that cause sensor interference or poisoning. This research presents a bio-inspired atomic internalization process that creates concentrated single Pt species on SnO_2 surfaces. This design alters NO_2 molecular distribution, enabling highly selective, interference-resistant detection with over 550 days of stability, surpassing commercial devices. The integrated sensor arrays achieve 100% classification accuracy for single and mixed gases at ultralow cost. This work enables accurate analysis of complex gas mixtures. This research is therefore suggested to be published in Nature Communications after a minor revision.
The concerned points are listed as follows:
1. The detectable NO_2 concentration range is narrow, spanning from 3 ppm to 15 ppm. Please extend the detectable NO_2 concentration range. In this case, the NO_2-responsivity correlation shall be analyzed if it is nonlinear.
2. The temperature-dependent sensitivity to NO_2 should be discussed, by which the operation temperature can be optimized in terms of sensitivity and power consumption.
3. Please elaborate the definition about the loading on Pt_3Sn alloys, and add characterizations related to the loading feature considering that this characteristic is closely correlated to the sensing performance.

Reviewer #2

(Remarks to the Author)

Reviewer #3

(Remarks to the Author)

The authors demonstrate a sophisticated internalization process for synthesizing high-density single Pt species with segregation behaviors, which provides a valuable advancement in the field of single-atom catalysts. The resulting catalysts show favorable NO_2 sensing performance, good long-term stability, and strong anti-interference capability, and they perform comparably well to or slightly better than commercial NO_2 sensors, indicating considerable practical applicability. Furthermore, the sensing system integrated with robotics can be applied to real-world scenarios, representing a meaningful research achievement. A suite of complementary methods, in situ characterizations, and AIMD simulations support the controlled design of single-atom catalysts and their potential applications in emerging sensing fields. Based on the topic and its potential impact, I consider this manuscript suitable for publication in Nature Communications after minor revisions to address the following concerns.
1. Please explain in detail the necessity of testing the sensing performance of the materials at different temperatures (Fig. 4), as well as the specific impact of temperature on the catalytic performance.
2. For the Pt-Pt and Pt-O peaks in Fig. 3f, the authors are advised to provide relevant references or a detailed explanation to clarify their assignment and significance.
3. In Fig. 4a, it is unclear whether the figure presents a comparison of the sensor's resistance signal or another type of

signal. Additionally, the relationship between color vividness and sensing performance requires further clarification—specifically, whether a more vivid color indicates better sensing performance. It is recommended that the authors provide a detailed explanation.
4. In the "Assessment of sensor arrays" section, the authors are encouraged to provide a more detailed description of the experimental procedure for testing interfering gases (e.g., H_2S and SO_2), including parameters such as gas concentration, exposure time, and testing environment.
5. On Page 6, the authors synthesized a series of nanofibers via electrospinning technology and stated: "As seen in Figs. S2 and S3, the fibers exhibit average diameters of approximately 200 nm. It is recommended that the authors clarify whether all nanofibers have diameters around 200 nm. Furthermore, an explanation is needed for why different Pt_3Sn doping concentrations do not alter the fiber morphology."

Version 1:

Reviewer comments:

Reviewer #1

(Remarks to the Author)
After careful review of the author's response, it is concluded that the manuscript has significantly improved and meets the publication requirements.

Reviewer #3

(Remarks to the Author)
The authors have addressed all the reviewers' concerns. I recommend acceptance of this manuscript.

Open Access This Peer Review File is licensed under a Creative Commons Attribution 4.0 International License, which permits use, sharing, adaptation, distribution and reproduction in any medium or format, as long as you give appropriate credit to the original author(s) and the source, provide a link to the Creative Commons license, and indicate if changes were made.
In cases where reviewers are anonymous, credit should be given to 'Anonymous Referee' and the source.
The images or other third party material in this Peer Review File are included in the article’s Creative Commons license, unless indicated otherwise in a credit line to the material. If material is not included in the article’s Creative Commons license and your intended use is not permitted by statutory regulation or exceeds the permitted use, you will need to obtain permission directly from the copyright holder.
To view a copy of this license, visit https://creativecommons.org/licenses/by/4.0/

Point-to-point responses to reviewers' comments

We sincerely thank the reviewers for their constructive suggestions. Below, we provide point-by-point responses to all comments and questions raised, and the corresponding revisions are highlighted in yellow in the revised manuscript and Supporting Information.

Reviewer 1:

Accurate NO_2 detection in complex atmospheres is critical for public safety but is hindered by gas interactions that cause sensor interference or poisoning. This research presents a bio-inspired atomic internalization process that creates concentrated single Pt species on SnO_2 surfaces. This design alters NO_2 molecular distribution, enabling highly selective, interference-resistant detection with over 550 days of stability, surpassing commercial devices. The integrated sensor arrays achieve 100% classification accuracy for single and mixed gases at ultralow cost. This work enables accurate analysis of complex gas mixtures. This research is therefore suggested to be published in Nature Communications after a minor revision. The concerned points are listed as follows:

Reply: We appreciate the reviewer's careful evaluation of our work. Following the reviewer's suggestion, we have conducted additional experiments, including an extension of the NO_2 detection range, and expanded the discussion to address the concerns raised. We thank the reviewer for the positive feedback and constructive comments, which have been valuable in helping us improve our manuscript.

Comments 1: The detectable NO_2 concentration range is narrow, spanning from 3 ppm to 15 ppm. Please extend the detectable NO_2 concentration range. In this case, the NO_2-responsivity correlation shall be analyzed if it is nonlinear.

Reply: We sincerely appreciate the reviewer's insightful suggestion to evaluate the performances of sensors across a wider concentration range and to analyze the response-concentration relationship. This is crucial for assessing its practical detection capability. Following your advice, we have conducted the following revisions and supplementary experiments.

In the original manuscript, the detectable NO_2 concentration range for the 1%-PtSn-SnO_2 sample is between 0.05 ppm and 20 ppm. Considering that NO_2 is a highly hazardous gas where even trace amounts can pose significant risks, accurate and interference-resistant detection at low concentrations is essential in numerous application scenarios. Therefore, extensive literature reports that NO_2 gas-sensing materials typically demonstrate a detection range below 10 ppm (*ACS Nano*, **36**, 32732 (2025), *Adv. Mater.*, **35**, 2207199 (2022), *Materials Science & Engineering R*, **160** 100819 (2024)). Accordingly, we have significantly expanded the tested NO_2 concentration range and the complete dataset is presented in the Fig. R1(a, b). The relationship of responses to NO_2 concentration was meticulously analyzed. As shown in the Fig. R1(a, b), the relationship exhibits a clear dual-regime behavior:

1. In the low-concentration region (0.05-20 ppm), the response follows a linear relationship (y=1.14+2.88x). This linearity is paramount for accurate concentration quantification in practical applications.

2. At higher concentrations (20-30 ppm), the response gradually transitions into a sub-linear trend, which can be well described by a power-law model ($y=36.8-171.4*0.86^x$). This is a classic signature of progressive saturation of surface adsorption sites (e.g., *Adv. Mater.*, **37**, 2507829 (2025), *Adv. Funct. Mater.*, **31**, 2102439 (2021), *Sens. Actuator B-Chem.*, **44,** 139015 (2026)). The maintained high response even at 30 ppm underscores the robustness of the sensing material.

We have incorporated this new data, analysis, and discussion into the revised supporting information (Fig. S33(d, e), **Page 49**). Thank you for prompting this significant

improvement to our work.

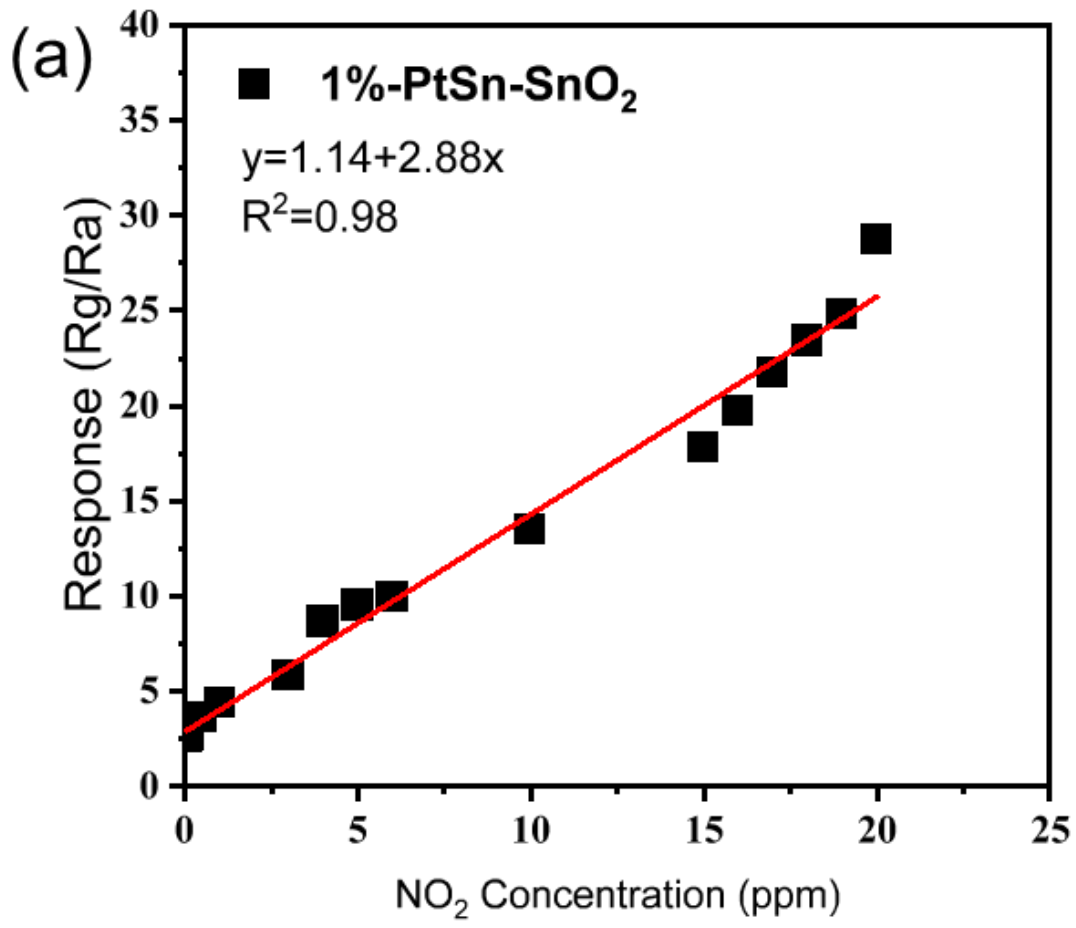

Fig. R1 (a) at low concentration of NO_2 gas (0.05-20 ppm) and (b) high concentration of NO_2 gas (15-30 ppm).

Comments 2: The temperature-dependent sensitivity to NO_2 should be discussed, by which the operation temperature can be optimized in terms of sensitivity and power consumption.

Reply: We sincerely thank you for your thorough review and valuable comments. Traditionally, the dependence of sensor sensitivity on the operating temperature is a classic and well-established phenomenon in the field of gas-sensing. This is mainly because at low temperatures, the response is limited by insufficient thermal energy to overcome the activation barrier for surface reactions. As the temperature increases, the reaction rate accelerates, leading to higher sensitivity. However, beyond the optimal temperature, the thermal desorption of NO_2 molecules becomes dominant over adsorption, resulting in a decline in response. This behavior is consistent with the classical theory of chemisorption on semiconductor surfaces. Therefore, in this work, the responses of the eight fabricated sensors to 5 ppm NO_2 show a typical volcanic trend (rising first and then falling) in Fig. S29 (Supporting Information). Typically, the operating temperature of sensor is generally defined as the temperature yielding the

highest sensitivity.

Furthermore, highly active noble metals have been demonstrated to significantly lower the optimal operating temperature of semiconducting sensing materials through mechanisms such as electronic sensitization and chemical sensitization. Specifically, the optimal operating temperatures for SnO_2, Pt NPs-SnO_2, Pt_1-SnO_2, 0.3%-PtSn-SnO_2, 0.6%-PtSn-SnO_2, 1%-PtSn-SnO_2, 1.5%-PtSn-SnO_2, 2%-PtSn-SnO_2 sensors were determined to be 150 °C, 130 °C, 130 °C, 90 °C, 90 °C, 90 °C, 90 °C, and 90 °C, respectively. This confirms that the aggregation-dispersed Pt species possess high catalytic activity. Moreover, the lower operating temperature of the sensor enables low-power consumption sensing, with its power consumption reduced to 6 mW as shown in Fig. S30.

The relevant supplementary discussion and the rationale for selecting the operating temperatures for different sensors have been added in **Page 45** of the Supporting Information: "The gas-sensing reaction in semiconductors is essentially a surface catalytic redox process. At low temperatures, gas molecules lack sufficient activation energy, resulting in sluggish surface reaction kinetics and weak sensing signals. Conversely, at excessively high temperatures, although the chemical reaction rate accelerates, the desorption rate of gas molecules surpasses their adsorption rate, leading to a decline in response. The peak response is achieved only at the optimal working temperature, where adsorption and reaction reach a dynamic equilibrium. Considering the interplay between sensitivity and power consumption, the operating temperature for each sensor was defined as the point of maximum sensitivity."

Comments 3: Please elaborate the definition about the loading on Pt_3Sn alloys, and add characterizations related to the loading feature considering that this characteristic is closely correlated to the sensing performance.

Reply: Thanks for the reviewer's kind comments. We apologize for any confusion caused by the lack of detail in the Methods Section. In our work, we first prepared the precursor by mixing the Pt_3Sn alloy with the tin precursor. Subsequently, a series of PtSn-Sn NFs samples were fabricated via electrospinning technology. The loading of the alloy was initially calculated based on the mass ratio of Pt in the Pt_3Sn alloy to Sn in the whole precursors. In detailed, the materials obtained by electrospinning 1.2 g of the $SnCl_2 \cdot 2H_2O$ precursor and 2.5 mg, 5 mg, 8 mg, 12 mg, and 16 mg of Pt_3Sn alloy precursors were designated as 0.3%-PtSn-Sn NFs, 0.6%-PtSn-Sn NFs, 1%-PtSn-Sn NFs,1.5%-PtSn-Sn NFs and 2.0%-PtSn-Sn NFs, respectively. Upon pyrolysis at 900 °C in air, the PtSn-Sn NFs samples yielded a series of final products, which were designated as the PtSn-SnO_2 series. We are fully aware that the Pt mass ratio indicated in the sample names does not equate to the actual Pt loading in the final products after calcination in air. Therefore, to obtain precise measurements, we conducted ICP-OES analysis on the entire series of PtSn-SnO_2 samples to accurately determine the mass fractions of Pt. The comprehensive ICP-OES results are summarized in Table S1 of the Supporting Information. The measured actual Pt contents in the 0.3%-PtSn-SnO_2, 0.6%-PtSn-SnO_2, 1%-PtSn-SnO_2, 1.5%-PtSn-SnO_2 and 2.0%-PtSn-SnO_2 samples are 0.10 wt.%, 0.52 wt.%, 0.72 wt.%, 1.1 wt.% and 1.5 wt.%, respectively.

Accordingly, we have added the following discussion to the manuscript in the section Methods in the **Page 26**: "The loading of the alloy was initially calculated based on the mass ratio of Pt in the Pt_3Sn alloy to Sn in the tin precursor."

Reviewer 2:

After careful reading, although the manuscript presents extensive data on the sensing properties and application, the analysis of the chemical and physical properties of SnO_2-based materials' single-atom modification is insufficient. Furthermore, based solely on the explanations provided in the manuscript, the relationship between these mechanistic descriptions and the adsorption theory used to interpret the concentration dependence

of the gas response is inconsistent. To ensure the quality and integrity of this review, I would like to express the following opinions:

Reply: We appreciate reviewer's careful reading of our manuscript, enlightening comments and valuable suggestions. We have answered all questions from you. Based on reviewer's suggestions, we have added related explanations in the revised manuscript to improve the quality and readability. The specific replies and modifications are listed as follows:

Comments 1: Please add standard reference patterns for SnO_2 and Pt_3Sn to Fig S7 by marking vertical lines at the bottom of the plot. Include the corresponding standard PDF card numbers in the figure caption.

Reply: Thank you for your kind reminder. Following your advice, we have added the standard reference patterns for SnO_2 (JCPDS No. 41-1445) in black and Pt_3Sn (JCPDS No. 35-1360) in red to Fig. S7. As seen in Fig. R2, vertical lines have been plotted at the bottom of the figure to mark the positions of characteristic diffraction peaks. The corresponding PDF card numbers have been included in the revised figure caption. These additions significantly improve the clarity of the phase identification.

The revised figure can be found on **Page 23** of the Supporting Information.

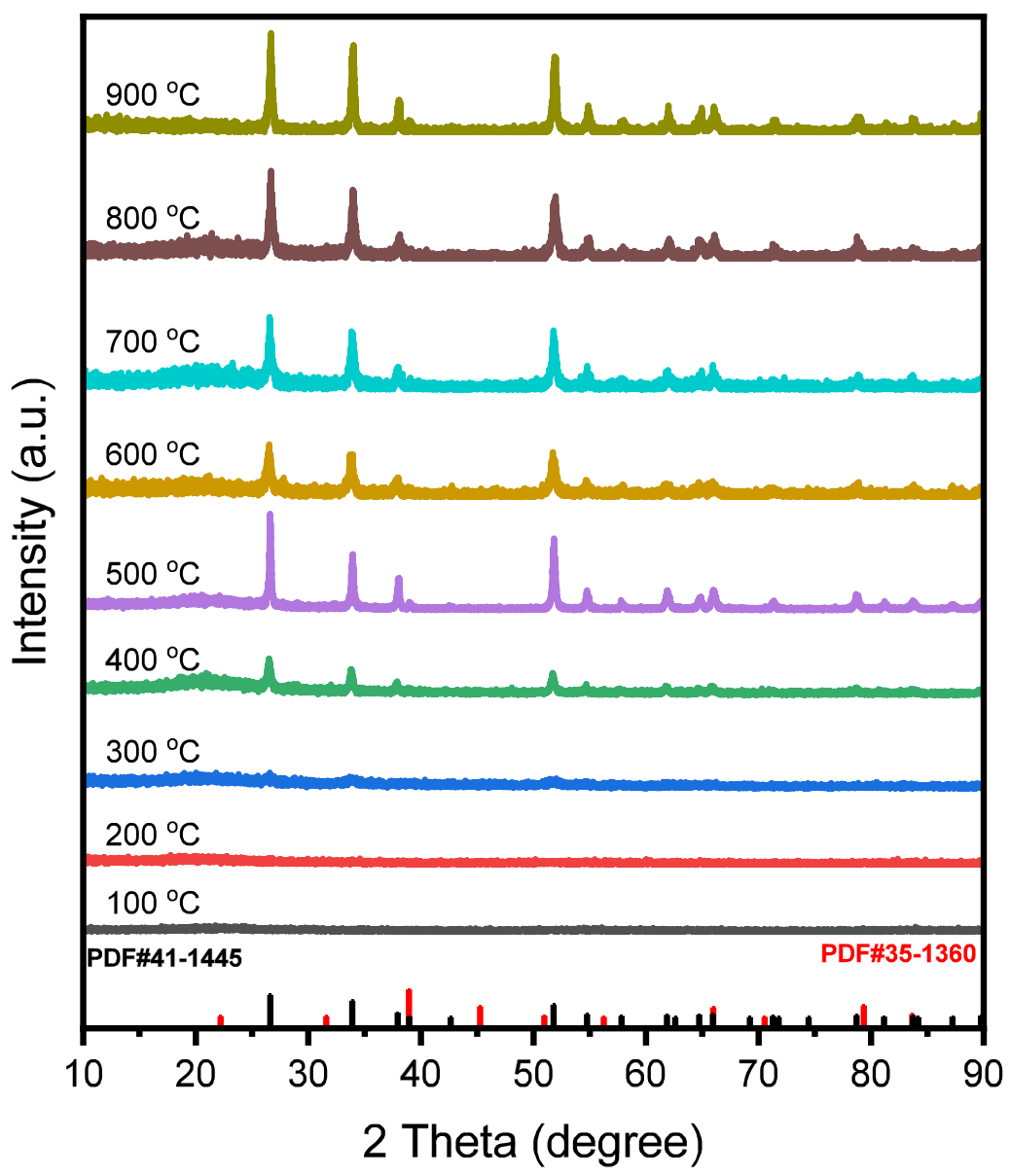

Fig. R2 XRD spectra of 1%PtSn-Sn NFs after different temperature treatment. (JCPDS No. 41-1445: SnO_2 and JCPDS No. 35-1360: Pt_3Sn)

Comments 2: The manuscript claims that Pt species agglomerate into nanoparticles when the Pt_3Sn loading exceeds 1.5 wt.% based on Supplementary Fig. S12-S15 Please add quantitative particle size analysis to Fig. S12-S15 and revise the original description with quantitative statistical results.

Reply: We sincerely appreciate the reviewer's valuable suggestion. Following your advice, we have added annotations regarding the size of Pt nanoparticles to the EDS-TEM results in Fig. S15 (now renumbered as Fig. R3 in the comments to reviewers). Furthermore, based on additional TEM characterization images of the 1.5%-PtSn-SnO_2 and 2%-PtSn-SnO_2 samples, we have conducted a statistical analysis of the Pt particle size distribution.

We have performed precise measurements on the individually resolvable Pt nanoparticles present in the EDS-mapping images (Fig.R3(a, b)). The measured diameters of these specific particles are now directly annotated on the corresponding images in the Fig. R3 ("~30 nm", "~50 nm", "~60 nm"). The figure captions for the

relevant supplementary figures have been updated to state that the size annotations of the Pt particles. Furthermore, a statistical analysis of the Pt particle sizes in the samples was performed. As shown in Fig. R3(c), the size distribution of the Pt nanoparticles is presented. When the loading of the Pt_3Sn alloys exceeds 1.5 wt.%, the Pt species aggregate to form nanoparticles with sizes approximately ranging from 30 to 60 nm, with the highest frequency of occurrence around 50 nm.

The supporting information (Page 31) has been revised to incorporate this data while transparently stating the observational basis: "EDS-mapping results of 1.5%-PtSn-SnO_2 (Fig.S15(a)) and 2%-PtSn-SnO_2 (Fig.S15(b)) show that distinct Pt particles on the surfaces of samples. The particle size distribution histogram of Pt (Fig. S15(c)) indicates that when the loading of the Pt_3Sn alloys exceeds 1.5 wt.%, the Pt species aggregate to form Pt nanoparticles with diameters approximately ranging from 30 to 60 nm." Furthermore, we have added an explanation regarding on **Page 9** of the manuscription "Furthermore, Pt_3Sn alloys with different loadings (including 0.3%-PtSn-SnO_2, 0.6%-PtSn-SnO_2, 1.0%-PtSn-SnO_2, 1.5%-PtSn-SnO_2, 2.0%-PtSn-SnO_2) exhibit distinct agglomeration of Pt species into nanoparticles (30-60 nm) when the loading exceeds 1.5% (Fig. S12-S15)."

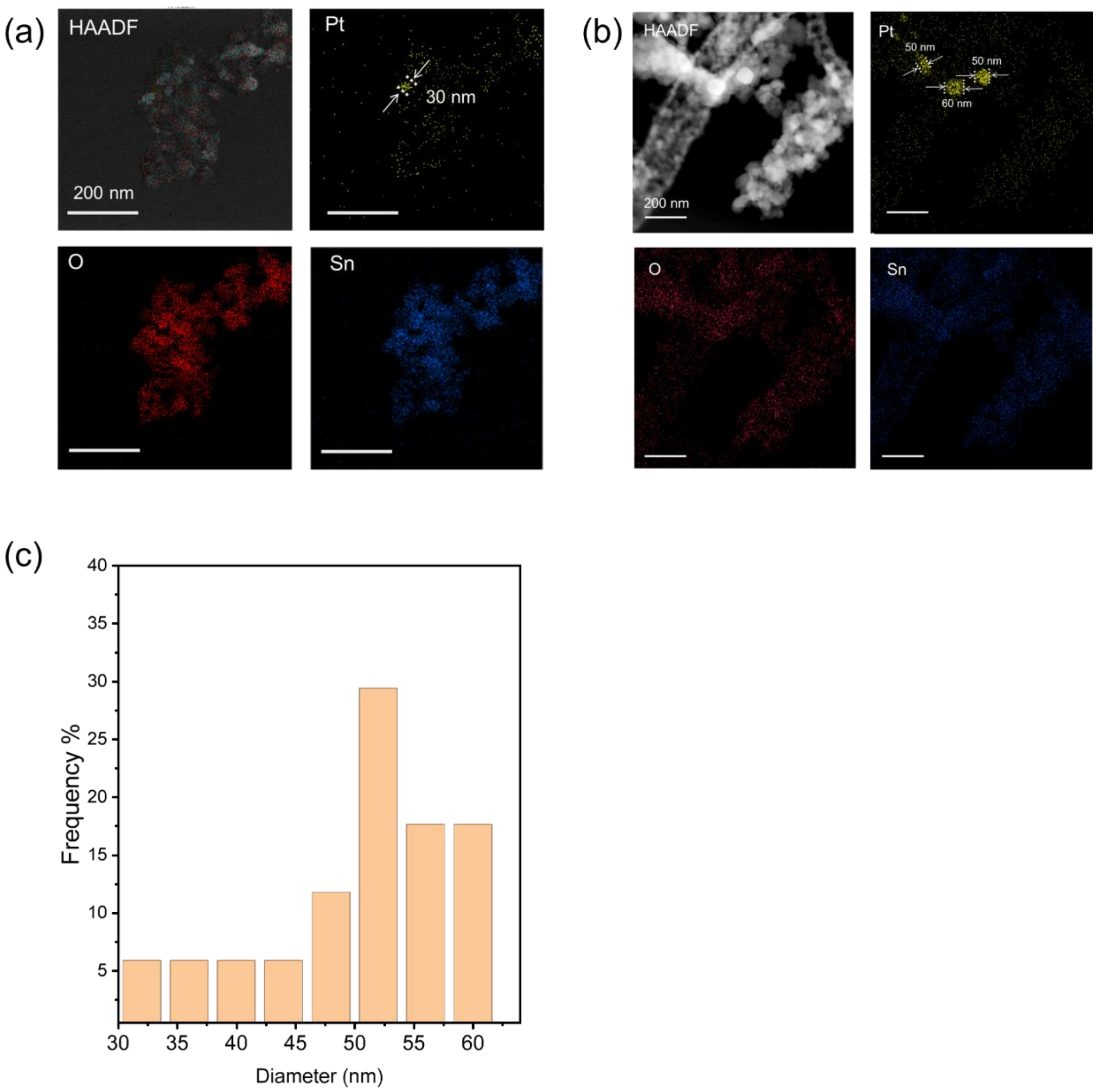

Fig. R3 EDS-mapping images of (a) 1.5%-PtSn-SnO_2 and (b) 2%-PtSn-SnO_2. (c) Particle size distribution of 1.5%-PtSn-SnO_2 and 2%-PtSn-SnO_2 samples.

Comments 3: Please add the label "(b)" to the 3D fitting plot in Fig. S18 to match the figure caption description and maintain consistent formatting with other figures.

Reply: We thank the referee for the careful reading and kinder reminder. We have revised Fig. S18 by adding the panel label **(b)**. The corrected figure is presented as Fig. S18 on **Page 34** of the Supporting Information, as shown in Fig. R4.

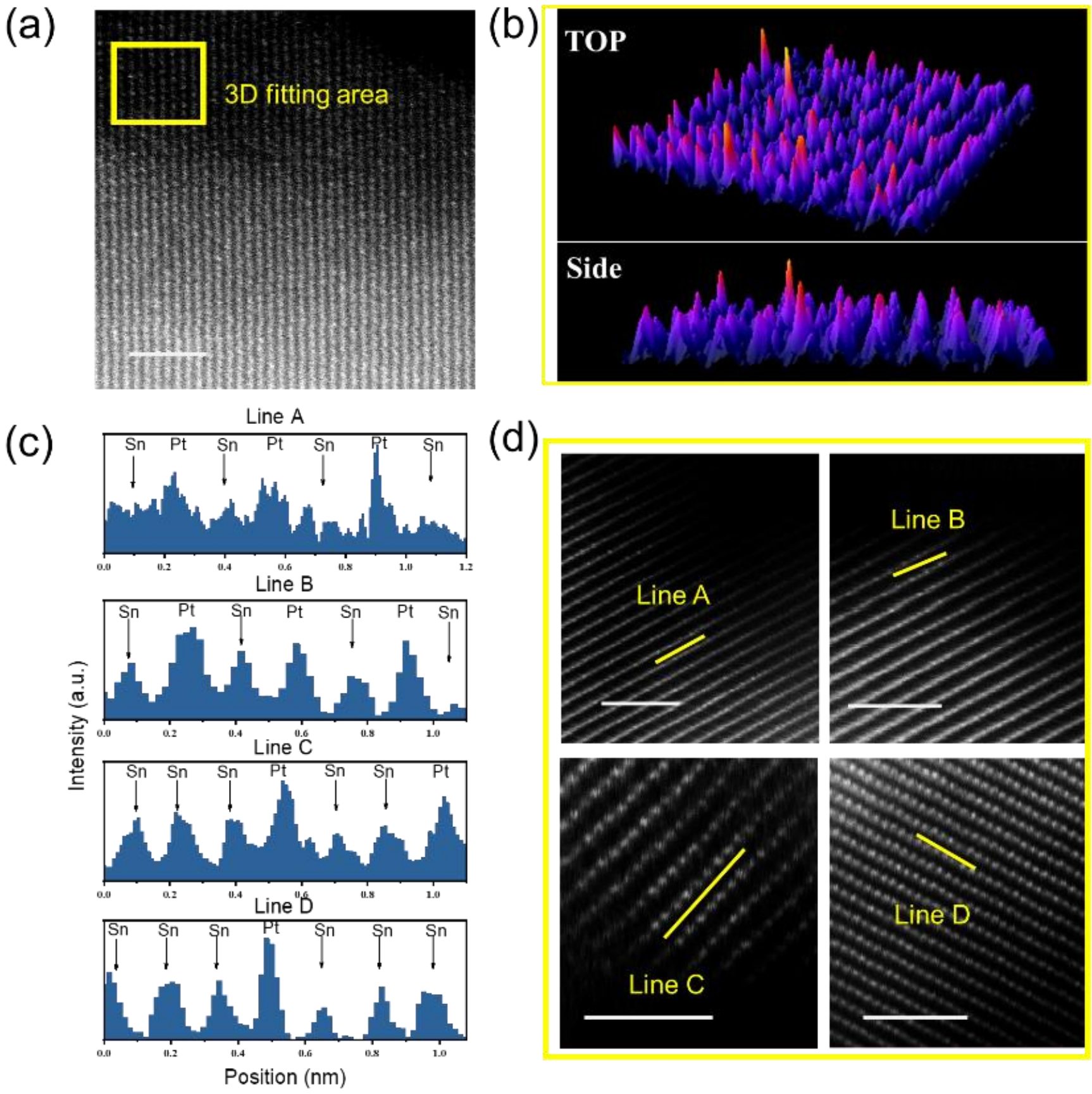

Fig. R4 (a) HAADF-STEM image of 1%-PtSn-SnO_2. (b) The atom-overlapping Gaussian-function fitting images of selective area scan (measured from white frame of (a)). (c) The intensity profiles obtained in regions X-Y in (d). (d) Other four representative HAADF images of 1%-PtSn-SnO_2.

Comments 4: There is a grammatical error in the sentence "promotes the formation of Pt NPs This is conformed to the mapping results in Fig. S15". A period is missing between "Pt NPs" and "This", leading to a run-on sentence.

Reply: We thank the referee for kinder reminder. We regret the editorial error in the original manuscript. We have corrected the punctuation by adding a period between "Pt NPs" and "This". The revised sentence "The FT-EXAFS spectra of the Pt L_3-edge reveal only Pt-O bonds in the 0.3%-PtSn-SnO_2, 0.6%-PtSn-SnO_2, and 1%-PtSn-SnO_2 samples (Fig. 3f), while both Pt-Pt and Pt-O bonds exist in the 1.5%-PtSn-SnO_2, 2%-

$PtSn-SnO_2$ samples, indicating that excessive Pt_3Sn alloy promotes the formation of Pt NPs. This is conformed to the mapping results in Fig. S15." are located on **Page 11** in the manuscription.

Comments 5: Revise the caption of Figure S31 by changing "towards 10 ppm NO_2 gas" to "towards different concentrations of NO_2 gas (0.1-5 ppm)" to be consistent with the data in the figure.

Reply: We sincerely thank the reviewer for pointing out this inaccuracy. We apologize for this oversight and have revised the caption of Figure S31 in the Supporting Information **Page 47** to: "Responses of the SnO_2, Pt NPs- SnO_2, Pt_1-SnO_2, 0.3%-PtSn-SnO_2, 0.6%-PtSn-SnO_2, 1%-PtSn-SnO_2, 1.5%-PtSn-SnO_2, 2%-PtSn-SnO_2 sensors towards 0.05-5 ppm NO_2 gas". This revision more accurately reflects. We sincerely thank you once again for your careful and thorough review.

Comments 6: Please label the optimal operating temperature for each sensor and the relative humidity during testing in Fig. 4b.

Reply: We are grateful to your valuable comments. Following your advice, we have updated the figure to explicitly label the optimal operating temperature (T) for each sensor and the relative humidity (RH) during testing. The specific temperature values of SnO_2, Pt NPs-SnO_2, Pt_1-SnO_2, 0.3%-PtSn-SnO_2, 0.6%-PtSn-SnO_2, 1%-PtSn-SnO_2, 1.5%-PtSn-SnO_2, 2%-PtSn-SnO_2 sensors are 150 ºC, 130 ºC, 130 ºC, 90 ºC, 90 ºC, 90 ºC, 90 ºC and 90 ºC, respectively. And the testing humidity condition (30% RH) has also been added to the Fig. R5.

The revised figure can be found in Fig.4b on **Page 17** of the manuscription.

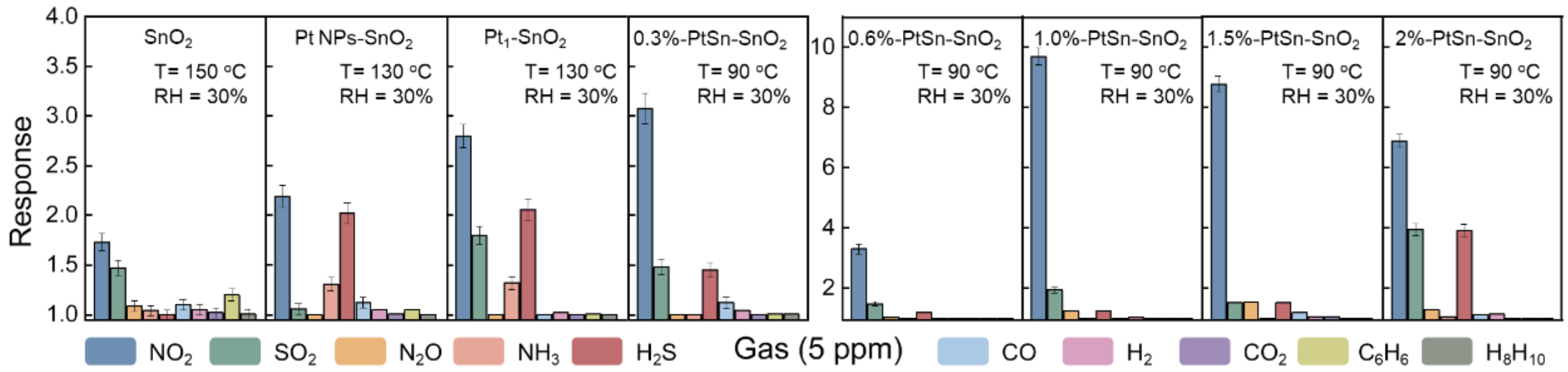

Fig. R5 The response values of the eight sensors to the ten different gases under optimal working temperature.

Comments 7: The anti-interference test only includes SO_2, H_2S, and NH_3, which does not cover common interfering gases in practical environments such as CO_2. To better evaluate the practical applicability and selectivity of the sensors, the anti-interference performance should be further verified against these common interfering gases.

Reply: We thank the referee for such enlightening comments. According to the reviewer's comments, the interfering gases in practical environments such as CO_2, was further tested. As seen in Fig.R6, the responses of the 1%-PtSn-SnO_2 samples to 5 ppm NO_2 show no significant fluctuation with the co-introduction of 1-10 ppm CO_2 as an interferent. For MOS-based semiconductor sensors, the evaluation of gas-sensing performance is typically conducted under an ambient air atmosphere and the CO_2 is generally inert over sensing surfaces. Consequently, MOS-based semiconductor sensors specifically for CO_2 detection are rarely reported.

In addition to SO_2, H_2S and NH_3 discussed in the manuscript, we systematically evaluated the sensor's response to a panel of common and relevant interfering gases. As the reviewer correctly suggests, this panel includes CO_2, as well as other significant interferents such as N_2O, CO, H_2, C_6H_6 (Benzene) and C_8H_{10} (Xylene). These gases comprehensively encompass most potential interfering species encountered in real-world application scenarios. The complete dataset for all these gases is consolidated and presented in Supplementary Fig.S53. The results in Fig. R7 demonstrate that the

1%-PtSn-SnO_2 sensor (Sensor 6/Sensor 7) exhibits negligible responses to all other tested interfering gases, including CO_2. Its excellent selectivity in complex atmospheres, as evidenced by the minimal cross-response, ensures high practical applicability, which is firmly grounded in our experimental data.

We apologize for any initial lack of clarity regarding the full extent of our anti-interference tests. And incorporate the following explanation into the manuscription at Line 3, **Page 19**: "Additional tests involving cross-mixed gas concentrations and complex atmospheres further confirm the robust anti-interference capability and stability of Sensors 6 and 7 (Fig. S50-S53)." We believe the provided data in Fig. S53 offers a solid foundation for assessing the sensor's practical suitability.

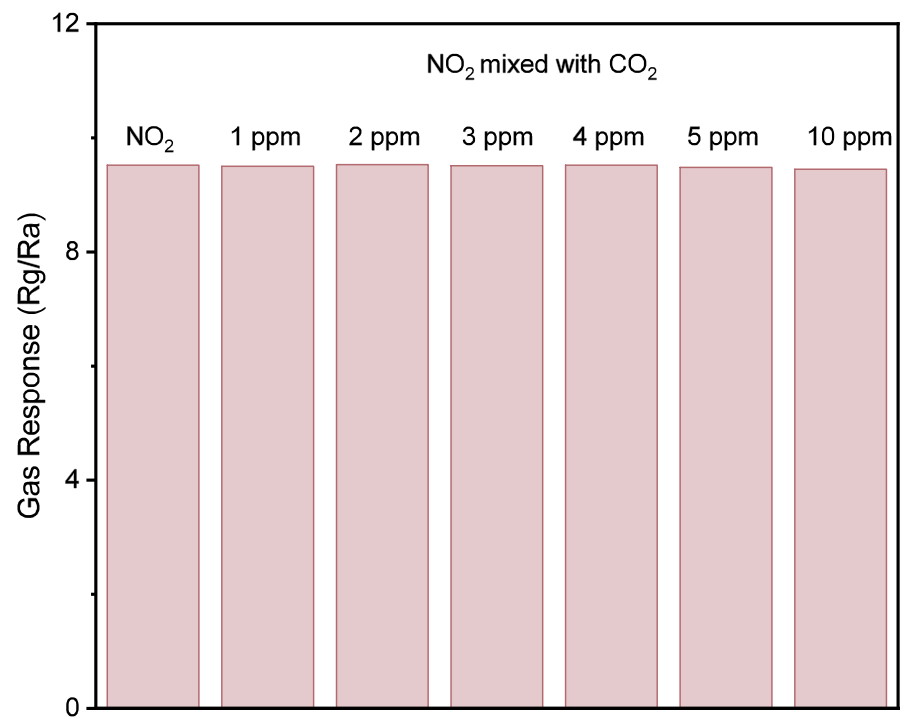

Fig. R6 Gas responses of sensors based 1%-PtSn-SnO_2 of 5 ppm NO_2 gas mixing with 1-10 ppm of CO_2.

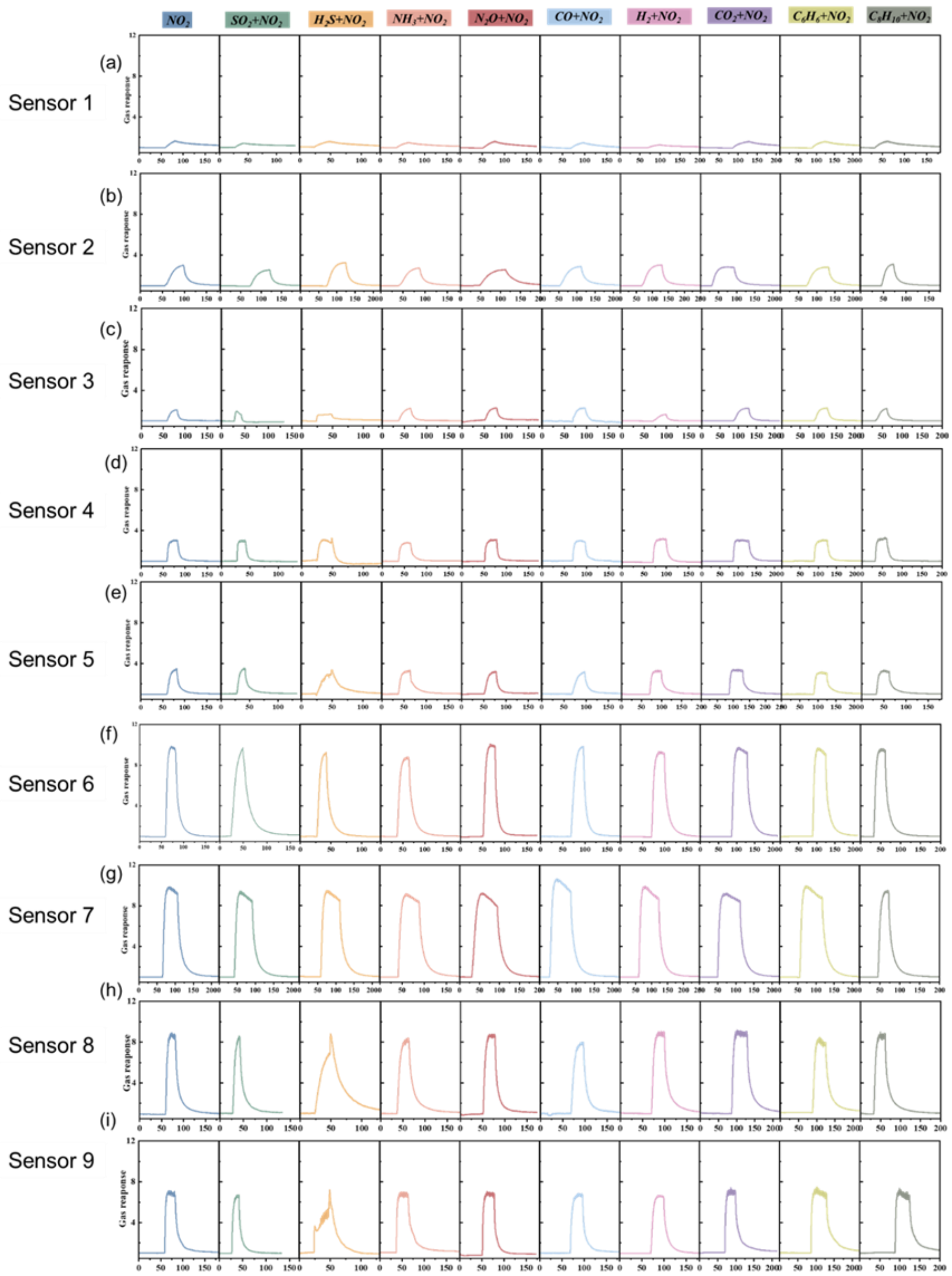

Fig. R7 Dynamic gas-sensing transients of 9 sensors under 5 ppm NO_2 and 5 ppm other gases.

Comments 8: The optimal working temperature of the best-performing sample 1%-PtSn-SnO_2 (90 ºC) is lower than that of conventional SnO_2-based sensors. Please explain the reason from the physicochemical mechanism.

Reply: Thanks for your kind comments. The optimal working temperature of the 1%-PtSn-SnO_2 sample is lowered to 90 ºC primarily due to the introduced by the

introduction of Pt species with a unique dispersion pattern. The underlying physicochemical mechanisms can be explained as follows.

Decoration with noble metals can significantly lower the optimal operating temperature of gas-sensing materials (e.g., *Coord. Chem. Rev.*, **562**, 217998 (2026), *Sens. Actuat. B Chem.*, **239**, 253 (2017), *Nano-Micro Lett.* **14**, 206 (2022)). Furthermore, a series of noble-metal-decorated materials have been reported to operate effectively at temperatures around 100°C, such as *Sens. Actuat. B Chem.*, **375**, 132943 (2023), *Rare Metals*, **40**, 1642 (2021), *Sens. Actuat. B Chem.*, **459**, 139864 (2026). Primarily through a catalytic effect, noble metals reduce the activation energy of gas reactions, provide additional active sites and amplify the responses of sensors via electronic interactions. This enables the sensors to achieve reaction rates and signal intensities at lower temperatures comparable to those of conventional sensors operating at high temperatures. Consequently, the optimal operating temperature is markedly reduced, leading to lower power consumption.

Firstly, the Pt single atoms on regionalized SnO_2 surfaces facilitate enhanced electron transfer between the material surface and NO_2 molecules, thereby amplifying the electronic response signals. This phenomenon is clearly evidenced by in-situ Raman characterization. As shown in Fig. S46 (Supporting Information), the characteristic Raman peak at 621 cm^{-1} of the 1%-PtSn-SnO_2 sample exhibits a significant shift. This pronounced electron transfer effect can occur at a lower operating temperature, consequently reducing the optimal working temperature of the sample to 90 °C. Secondly, through kinetic adsorption-desorption tests of NO_2 gas and AIMD simulation results (Fig. S41-44), we demonstrate that the highly dispersed Pt species on the surface of the 1%-PtSn-SnO_2 sample can alter the adsorption distribution pattern of NO_2 molecules, leading to increased adsorption. Compared to conventional SnO_2, this optimizes the adsorption efficiency of NO_2 and shifts the kinetic equilibrium of the adsorption reaction to a lower temperature.

In summary, the unique dispersion of Pt species in the 1%-PtSn-SnO_2 sample creates a synergistic electronic and catalytic microenvironment. It optimizes NO_2 adsorption,

amplifies the electronic signal per adsorption event and catalytically lowers the energy barrier for the surface reaction. This collective action shifts the balance of adsorption, charge transfer, and reaction kinetics to a lower thermal energy regime, enabling high-sensitivity detection at the significantly reduced optimal operating temperature of 90 °C.

Reviewer 3:

The authors demonstrate a sophisticated internalization process for synthesizing high-density single Pt species with segregation behaviors, which provides a valuable advancement in the field of single-atom catalysts. The resulting catalysts show favorable NO_2 sensing performance, good long-term stability, and strong anti-interference capability, and they perform comparably well to or slightly better than commercial NO_2 sensors, indicating considerable practical applicability. Furthermore, the sensing system integrated with robotics can be applied to real-world scenarios, representing a meaningful research achievement. A suite of complementary methods, in situ characterizations, and AIMD simulations support the controlled design of single-atom catalysts and their potential applications in emerging sensing fields. Based on the topic and its potential impact, I consider this manuscript suitable for publication in Nature Communications after minor revisions to address the following concerns.

Reply: We sincerely appreciate the reviewer's highly positive and encouraging evaluation of our work. We have carefully addressed all concerns and constructive suggestions in a point-by-point manner below, and the corresponding revisions have been highlighted in yellow in the revised manuscript.

Comments 1: Please explain in detail the necessity of testing the sensing performance of the materials at different temperatures (Fig. 4), as well as the specific impact of temperature on the catalytic performance.

Reply: We thank the referee for this valuable comment. Generally, evaluating the gas-sensing performance of a material begins with testing its response across various operating temperatures to identify the **optimal working temperature**. It is only at this specific temperature that the sensors exhibit their highest sensitivity and selectivity. The gas-sensing reaction in semiconductors is essentially a surface catalytic redox process. At low temperatures, gas molecules lack sufficient activation energy, resulting in sluggish surface reaction kinetics and weak sensing signals. Conversely, at excessively high temperatures, although the chemical reaction rate accelerates, the desorption rate of gas molecules surpasses their adsorption rate, leading to a decline in response. The peak response is achieved only at the optimal working temperature, where adsorption and reaction reach a dynamic equilibrium.

As illustrated in Fig. R8, the responses of the four sensors to 5 ppm NO_2 exhibit a typical volcanic trend (initially increasing and subsequently decreasing) as the temperature rises from 70 ºC to 170 ºC. To further elucidate the sensing characteristics, we conducted comprehensive tests on eight types of sensors exposed to ten different gases across varying temperatures. The complete dataset, presented in Fig. 4 in the origin manuscription, provides a holistic overview of the sensing profiles and facilitates the identification of the optimal operating temperatures for each gas analyte.

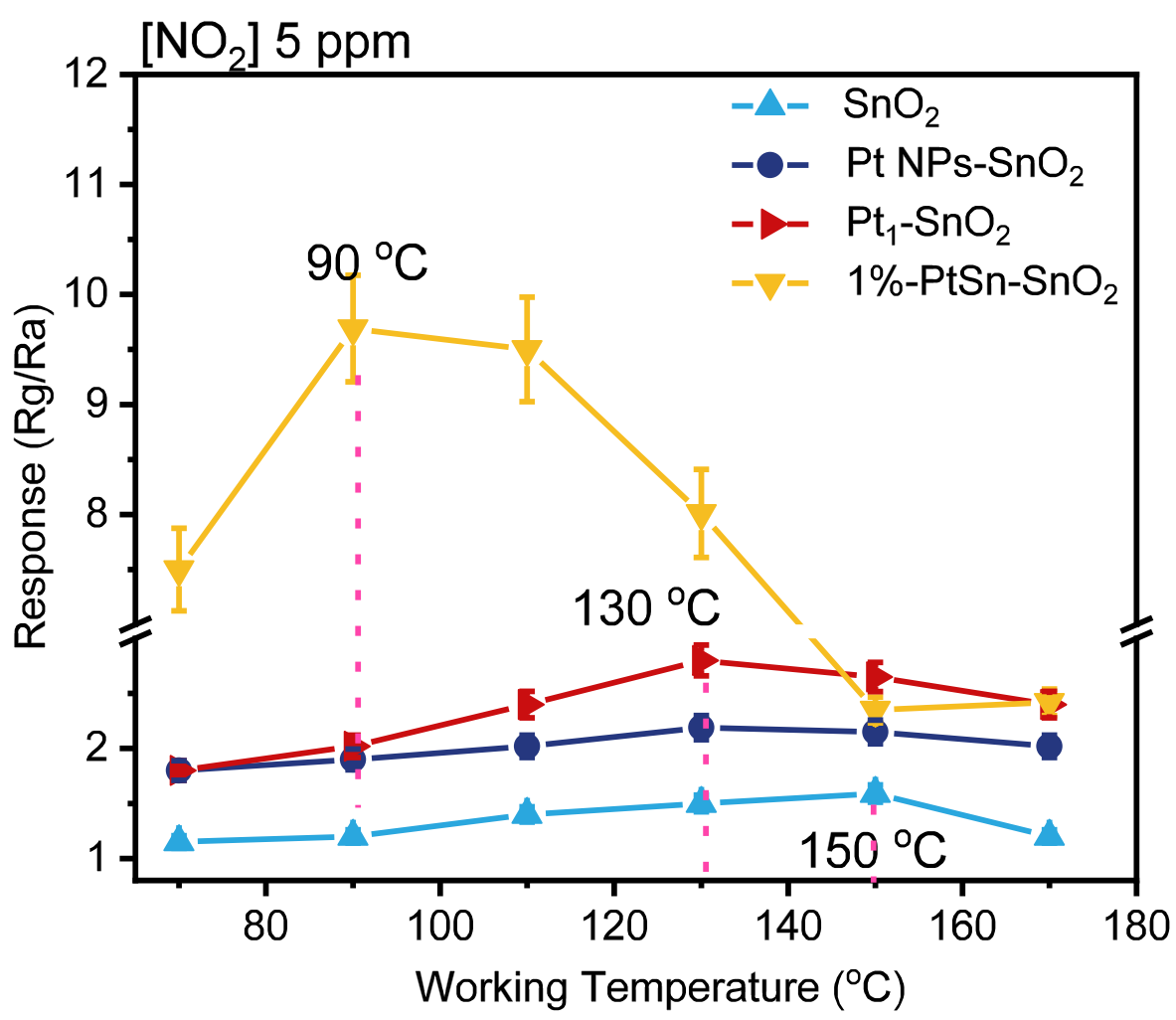

Fig. R8 Responses of SnO_2, Pt NPs-SnO_2, Pt_1-SnO_2, 1%-PtSn-SnO_2 sensors towards 5 ppm NO_2 at different temperature.

Comments 2: For the Pt-Pt and Pt-O peaks in Fig. 3f, the authors are advised to provide relevant references or a detailed explanation to clarify their assignment and significance.

Reply: Thanks for reviewer's professional suggestion. We would like to clarify that the Fourier transform EXAFS (FT-EXAFS) spectrum presented in Fig. 3f is the **raw, phase-uncorrected data**. As shown in Fig. R9, the Fourier transform EXAFS (FT-EXAFS) spectrum of 0.3%-PtSn-SnO_2, 0.3%-PtSn-SnO_2, 0.6%-PtSn-SnO_2, 1%-PtSn-SnO_2, 1.5%-PtSn-SnO_2 and 2%-PtSn-SnO_2 exhibits two prominent peaks.

1. The Pt-O coordination shell (~2.0 Å): The first shell peak at approximately 1.65 Å corresponds to the Pt-O bond. This peak arises from the oxygen atoms in the immediate coordination environment of the Pt atoms, confirming the formation of a Pt-O-Sn interfacial structure (*Science*, **388**,497-502 (2025)).
2. The Pt-Pt coordination shell (~2.75-2.78 Å): The second shell peak at around 2.3 Å is attributed to the Pt-Pt scattering path, which is characteristic of the metallic Pt (Pt^0) phase. The intensity and position of this peak are consistent with the formation of Pt nanoparticles (NPs) or clusters, as supported by *Nature* **643**, 691–698 (2025).

Due to the inherent phase shift of the photoelectron wave, the apparent distances in the uncorrected FT-EXAFS are always shorter than the real atomic distances. For the Pt-Pt coordination shell, although its real bond length is approximately 2.75-2.78 Å, it typically appears at 2.2-2.4 Å (e.g., 2.3 Å in our Fig. 3f) in the uncorrected R-space. Similarly, the Pt-O peak (real bond length ~2.0 Å) usually appears around 1.5-1.7 Å in the uncorrected spectrum.

We have added detailed explanations "The FT-EXAFS spectrum is not corrected for phase shift, therefore the peak positions are shorter than the actual atomic distances." in Page 12 of the manuscription. In addition, we have added corresponding references (*Nature*, **643**, 691–698 (2025) as references [33]) to clarify the assignment of the Pt-Pt and Pt-O peaks in Fig. 3f.

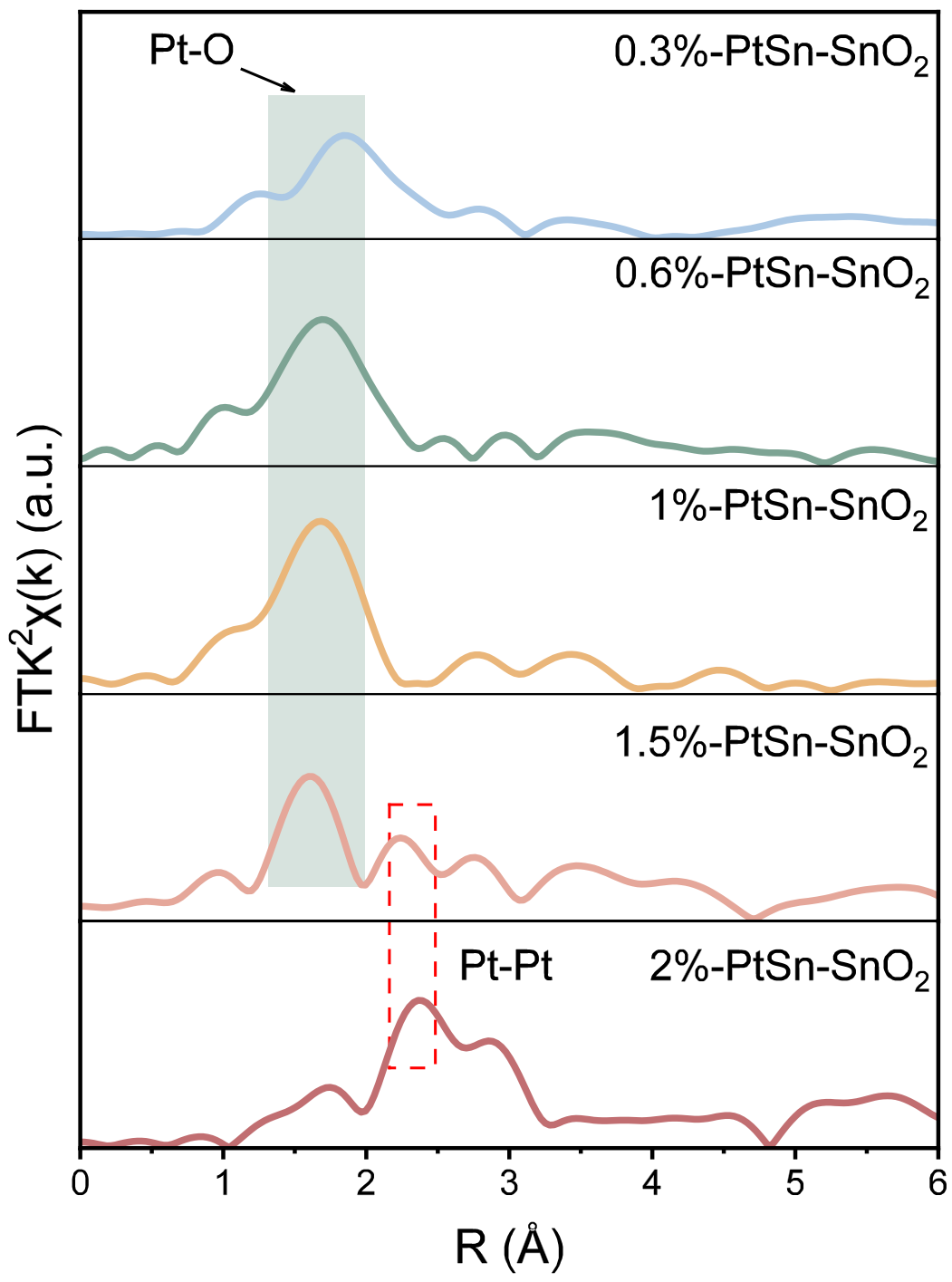

Fig. R9 FT-EXAFS spectra of Pt L_3-edge.

Comments 3: In Fig. 4a, it is unclear whether the figure presents a comparison of the sensor's resistance signal or another type of signal. Additionally, the relationship between color vividness and sensing performance requires further clarification-specifically, whether a more vivid color indicates better sensing performance. It is recommended that the authors provide a detailed explanation.

Reply: We sincerely thank you for your thorough review and valuable comments. We apologize for any ambiguity in the original presentation. Fig. 4a does **not** present the absolute resistance (or response) of the sensors. Instead, it displays the **normalized sensing response** calculated using the **Min-Max scaling method** of the eight sensors toward ten gases at various operating temperatures. Since the baseline response magnitudes vary drastically among the eight different sensors, normalization is essential. It allows for a direct, unbiased comparison of the selectivity patterns and the optimal working windows across the entire sensor array.

The normalization transforms the actual response (S) to a dimensionless value (S_{norm}) ranging from 0 to 1, according to the formula:

$$S_{norm} = \frac{S - S_{min}}{S_{max} - S_{min}} \quad \text{(R-1)}$$

Where S_{max} and S_{min} are the maximum and minimum response values recorded for a specific sensor across all tested gases and temperatures.

Regarding the color vividness, a more vivid color (e.g., red) corresponds to a higher S_{norm} value (closer to 1.0), indicating better relative sensing performance. Cooler colors (e.g., blue/purple) represent lower performance. We employed Min-Max scaling because the eight sensors possess vastly different baseline resistances and response magnitudes. This method eliminates dimensional differences, allowing for a fair, side-by-side comparison of the selectivity patterns across the entire sensor array.

Numerous studies in the field (Adv. Funct. Mater., **35**, 2415517 (2025).) routinely employ this dimensionless normalization to visualize selectivity patterns without the interference of absolute response variations.

Comments 4: In the "Assessment of sensor arrays" section, the authors are encouraged to provide a more detailed description of the experimental procedure for testing interfering gases (e.g., H_2S and SO_2), including parameters such as gas concentration, exposure time, and testing environment.

Reply: Thank you for your kindly reminder. Regarding the testing of interfering gases, we also employed a **static testing method**. Once the sensor resistance stabilized, two/three target gases were simultaneously injected into a 200 mL test chamber. The gas mixtures were extracted from a gas sampling bag using a microliter syringe, ensuring that the time interval between extraction and injection was kept within 5 seconds to maintain consistency.

The amount of injected gas can be also obtained through the equation：

$$C_x = \frac{V_x}{200} \cdot C \qquad \text{(R-2)}$$

All these gas-sensing tests were conducted in an environmentally controlled chamber capable of maintaining constant temperature and humidity, with the test temperature stabilized between 28 °C and the relative humidity (RH) was controlled at 30%.

In response, we have added the following details to the “For the cross-sensitivity and selectivity tests, two/three gases were simultaneously injected into the chamber to simulate complex gas environments. The concentrations of the target gas (NO_2) and interfering gases (e.g., H_2S, SO_2) were calculated based on the injection volume and the total chamber volume (V = 200 mL).” on **Page 8** in Fabrication of MEMS sensors and Gas-sensing measurement section of the supporting information.

Comments 5: On Page 6, the authors synthesized a series of nanofibers via electrospinning technology and stated: "As seen in Figs. S2 and S3, the fibers exhibit average diameters of approximately 200 nm. It is recommended that the authors clarify whether all nanofibers have diameters around 200 nm. Furthermore, an explanation is needed for why different Pt_3Sn doping concentrations do not alter the fiber morphology."

Reply: We are grateful to the reviewer for the valuable comments. We confirm that the average diameter of approximately 200±20 nm applies to all nanofibers about Sn NFs, 0.3%-PtSn-Sn NFs, 0.6%-PtSn-Sn NFs, 1.0%-PtSn-Sn NFs, 1.5%-PtSn-Sn NFs and 2.0%-PtSn-Sn NFs. To clarify this, we have added scale bars and average diameter annotations to all SEM images (Fig.R10).

The reason why varying the Pt_3Sn doping concentrations does not alter the fiber morphology is that the fiber formation is governed by the electrospinning rheology (viscosity and surface tension) of the polymer precursor solution. Since the amount of

Pt_3Sn dopants is extremely low, it does not significantly change the polymer chain entanglement or the solution viscosity. Therefore, the nanofibers retain the same diameter and smooth morphology regardless of the metal loading.

The revised figures are now presented as Fig. S2 in supporting information. And the relevant explanation is added in Page 18 of supporting information: "The introduction of Pt_3Sn alloys does not significantly alter the fiber morphology or diameter. This is attributed to the fact that the electrospinning process is primarily dictated by the rheological properties of the polymer matrix, which remain stable despite the trace amount of metal doping."

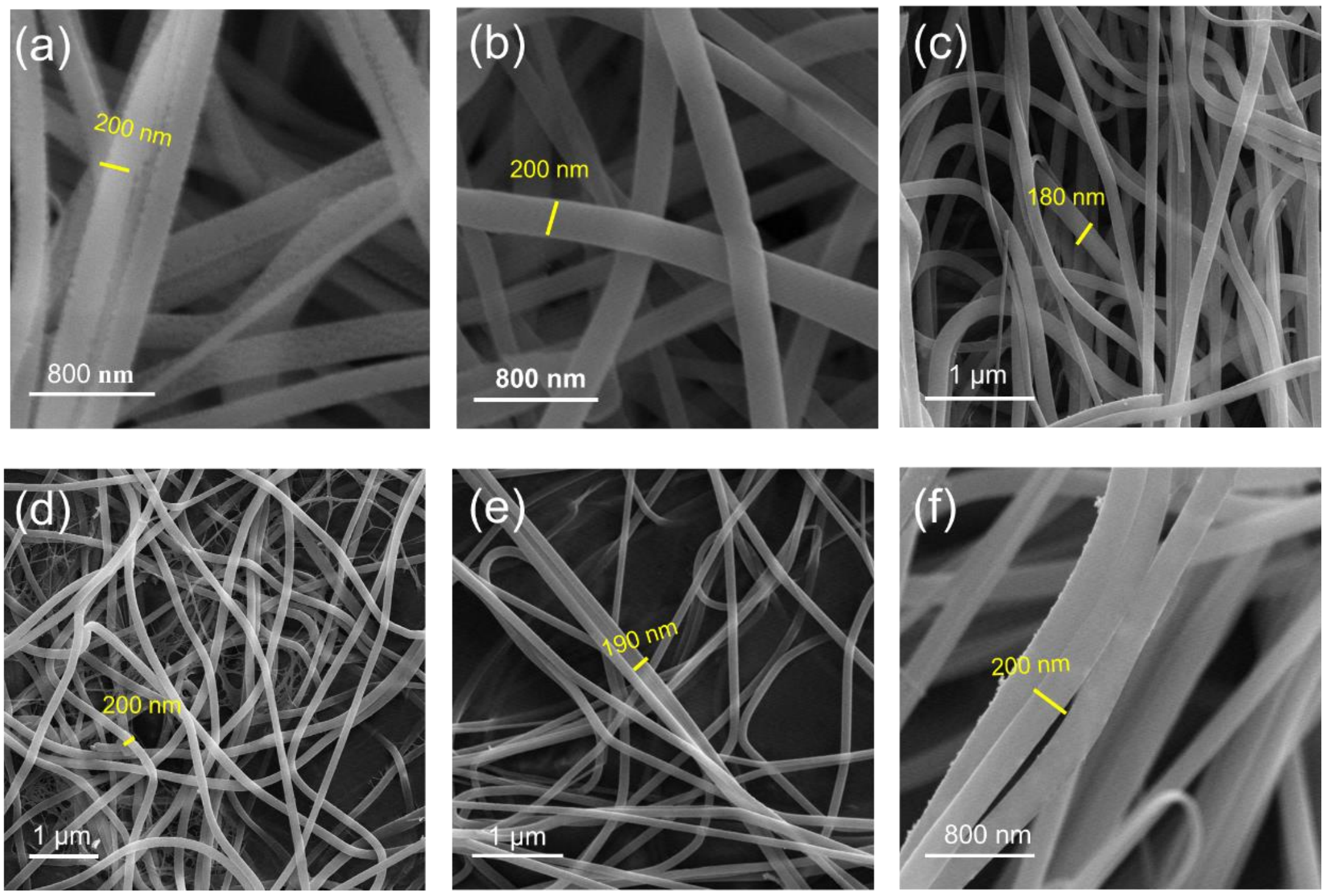

Fig.R10 SEM images of (a) Sn NFs, (b) 0.3%-PtSn-Sn NFs, (c) 0.6%-PtSn-Sn NFs, (d) 1.0%-PtSn-Sn NFs, (e) 1.5%-PtSn-Sn NFs, (f) 2.0%-PtSn-Sn NFs.

After careful reading, although the manuscript presents extensive data on the sensing properties and application, the analysis of the chemical and physical properties of SnO_2-based materials' single-atom modification is insufficient. Furthermore, based solely on the explanations provided in the manuscript, the relationship between these mechanistic descriptions and the adsorption theory used to interpret the concentration dependence of the gas response is inconsistent. To ensure the quality and integrity of this review, I would like to express the following opinions:

1. Please add standard reference patterns for SnO_2 and Pt_3Sn to Fig S7 by marking vertical lines at the bottom of the plot. Include the corresponding standard PDF card numbers in the figure caption.
2. The manuscript claims that Pt species agglomerate into nanoparticles when the Pt_3Sn loading exceeds 1.5 wt% based on Supplementary Fig. S12-S15 Please add quantitative particle size analysis to Fig. S12-S15 and revise the original description with quantitative statistical results.
3. Please add the label "(b)" to the 3D fitting plot in Fig. S18 to match the figure caption description and maintain consistent formatting with other figures.
4. There is a grammatical error in the sentence "promotes the formation of Pt NPs This is conformed to the mapping results in Fig. S15". A period is missing between "Pt NPs" and "This", leading to a run-on sentence.
5. Revise the caption of Figure S31 by changing "towards 10 ppm NO_2 gas" to "towards different concentrations of NO_2 gas (0.1–5 ppm)" to be consistent with the data in the figure.
6. Please label the optimal operating temperature for each sensor and the relative humidity during testing in Fig. 4b.
7. The anti-interference test only includes SO_2, H_2S, and NH_3, which does not cover common interfering gases in practical environments such as CO_2. To better evaluate the practical applicability and selectivity of the sensors, the anti-interference performance should be further verified against these common interfering gases.
8. The optimal working temperature of the best-performing sample 1%-PtSn-SnO_2 (90 ° C) is lower than that of conventional SnO_2-based sensors. Please explain the reason from the physicochemical mechanism.
